# Supplementary material for: Prevalence of HIV seropositive status and associated factors among family members of index cases of antiretroviral clinical attendants in Sodo Town, Southern Ethiopia
Source: PLoS One. 2023 Feb 13;18(2):e0280571. doi: 10.1371/journal.pone.0280571 (PMC9924999; doi:10.1371/journal.pone.0280571)
Supplement: Attachment — Submitted filename: PONE-D-22-04620_R1.pdf [file pone.0280571.s004.pdf]

PLOS ONE

Prevalence Of HIV Seropositive Status and Associated Factors Among Family Members Of Index Cases Of Antiretroviral Clinical Attendants in Sodo Town, Southern Ethiopia

--Manuscript Draft--

**Manuscript Number:**	PONE-D-22-04620R1
**Article Type:**	Research Article
**Full Title:**	Prevalence Of HIV Seropositive Status and Associated Factors Among Family Members Of Index Cases Of Antiretroviral Clinical Attendants in Sodo Town, Southern Ethiopia
**Short Title:**	Prevalence Of HIV Seropositive Status and Associated Factors Among Family Members Of Index Cases of HIV
**Corresponding Author:**	Dereje Haile, MPH/RH<br>Wolaita Sodo University<br>Sodo, SNNPR ETHIOPIA
**Keywords:**	Seropositive status; HIV among biological children; HIV among spousal partners
**Abstract:**	Background<br><br>Human immunodeficiency virus mainly occurs through heterosexual to their partners and vertical to biological children. Therefore partners of people living with HIV and children whose parents with HIV are at higher risk of contracting HIV unless taken preventive measures. This study assessed prevalence of HIV seropositive status and associated factors among family members of index cases of ART clinic attendants in Sodo Town, Southern Ethiopia.<br><br>Methods<br><br>A community-based cross-sectional study was done among 623 randomly selected family members of index cases in Sodo Town from February- June 2021. Data were collected by a pre-tested structural questionnaire. Binary logistic regression was used to investigate variables, independently associated with the outcome variable. The adjusted odds ratio with 95%CI was used to show the strength of association and a P-value <0.05 was used to declare the cut-off point in determining the level of significance.<br><br>Results<br><br>This study revealed that 31.5% with 95%CI (27.6-35.2%) of family members of index cases were HIV seropositive. In subgroup analysis, this study also revealed that 11.1% with 95%CI (8.4-14.5%) of biological children and 69.6% with 95%CI (63.1-75.6%) of spousal partners of index cases were HIV seropositive. Immediate ART initiation of index cases (AOR=0.148, 95%CI: 0.067-0.325), being bedridden or ambulatory functional status at enrollment (AOR=7.71, 95%CI: 3.5-17), baseline CD4 level <350cell/ml (AOR=8.06, 95%CI: 1.8-36) were statistically significant with the outcome variable among biological children. History or symptoms of STIs, (AOR=5.7, 95%CI: 1.86-17.5.), early disclosure (AOR=0.062, 95%CI: 0.024-0.159), immediate ART initiation (AOR=0.172, 95%CI: 0.044-0.675) and duration of infection since known (AOR=5.09, 95%CI: 1.8-14.4) were statistically significant with the outcome variable among spousal partners.<br><br>Conclusion<br><br>HIV among family members of index cases is high as evidenced by our data. Interventions like, immediate ART initiation, early disclosure, screening and early treatment of STI for minimizing HIV transmission should be given.
**Order of Authors:**	Dereje Haile, MPH/RH

Powered by Editorial Manager® and ProduXion Manager® from Aries Systems Corporation

	Alemayehu Kefale
	Kebreab Paulos
	Abdulbasit Sherefa
	Animut Addis
	Amene Abebe
	Muluken Gunta
	Kassa Daka
	Asamino Ayza
**Response to Reviewers:**	Manuscript #: PONE-D-22-04620<br>Title: Seropositive Status and Associated Factors among Family Members of Index Cases of ART clinic Attendants in Sodo Town, Southern Ethiopia<br><br>Comments to the Author<br><br>1. Is the manuscript technically sound, and do the data support the conclusions?<br><br>The manuscript must describe a technically sound piece of scientific research with data that supports the conclusions. Experiments must have been conducted rigorously, with appropriate controls, replication, and sample sizes. The conclusions must be drawn appropriately based on the data presented.<br>Reviewer #1: Yes<br>____<br>2. Has the statistical analysis been performed appropriately and rigorously?<br>Reviewer #1: Yes<br>____<br>3. Have the authors made all data underlying the findings in their manuscript fully available?<br><br>The PLOS Data policy requires authors to make all data underlying the findings described in their manuscript fully available without restriction, with rare exception (please refer to the Data Availability Statement in the manuscript PDF file). The data should be provided as part of the manuscript or its supporting information, or deposited to a public repository. For example, in addition to summary statistics, the data points behind means, medians and variance measures should be available. If there are restrictions on publicly sharing data—e.g. participant privacy or use of data from a third party—those must be specified.<br>Reviewer #1: Yes<br>____<br>4. Is the manuscript presented in an intelligible fashion and written in standard English?<br><br>PLOS ONE does not copyedit accepted manuscripts, so the language in submitted articles must be clear, correct, and unambiguous. Any typographical or grammatical errors should be corrected at revision, so please note any specific errors here.<br>Reviewer #1: No<br>____<br><br>5. Review Comments to the Author<br><br>Please use the space provided to explain your answers to the questions above. You may also include additional comments for the author, including concerns about dual publication, research ethics, or publication ethics. (Please upload your review as an attachment if it exceeds 20,000 characters)<br>General Comments:<br>focused objective and has generated the required data and performed an appropriate analysis of the data and interpretation of the results. It has generated conclusions warranted by the data. However, the language of the submitted manuscript for from adequate, both from the perspective of clearly conveying what is intended and quality standards of scientific publications. Here, review comments are provided that the

	authors may wish to consider improving the quality of the manuscript, particularly in terms of content organization, formatting, and language to make the manuscript potentially publishable in PLOS.<br>Authors need to use the 'PLoS Guidelines to Authors' in the formatting (line spacing, text alignment) as well as in organizing the content in the different sections of the manuscript. Please note that some of the comments given in the different sections below clearly indicate this.<br>The manuscript requires heavy language editing – authors may wish to enlist support from a language editor. Much language editing in the manuscript is done as part of this review (please see below comments in each section)<br>Thank you for your patience and meticulous observation of this manuscript and your point-by-point comments. We have carefully addressed the comments raised accordingly.<br>Specific comments:<br>Title/Title Page<br>The title should be written in sentence case (capitalize only the first word of the title and proper names); expand ART to Antiretroviral Treatment<br>Response: thank you for your comment; we have corrected and highlighted it in the revised manuscript<br>Line 1: Missing space between Seropositiveand Status<br>Response: thank you for your comment; we have corrected and highlighted it in the revised manuscript<br>Line 3: Missing space between words. Correct CLINICATTENDANTSINSODO to Clinic Attendants in Sodo<br>Response: thank you for your comment; we have corrected and highlighted it in the revised manuscript<br>Line 4: Would edit Abebe 2Dereje asread Abebe2, Dereje<br>Response: thank you for your comment; we have corrected and highlighted it in the revised manuscript<br>Line 5: Would remove the extra comma between Addis4 and Muluken<br>Response: thank you for your comment; we have corrected and highlighted it in the revised manuscript<br>Lines 6-9: Would edit the affiliation for each author (include department, University, or organization affiliation, and its location, including city, state/province, and country<br>Response: thank you for your comment; we have corrected and highlighted it in the revised manuscript<br><br>Would add on this page: first author's e-mail address followed by name initials in parenthesis<br>Response: thank you for your comment; we have corrected and highlighted it in the revised manuscript<br>On this page: also indicate authors' contribution(s) using symbols as given in PLoS One "Title, Author, Affiliation Formatting Guidelines".<br><br>Abstract:<br>Lines 30-31: the sentence needs editing; suggest "... transmitted through … sexual contact with an infected partner and babies born to mothers infected with the virus."<br>Response: thank you for your comment; we have corrected and highlighted it in the revised manuscript<br>Lines 33-35: The sentence is the title repeated as is; suggest restating this in a form of an objective ... Like "This study aimed at identifying prevalence and determinants of HIV infection among family members of index cases on antiretroviral treatment (ART)."<br>Response: thank you for your comment; we have corrected and highlighted it in the revised manuscript<br>Line 38: Would Insert HIV between "of" and "index"<br>Response: thank you for your comment; we have corrected and highlighted it in the revised manuscript<br>Line 39: ".. collected data ……" on what ?<br>Response: thank you for your comment; we have corrected and highlighted it in the revised manuscript<br>Line 39: Would add space after the stop after data.<br>Response: thank you for your comment; we have corrected and highlighted it in the revised manuscript<br>Line39: Would replace "investigate" with "identify"

Powered by Editorial Manager® and ProduXion Manager® from Aries Systems Corporation

Response: thank you for your comment; we have corrected and highlighted it in the revised manuscript
Line40: Helpful to add the expanded form for AOR upfront here so that we use the acronym in the rest f the manuscript; i.e., adjusted odds ratio (AOR)
Response: thank you for your comment; we have corrected and highlighted it in the revised manuscript
Line41: Same here. 95% Confidence interval (CI)
Response: thank you for your comment; we have corrected and highlighted it in the revised manuscript
Lines41: The P in P-values is written in upper case as is and italicised (P). Please do this across the manuscript.
Response: thank you for your comment; we have corrected and highlighted it in the revised manuscript

Lines41-42: last part of the sentence requires editing. Suggest changing "----used to declare the cut-off point in determining the level of significance" to "---- used as a cut-off point to determine the level of statistical significance of point estimate."
Response: thank you for your comment; we have corrected and highlighted it in the revised manuscript
Line 45:Would edit"----- revealed that 31.5% with 95%CI (27.6-35.2%)' as "----- revealed that 31.5% (95%CI: 27.6-35.2%); need to use same formant across the manuscript.
Response: thank you for your comment; we have corrected and highlighted it in the revised manuscript.
Lines 46-47: Would edit:(…11.1% with 95%CI (8.4–14.5%..) as (…11.1% (95%CI: 8.4–14.5%)
Response: thank you for your comment; we have corrected and highlighted it in the revised manuscript
Line 47:Would edit:"…and 69.6% with 95%CI (63.1–75.6%..)" as"….. and 69.6% (95%CI: 63.1–75.6%)
Response: thank you for your comment; we have corrected and highlighted it in the revised manuscript
Line 50:Would insert 'of' between "level" and "350"
Response: thank you for your comment; we have corrected and highlighted it in the revised manuscript
Line 51:Would edit "….. significant with …." as "….. significantly associated with …"
Response: thank you for your comment; we have corrected and highlighted it in the revised manuscript
Line 54:Would edit:"…. infection since known …", may remove 'since known' as this does not add an y useful information.
Response: thank you for your comment; we have corrected and highlighted it in the revised manuscript
Line 59:Would change 'should be' to 'might be' or 'need to'; one needs to avoid strong words like should in conclusions/recommendations.
Response: thank you for your comment; we have corrected and highlighted it in the revised manuscript.
Introduction
Line 60: May consider editing as "HIV infection, HIV seropositive, biological children, spousal partners, index case,".
Response: thank you for your comment; we have corrected and highlighted it in the revised manuscript.
Line 63: Definition is incomplete, and the language requires editing. Option: "Index testing is a voluntary process where counsellors or health care providers ask a newly diagnosed HIV positive patient or an HIV positive patient already accessing HIV treatment to list all partners who could have been exposed to HIV through them, the index cases" [1].
Response: thank you for your comment; we have corrected and highlighted it in the revised manuscript.

Line 57: Just use the acronym 'ICT'; already used at first enter above with the expanded forms.
Response: thank you for your comment; we have corrected and highlighted it in the revised manuscript.
Line 67: Insert space " ….models of testing:(1)Facility….." so that it reads" ….models of

	testing (1) Facility….."<br>Line:67:Would edit "… (1)Facility Based ICT …" as " ……. (1) Facility-based ICT …"<br>Response: thank you for your comment; we have corrected and highlighted it in the revised manuscript.<br>Line 68; Would edit "…. index case and gets the names .." to read "…. index cases on ART at the health facility, and gets the names .. "<br>Response: thank you for your comment; we have corrected and highlighted it in the revised manuscript.<br>Line 69: Would replace 'old' with "of age'<br>Response: thank you for your comment; we have corrected and highlighted it in the revised manuscript.<br>Line 69: Would Insert a comma after 'old' and delete "in Antiretroviral Treatment (ART) service giving facility.<br>Response: thank you for your comment; we have corrected and highlighted it in the revised manuscript.<br>Line 69-72: Sentence after '(2)' needs revision, as it has many grammatic errors; suggest: "community-based ICT: a counsellor traces sexual partner(s) of index cases and their biological children who are under 15 years of age through home-to-home visits, offers HTS, and links newly identified People Living with HIV (PLHIV) to ART services [1].<br>Response: thank you for your comment; we have corrected and highlighted it in the revised manuscript.<br>Line 75. Would add 'year' at the end of the sentence.<br>Response: thank you for your comment; we have corrected and highlighted it in the revised manuscript.<br>Line 76. Paragraph starting with this line is a continuation of the above; Would keep this para with the above.<br>Response: thank you for your comment; we have corrected and highlighted it in the revised manuscript.<br>Line 76: Would edit "….. PLHIV were lived and …" as "…. PLHIV were living in 2021, and of which …."<br>Response: thank you for your comment; we have corrected and highlighted it in the revised manuscript.<br>Line 77: In "… transformative and ---" would add 'a' before transformative.<br>Response: thank you for your comment; we have corrected and highlighted it in the revised manuscript.<br>Line 78: Would edit " …. expansion of ART indicating that from total of ….''as "…… as indicated by the total number of …"<br>Response: thank you for your comment; we have corrected and highlighted it in the revised manuscript.<br>Line 79: Would edit "…. sixteen million (16 million) …" as "..16 million…"<br>Response: thank you for your comment; we have corrected and highlighted it in the revised manuscript.<br>Line 79: Would need space between treatment and the citation in"…. on treatment[2] ….".Would do this consistently all along.<br>Response: thank you for your comment; we have corrected and highlighted it in the revised manuscript.<br>Line 80: Would replace "…among which …" by "… of which …."<br>Response: thank you for your comment; we have corrected and highlighted it in the revised manuscript.<br>Line 82: Would keep this paragraph starting with line with the one above paragraph.<br>Response: thank you for your comment; we have corrected and highlighted it in the revised manuscript.<br>Line 82: Would delete 'also"<br>Response: thank you for your comment; we have corrected and highlighted it in the revised manuscript.<br>Line 82.Would replace …among which…." With "… of which …"<br>Response: thank you for your comment; we have corrected and highlighted it in the revised manuscript.<br>Line 83: Would edit "… of age, with 13,000 …" to read " of age. There were 13,000…."<br>Response: thank you for your comment; we have corrected and highlighted it in the revised manuscript.<br>Line 83: Would edit " …. under 15 years old." as "….. under 15 years of age. …."<br>Response: thank you for your comment; we have corrected and highlighted it in the

Powered by Editorial Manager® and ProduXion Manager® from Aries Systems Corporation

	revised manuscript.<br>Line 84: Would edit “PLHIVs” should be “PLHIV”<br>Response: thank you for your comment; we have corrected and highlighted it in the revised manuscript.<br>Line 86: Would merge the paragraph starting with “according to the Ethiopian ….” with the above paragraph<br>Response: thank you for your comment; we have corrected and highlighted it in the revised manuscript.<br>Line 87: Would edit “….was 0.9 percent (0.7 percent ….” as “… was 0.9% (0.7% …..”; and be consistent all along.<br>Response: thank you for your comment; we have corrected and highlighted it in the revised manuscript.<br>Line 88: Would replace “With this general prevalence, the current epidemiology of HIV is heterogeneous, with ….” with 'HIV/AIDS in Ethiopia is regularly categorized as “generalized“ among the adult population, with …….”<br>Response: thank you for your comment; we have corrected and highlighted it in the revised manuscript.<br><br>Line 90: Would replace “…. Geographical ….” with ““…. Geographic ….”<br>Response: thank you for your comment; we have corrected and highlighted it in the revised manuscript.<br>Line 90: Would insert a reference (e.g., the Ethiopian Population-based HIV Impact Assessment 2020) at end of the line (after subgroups)<br>Response: thank you for your comment; we have added and highlighted it in the revised manuscript.<br>Line 92: Would insert after “below 0.1” a reference (e.g., EPHI, Mini EDHS 2018/19).<br>Response: thank you for your comment; we have corrected and highlighted it in the revised manuscript.<br>Line 92: Would replace ‘Somalia” with ‘Ethiopian Somali Region”<br>Response: thank you for your comment; we have corrected and highlighted it in the revised manuscript.<br>Line 97:Paragraph starting with this is redundant, repeating what is covered in the above paragraph. Would delete.<br>Response: thank you for your comment; we have deleted and highlighted it in the revised manuscript.<br>Line 101: Would insert HIV between universal and testing and edit “ …. testing strategy …”, as“…..test and treat strategy ….”<br>Response: thank you for your comment; we have corrected and highlighted it in the revised manuscript.<br>Line 101: Would edit “…. testing all individuals as a mass ……” as“ …. testing all individuals in the population who were at risk …”<br>Response: thank you for your comment; we have edited and highlighted it in the revised manuscript.<br>Line 102: Would edit :…. index case clients are one of these areas [6] …” as“….index cases as a target population for this strategy [6].<br>Response: thank you for your comment; we have edited and highlighted it in the revised manuscript.<br>Line 104: Would keep paragraph starting with this line with the above one.<br>Response: thank you for your comment; we have corrected and highlighted it in the revised manuscript.<br>Line 105: Would edit “….and Family-Based Index Case Testing ….” as“…..Family-based Index Case Testing ……”<br>Response: thank you for your comment; we have corrected and highlighted it in the revised manuscript.<br>Line 105: Would edit “……. or Index Case Testing (ICT) strategy ….” as “……. or the ICT strategy ….”<br>Response: thank you for your comment; we have corrected and highlighted it in the revised manuscript.<br>Line 107: Would edit “…..under-15-year old…” to“…..under 15-year-old…”<br>Response: thank you for your comment; we have corrected and highlighted it in the revised manuscript.<br><br>Line 107: Would edit: “…… biological children to break the chain of ……. “ as “…… biological children. This breaks the chain of ……. “ NB. This breaks a long sentence.

	Response: thank you for your comment; we have corrected and highlighted it in the revised manuscript.<br><br>Line 108: Would edit: "….. who have been exposed to HIV …"as"….. who are exposed to HIV …"<br>Response: thank you for your comment; we have corrected and highlighted it in the revised manuscript.<br>Line 108: Would edit "….. to HIV to give preventive ….." as "to HIV, provide preventive …."<br>Response: thank you for your comment; we have corrected and highlighted it in the revised manuscript.<br>Line 109: Would edit "…… services for negative and, …." as"…… services for those testing HIV-negative, and, ….<br>Response: thank you for your comment; we have corrected and highlighted it in the revised manuscript.<br>Line 109: Would edit "…. if positive, linkage and treatment that increase case detection and attain …." as ". if positive, link them to cared and treatment services, a cascade that will contribute to attaining the.."<br>Response: thank you for your comment; we have corrected and highlighted it in the revised manuscript.<br>Lines 110-111: Would edit "…. while family members have an ongoing risk of contracting HIV." as"….. exposing the family members to an ongoing risk of contracting HIV."<br>Response: thank you for your comment; we have corrected and highlighted it in the revised manuscript.<br>Line 111:Would replace "So" with "Therefore"<br>Response: thank you for your comment; we have corrected and highlighted it in the revised manuscript.<br>Lines 112-113: Would edit "….under 15 years of biological children[1]." as "….under 15-years old biological children[1]."<br>Response: thank you for your comment; we have corrected and highlighted it in the revised manuscript.<br>Line 117: The paragraph starting with this line would rather be kept with the above one.<br>Response: thank you for your comment; we have corrected and highlighted it in the revised manuscript.<br>Line 120-122: Would replace "Testing for HIV and linking to care for those identified by the families of the index cases will improve the health outcomes of the seropositive family members[5]." with "Testing for HIV and linking those who are positive to care and treatment services will improve the health outcomes of the seropositive family members[5].<br>Response: thank you for your comment; we have corrected and highlighted it in the revised manuscript.<br>"Line 123: Would replace 'But' with :Nevertheless'<br>Response: thank you for your comment; we have corrected and highlighted it in the revised manuscript.<br>Line 123: Would edit "HIV positive" as "HIV-positive"; and do this consistently across the manuscript<br>Response: thank you for your comment; we have corrected and highlighted it in the revised manuscript.<br>Lines 123-127: "…people for HIV testing and associated factors among ART clinic attendants at FelegeHiwot Referral Hospital, Northwest Ethiopia, reveals that 25.8% of index cases were not tested. Even though there were a significant number of family members not tested in this study, 30% of family members were seropositive[8]." requires editing; Would consider "……. ART clinic attendants at a referral hospital in Northwest Ethiopia revealed that many children and adults were traced and tested for HIV. Yet, there were many untested partners, children and other individuals living with index cases who were not tested.<br>Response: thank you for your comment; we have edited and highlighted it in the revised manuscript.<br>Lines 128-130: Would delete this para; already addressed in the paragraphs above.<br>Response: thank you for your comment; we have deleted and highlighted it in the revised manuscript.<br>Line131: Paragraph starting with this line would rather be kept with paragraph above ending with ref #8.

	Response: thank you for your comment; we have edited and highlighted it in the revised manuscript.<br><br>Line 131: Would edit "…. no study was conducted …" as"… no similar study was conducted …"<br>Response: thank you for your comment; we have edited and highlighted it in the revised manuscript.<br>'Line 134: Would replace ''would' with "will"<br>Response: thank you for your comment; we have replaced and highlighted it in the revised manuscript.<br>Lines 134-136: " ….. the family members' information in routine clinical practice and could facilitate ending the HIV/AIDS epidemic vision in 2030." requires editing; would suggest: :… 'HIV serostatus information on family members of index cases, which will contribute to improving HIV care and testament services and epidemic control in Ethiopia'.<br>Response: thank you for your comment; we have edited and highlighted it in the revised manuscript.<br>Methods<br>Line 138: Usually written as 'Materials and Methods" or just "Methods"<br>Response: thank you for your comment; we have edited and highlighted it in the revised manuscript.<br>Line 142: Would expand SNNPR. Southern Nations, Nationalities, and Peoples Region (SNNPR).<br>Response: thank you for your comment; we have expanded and highlighted it in the revised manuscript.<br>Line 142: Would edit "… Sodo Town has been…" as"… Sodo Town is …"<br>Response: thank you for your comment; we have edited and highlighted it in the revised manuscript.<br>'Line 143: Would replace '3' with 'three'<br>Response: thank you for your comment; we have replaced and highlighted it in the revised manuscript.<br><br>Line 143: Would edit as " … kebele (lowest administrative entity)"<br>Response: thank you for your comment; we have edited and highlighted it in the revised manuscript.<br>Line 144: Would insert reference for "According to the 2021 population and housing census projection [ ]. …"<br>Response: thank you for your comment; we have edited and highlighted it in the revised manuscript.<br>Line 146: Would replace '3' with 'three'<br>Response: thank you for your comment; we have edited and highlighted it in the revised manuscript.<br>Line 146: Would edit: ".. service-giving facilities …." as".. service-delivery facilities …."<br>Response: thank you for your comment; we have edited and highlighted it in the revised manuscript.<br>Line 147: Would replace '2" with 'two" and '1' with 'one'<br>Response: thank you for your comment; we have edited and highlighted it in the revised manuscript.<br>Line 150: Would replace sentence with "The study was a community-based cross-sectional study"<br>Response: thank you for your comment; we have edited and highlighted it in the revised manuscript.<br>Line 15
**Additional Information:**	
**Question**	**Response**
**Financial Disclosure**<br><br>Enter a financial disclosure statement that describes the sources of funding for the work included in this submission. Review the submission guidelines for detailed	The author(s) received no specific funding for this work.

requirements. View published research articles from *PLOS ONE* for specific examples.<br><br>This statement is required for submission and **will appear in the published article** if the submission is accepted. Please make sure it is accurate.<br><br>**Unfunded studies**<br>Enter: *The author(s) received no specific funding for this work.*<br><br>**Funded studies**<br>Enter a statement with the following details:<br>• Initials of the authors who received each award<br>• Grant numbers awarded to each author<br>• The full name of each funder<br>• URL of each funder website<br>• Did the sponsors or funders play any role in the study design, data collection and analysis, decision to publish, or preparation of the manuscript?<br>• **NO** - Include this sentence at the end of your statement: *The funders had no role in study design, data collection and analysis, decision to publish, or preparation of the manuscript.*<br>• **YES** - Specify the role(s) played.<br><br>* typeset	
**Competing Interests**<br><br>Use the instructions below to enter a competing interest statement for this submission. On behalf of all authors, disclose any competing interests that could be perceived to bias this work—acknowledging all financial support and any other relevant financial or non-financial competing interests.<br><br>This statement is required for submission and **will appear in the published article** if the submission is accepted. Please make sure it is accurate and that any funding sources listed in your Funding Information later in the submission form are also declared in your Financial Disclosure	The authors have declared that no competing interests exist

statement.<br><br>View published research articles from *PLOS ONE* for specific examples.<br><br>**NO authors have competing interests**<br><br>Enter: *The authors have declared that no competing interests exist.*<br><br>**Authors with competing interests**<br><br>Enter competing interest details beginning with this statement:<br><br>*I have read the journal's policy and the authors of this manuscript have the following competing interests: [insert competing interests here]*<br><br>* typeset	
**Ethics Statement**<br><br>Enter an ethics statement for this submission. This statement is required if the study involved:<br><br>• Human participants<br>• Human specimens or tissue<br>• Vertebrate animals or cephalopods<br>• Vertebrate embryos or tissues<br>• Field research<br><br>Write "N/A" if the submission does not require an ethics statement.<br><br>General guidance is provided below. Consult the submission guidelines for detailed instructions. **Make sure that all information entered here is included in the Methods section of the manuscript.**	N/A

**Format for specific study types**<br><br>**Human Subject Research (involving human participants and/or tissue)**<br>• Give the name of the institutional review board or ethics committee that approved the study<br>• Include the approval number and/or a statement indicating approval of this research<br>• Indicate the form of consent obtained (written/oral) or the reason that consent was not obtained (e.g. the data were analyzed anonymously)<br><br>**Animal Research (involving vertebrate animals, embryos or tissues)**<br>• Provide the name of the Institutional Animal Care and Use Committee (IACUC) or other relevant ethics board that reviewed the study protocol, and indicate whether they approved this research or granted a formal waiver of ethical approval<br>• Include an approval number if one was obtained<br>• If the study involved *non-human primates*, add *additional details* about animal welfare and steps taken to ameliorate suffering<br>• If anesthesia, euthanasia, or any kind of animal sacrifice is part of the study, include briefly which substances and/or methods were applied<br><br>**Field Research**<br><br>Include the following details if this study involves the collection of plant, animal, or other materials from a natural setting:<br>• Field permit number<br>• Name of the institution or relevant body that granted permission	
**Data Availability**<br><br>Authors are required to make all data underlying the findings described fully available, without restriction, and from the time of publication. PLOS allows rare exceptions to address legal and ethical concerns. See the PLOS Data Policy and FAQ for detailed information.	Yes - all data are fully available without restriction

Powered by Editorial Manager® and ProduXion Manager® from Aries Systems Corporation

A Data Availability Statement describing where the data can be found is required at submission. Your answers to this question constitute the Data Availability Statement and **will be published in the article**, if accepted.<br><br>**Important:** Stating 'data available on request from the author' is not sufficient. If your data are only available upon request, select 'No' for the first question and explain your exceptional situation in the text box.<br><br>Do the authors confirm that all data underlying the findings described in their manuscript are fully available without restriction?	
**Describe where the data may be found in full sentences. If you are copying our sample text, replace any instances of XXX with the appropriate details.**<br><br>• If the data are **held or will be held in a public repository**, include URLs, accession numbers or DOIs. If this information will only be available after acceptance, indicate this by ticking the box below. For example: *All XXX files are available from the XXX database (accession number(s) XXX, XXX.*).<br>• If the data are all contained **within the manuscript and/or Supporting Information files**, enter the following: *All relevant data are within the manuscript and its Supporting Information files.*<br>• If neither of these applies but you are able to provide **details of access elsewhere**, with or without limitations, please do so. For example:<br><br>*Data cannot be shared publicly because of [XXX]. Data are available from the XXX Institutional Data Access / Ethics Committee (contact via XXX) for researchers who meet the criteria for access to confidential data.*<br><br>*The data underlying the results presented in the study are available from (include the name of the third party*	All relevant data are within the manuscript and its Supporting Information files

Powered by Editorial Manager® and ProduXion Manager® from Aries Systems Corporation

and contact information or URL).<br>• This text is appropriate if the data are owned by a third party and authors do not have permission to share the data.<br><br>* typeset	
Additional data availability information:	

Powered by Editorial Manager® and ProduXion Manager® from Aries Systems Corporation

Manuscript Click here to access/download;Manuscript;Manuscript.docx

PREVALENCE OF HIV SEROPOSITIVESTATUS AND ASSOCIATED FACTORS AMONG FAMILY MEMBERS OF INDEX CASES OF ART CLINICATTENDANTSINSODO TOWN, SOUTHERN ETHIOPIA

Alemayehu Kefale[1], Kassa Daka[2], Amene Abebe [2]Dereje Haile[2], Kebreab Paulos[2], Abdulbasit Sherefa[3], Animut Addis[4], , Muluken Gunta[1]

[1]Wolaita Zone Health Department, Southern Ethiopia

[2]School of Public Health, Wolaita Sodo University, Ethiopia

[3]Deparment of Public Health, Worabe University, Ethiopia

[4]ICAP, Hawassa, Ethiopia

Abstract

Background:

Human immunodeficiency virus is primarily transmitted through heterosexual partners and vertical transmission to biological children. partners of people living with HIV and children whose parents have HIV are at higher risk of contracting HIV unless they take preventive measures. This study assessed the prevalence of HIV seropositive status and associated factors among family members of index cases of ART clinic attendants in Sodo Town, Southern Ethiopia.

Methods:

A community-based cross-sectional study was conducted among 623 randomly selected family members of index cases in Sodo Town from February to June 2021. A pre-tested structural questionnaire was used to collect data.Binary logistic regression was used to investigate variables independently associated with the outcome variable. The adjusted odds ratio with 95%CI was used to show the strength of association, and a P-value 0.05 was used to declare the cut-off point in determining the level of significance.

Results:

This study revealed that 31.5% with 95%CI (27.6-35.2%) of family members of index cases were HIV seropositive. In subgroup analysis, this study also revealed that 11.1% with 95%CI (8.4–14.5%) of biological children and 69.6% with 95%CI (63.1–75.6%) of spousal partners of index cases were HIV seropositive. Immediate ART initiation of index cases (AOR = 0.148, 95%CI: 0.067-0.325), being bedridden or ambulatory functional status at enrollment (AOR = 7.71, 95%CI: 3.5-17), and baseline CD4 level 350 cells/ml (AOR = 8.06, 95%CI: 1.8-36) were statistically significant with the outcome variable among biological children. Among spousal partners, STI history or symptoms (AOR = 5.7, 95%CI: 1.86–17.5), early disclosure (AOR = 0.062, 95%CI: 0.024-0.159), immediate ART initiation (AOR = 0.172, 95%CI: 0.044–0.675), and duration of infection since known (AOR = 5.09, 95%CI: 1.8–14.4) were statistically significant with the outcome variable.

Conclusion:

As evidenced by our data, the risk of HIV among family members of index cases is high. Interventions like immediate ART initiation, early disclosure, screening, and early treatment of STIs for minimizing HIV transmission should be given.

Keywords: Seropositive status, HIV among biological children, HIV among spousal partners.

Introduction

Index case testing (ICT) refers to HIV testing and counseling (HTC) service providers giving counseling for people diagnosed with the Human Immunodeficiency Virus (HIV) and offering HTC to their biological children under 15 years of age and sexual partners.

[1].

Index case testing has two models of testing:(1)Facility Based ICT that a counselor talks with the index case and gets the names and contact information for sexual partner(s) and biological children under 15 years old in Antiretroviral Treatment (ART) service giving facility and (2) Community Based ICT: a counselors trace sexual partner(s) and biological children under 15 years old in the community through home to home visit of index cases house, offer HTS, and links new People Living with Human Immunodeficiency Virus (PLHIV) to ART[1]

According to the United Nations Program on HIV/AIDS (UNAIDS), 37.7 million people worldwide were living with HIV/AIDS in 2021, including 1.7 million children under the age of 15.

[2]. In Eastern and Southern Africa (ESA), 20.6 million PLHIV were lived and 670,000 people were newly infected by HIV[2]. Despite this burden, transformative and remarkable progress has been made in the past decades with regards to the expansion of ART indicating that from a total of PLHIVs in ESA in 2021, sixteen million (16 million) were on treatment[2].

In 2021, in Ethiopia, there were an estimated 620,000 PLHIV, among which 44,000 (7.1%) were children under 15 years of age and 360,000 (62%) were females [3].

There were also 12,000 new annual infections, among which 2,800 were children under 15 years of age, with 13,000 annual deaths (11,000) adults and 2,000 children under 15 years old. Of the total PLHIVs in Ethiopia, 483,127 (465,457 adults and 17,670 children under 15 years of age) were receiving ART[3].

According to the Ethiopian Demographic and Health Survey (EDHS) 2016, the national HIV prevalence in adults aged 15–49 years was 0.9 percent (0.7 percent in males and 1.2% in females)[4].

With this general prevalence, the current epidemiology of HIV is heterogeneous, with a significant variation in the burden of HIV across a geographical area and population subgroups. The urban HIV prevalence is seven times higher than the rural HIV prevalence. Among the regional states, the HIV prevalence ranges from 4.8% in Gambella to below 0.1 in Somalia. There is also a disproportionally high HIV burden among population groups such as sexual partners of PLHIV, female commercial sex workers, long truck drivers, prisoners, widowed and

divorced urban women, and mobile workers, where the HIV prevalence rate is 28%, 23%, 4.9%, 4.2%, 3.5%, and 1.5%, respectively[4, 5].

The epidemic in Ethiopia is concentrated in key populations and priority populations. Female sex workers (FSW) are important populations in Ethiopia.Priority populations are widowed, separated or divorced women; truck drivers; family members of index cases; and people who work in hot spot areas, both mobile and resident[5].

Ethiopia has changed its universal testing strategy, which is testing all individuals as a mass to yield-based target testing strategy. Family members of index case clients are one of these areas [6].

For the implementation, the country has adopted the World Health Organization (WHO) Partner and Family-Based Index Case Testing (PFBICT) or Index Case Testing (ICT) strategy; a voluntary process where counselors and/or healthcare workers ask index cases to list all sexual partners in the past year and under-15-year old biological children to break the chain of transmission by offering HTS to people who have been exposed to HIV to give preventive services for negative and, if positive, linkage and treatment that increase case detection and attain 95-95-95 targets. Some index cases might not have yet disclosed their HIV status, while family members have an ongoing risk of contracting HIV. So, index cases' concerns should be addressed to improve disclosure and testing service uptake among sexual partners and under 15 years of biological children[1].

In Ethiopia, the majority of HIV infections among spousal partners are heterosexual, and 90% of HIV transmission among biological children under the age of 15 is through mother-to-child transmission (MTCT)[4, 7].

This indicates that HIV-negative spousal partners of index cases and biological children under 15 years of age whose parents have HIV or family members of index cases are at a higher risk of contracting HIV. To reduce the risk of HIV transmission, family members of index cases would need to know their HIV status. Testing for HIV and linking to care for those identified by the families of the index cases will improve the health outcomes of the seropositive family members[5].

But a study conducted on the assessment of tracing family members of HIV positive people for HIV testing and associated factors among ART clinic attendants at FelegeHiwot Referral Hospital, Northwest Ethiopia, reveals that 25.8% of index cases were not tested. Even though there were a significant number of family members not tested in this study, 30% of family members were seropositive[8].

Therefore, family members need to be tested, and those who become HIV positive should be linked to care and support services and start treatment. Those who become negative should get appropriate counseling on risk reduction.

However, no study was conducted to assess HIV seropositive status and associated factors among family members of index cases and ART clinic attendants in this town. This study aimed

to assess the prevalence of seropositive status and associated factors among family members of index cases among ART clinic attendants in Sodo Town. The results of this study would emphasize the importance of obtaining the family members' information in routine clinical practice and could facilitate ending the HIV/AIDS epidemic vision in 2030.

METHODS AND MATERIALS

Study Area and Period

The study was conducted at Sodo Town, which is located in Wolaita Zone, South Ethiopia. The Town, which is the capital city of the Zone, is located 380 km south of Addis Ababa, the capital city of Ethiopia, and 157 km south of Hawassa, the capital city of SNNPR. Sodo Town has been further divided into 3 sub-cities and 25 kebele.

According to the 2021 population and housing census projection, Sodo Town has a total population of 244,817, of which 121,184 are males and 123,633 are females. There are two hospitals, three health centers, and 25 health posts. There are 3 ART service-giving facilities in the town, of which 2 are hospitals and the remaining 1 is a health center. This study was conducted from February 18–June 30, 2021.

Study design

A community-based cross-sectional study was conducted.

Population

All family members aged 2–14 years old, biological children and spousal partners of index cases of ART clinic attendants in Sodo Town were considered as the source population.

Study population

All family members aged 2–14 years old, biological children and spousal partners of index cases of ART clinic attendants in Sodo Town

Study participants

Selected family members, biological children aged 2–14, and spousal partners of index cases of ART clinic attendants in Sodo Town

Inclusion and Exclusion criteria

Inclusion criteria

Index cases who had at least one family member aged 2–14 years old were biological children or spousal partners.

Biological children 2–14 years old and spousal partners of index cases who lived in Sodo Town were included.

Exclusion criteria

Index cases who didn't disclose their status

Biological children (2–14 years old) and spousal partners who couldn't respond due to serious illness

Sample size, Sampling technique, and procedure

All of the three (Sodo Health Center, Sodo Teaching and Referral Hospital, and Sodo Christian General Hospital) ART services giving health facilities in the town were included. According to the report of each facility, the total HIV positive clients currently on ART (COA) at the end of September 2020 was 2,317 (Sodo HC = 662, Sodo Teaching and Referral Hospital = 1530, and Sodo Christian General Hospital = 125) and among these 2,213 were adults aged $\geq$18 years old. Primarily from the Partner and Family-Based Index Case testing registration book, we identified all adult clients only living in Sodo Town, and there were 1,144. Secondly, by using HIV testing positive dates between each spousal partner, we identified 834 index cases. Then, from the total index case (834), only 723 who had at least one family member were selected and given a serial code. The ART code numbers of facilities were used to identify each facility. In a study done at public health facilities in Butajira Town, Southern Ethiopia, HIV-positive status disclosure among HIV-positive adult patients attending ART clinics, the magnitude of non-disclosure was 10%[9].

By considering this from 723 index clients, 72 were expected to not disclose and 651 were expected to disclose their status to their family members. Since all index cases were manageable, we included them all and selected them as total index cases. Finally, from each disclosed index case, only one family member was selected by lottery method, for a total of 651 family members from all ART sites.

Study variables

Dependent variable

HIV seropositive status among biological children of index cases

HIV seropositive status among spousal partners of index cases

Independent variables

Socio-demographic and economic variables: - age, sex, residence, marital status, education, wealth status, and occupation.

Index case-related factors: - duration of a relationship, ART initiation status, enrollment status, history of treatment failure, history of adherence, WHO stage, VL, CD4+ and disclosure status

Sexual and substance use behavior of spousal partners: - condom use, substance use (alcohol drinking, chat chewing &tobacco smoking), number of lifetime sexual partnership or multiple sexual partners and type of current marriage (polygamous/monogamous)

HIV testing and STIs history: - ever tested or not, sexually transmitted infections.

Data collection instrument and procedure

The data collection tool was an interviewer-administrator structured questioner developed through the review of related literature. It contains information on socio-demographic and economic factors, sexual and substance use behaviors of spousal partners, HIV testing and STIs-related history factors, and index case-related factors.

After addressing the disclosure issue, data collectors were appointed to index cases with their family members for visiting home. Then, during visiting, the purpose of the study was explained to study participants and informed verbal consent for spousal partners and assent for biological children was taken. Positive serostatus participants were only interviewed, but if their status was unknown or negative, testing and interview were undergone simultaneously. Parents or guardians were interviewed on behalf of biological children. The testing procedure was strictly followed by the current national testing algorithm. For the data of the index case, unique ART numbers were used to review secondary data and link it with the primary data of their family members.

Operational definitions

Index case: - first diagnosed spousal partner with HIV infection in the family

Biological children;-children born from the index case and aged 2-14 years old

Spousal partner;-individuals who are currently married or engaged or in a steady relationship

Family members of index case;-biological children and/or spousal partner

HIV seropositive status: - being reactive as per the national HIV testing algorithmic test

Treatment failure;-clinically stage III or IV, CD4+ <200cells/ml or viral load >/1000 copies/ml after 6 months of ART treatment

Disclosure; Early=when HIV positive family members share his status to his family members within one month, late=if not disclosed within a month or disclosed after a month of diagnosis

Condom use;-always=if used at each sexual intercourse, sometimes=if not used at every sexual intercourse e.g. for only prevention of pregnancy & never=not used at all

ART initiation status; =Immediate, if clients started ART whenever eligible, and late, = if not started ART though eligible to start based on eligibility criteria.

Viral load status; suppressed= if number of copies /ml <1000, and unsuppressed= if number of copies /ml>/1000 after taking ART at least for 6.months in the last 12 months.

Data management and quality control

The English version was translated to the Amharic language and checked for comprehension and consistency. Then it was translated back into English. The Amharic version was used for gathering information. Five percent of the questioners were pre-tested in the Boditi health center, which had similarities with the study population. Then it was modified or contextualized. Eight data collectors and two supervisors working in the ART clinic were recruited and trained. The two-day training addressed the objective of the study, the introduction of the questionnaire, sampling procedure, HIV testing procedure, and interviewing. The data collectors and supervisors participated in pre-testing of the questionnaire for its understanding, which was not included in the study, and the result was used to correct unclear ideas and statements. During data collection, the supervisors and the principal investigator had a close follow-up of the day-to-day data collection process to ensure completeness and consistency of the questionnaire administered each day. After data collection, the collected information was rechecked for its completeness and consistency by the supervisors and principal investigators before being transferred into computer software. A non-overlapping numerical code was given for each questionnaire.

Data analysis procedure

EPI data 4.2 statistical software was used for editing, coding, and data entry, and then exported to SPSS version 25 for cleaning and analysis. A descriptive summary to see the distribution of the study participants with variables under the study was done. Bivariate analysis between dependent and independent variables was performed by using a binary logistic regression model; a P-value of 0.25 was used to select candidate variables for multivariate logistics regression. A VIF or tolerance test was done to check the correlation. Multivariate logistic regression analysis was done to adjust for possible confounding variables. A P-value of 0.05 was used as a cut-off point. An Adjusted Odds Ratio (AOR) with a 95% confidence interval (CI) was used to determine the level of association. The Hosmer and Lemeshow's goodness of fit test was used to check the model's fitness. Results were presented in narrative and tables.

Ethical consideration

Ethical clearance was obtained from the Ethical Review Board of Wolaita Sodo University College of Health Science and Medicine. Formal permission letters for each health facility were written by the Zonal Health Department and Town Health Office in decentralized order. The purpose of the study was explained to the study participants and both informed written and verbal consent from each spousal partner and assent for biological children from their parents were obtained. The information obtained from the study participants was kept confidential. The testing and interview were undertaken privately in a separate area. Family members who tested negative were counseled about further preventive measures, and positives were linked to a nearby ART clinic where care and support were given according to the national guidelines.

RESULTS

Socio-demographic and economic characteristics of family members of index cases

A total of 623 family members of index cases in Sodo Town were interviewed with a response rate of 94%. While the majority were biological children (406, 65.2%), nearly one-third (217, 34.8%) were spousal partners. The mean age of biological children was 8.9 $\pm$ 3.66 and the spousal partner was 36.9 $\pm$ 9.26 years. Of the respondents, nearly half (51.5%) were females, more than half (54.6%) were Orthodox Christians, and one-third (32.6%) had Frist percentile wealth status. Including 1072 biological children (2–14) years old (1.72 biological children per index case), a total of 2,932 family members (4.71 family members per index case) were identified from 623 index cases (Table 1).

Table 1 Socio-demographic and economic characteristics of family members of index case in Sodo Town, Southern Ethiopia, 2021(n=623).

Variables	Category	Frequency	Percentage

Sex	Male<br>Female	302<br>321	48.5<br>51.5
Age	<5<br>5-9<br>10-14<br>15-29<br>>=30	87<br>126<br>192<br>43<br>175	14.0<br>20.2<br>30.8<br>6.9<br>28.1
Religion	Orthodox<br>Protestant<br>Muslim<br>Catholic	340<br>249<br>26<br>8	54.6<br>40.0<br>4.2<br>1.3
Wealth index	Frist percentile<br>Secondpercentile<br>Thirdpercentile	203<br>211<br>209	32.6<br>33.9<br>33.5
Family size	1-3<br>4-6<br>>=7	129<br>447<br>47	20.7<br>71.7<br>7.5
2-14 yrs. old biological child size	<3<br>>=3	506<br>117	81.2<br>18.8
Alive parents	Father<br>Mother<br>Both	26<br>123<br>474	4.2<br>19.7<br>76.1
Relation to the index	Spouse<br>Biological child	217<br>406	34.8<br>65.2
Even though we expected651 index case disclosures from 723 eligible index cases, to determine sample size, 662 were disclosed during data collection and used for to determine non-response rate.			

Sexual and substance use behavior of spousal partners of index case

Nearly one-fourth (23%) had 10 or more lifetime sexual partners. The majority (84.8%) were in a monogamous relationship; 69.1% had been living together with their spouse for more than ten years; and (68.2%) had an age difference of ten years or more between spousal partners.Despite the fact that most (76.5%) had been educated on condom use, more than half (60.8%) had never used one during sexual intercourse with their spousal partner.While the majority had never smoked tobacco (78.8%) and chaw chat (70%), 9.7% drank alcohol daily (Table 2).

Table 2 Sexual and substance use behavior of spousal partners of index case in Sodo Town, Southern Ethiopia, 2021(n=217)

Variables	Category	Frequency	Percentage
Number of life time sexual partners	<5	89	41.0
	5-9	78	35.9
	=/>10	50	23.0
How do you describe the type of your current marriage	Polygamous	33	15.2
	Monogamous	184	84.8
Is this your first marriage or partnership	Yes	28	12.9
	No	189	87.1
Are you the first spouse for your spouse	Yes	17	7.8
	No	200	92.2
Duration of spousal partners living together in year	<5	26	12.0
	5-9	41	18.9
	>=10	150	69.1
Age difference between spousal partners	<5	22	10.1
	5-9	47	21.7
	>=10	148	68.2
Ever discussed condom use with your spousal partner	Yes	166	76.5
	No	51	23.5
Ever used a condom during sexual intercourse with your spousal partner	Yes	98	45.2
	No	119	54.8

How regularly did you use condoms during sexual intercourse with spousal partner	Never<br>Sometimes<br>Always	132<br>72<br>13	60.8<br>33.2<br>6.0
Ever drunk alcohol	Never<br>Occasionally<br>Daily	42<br>154<br>21	19.4<br>71.0<br>9.7
Ever used Tobacco	Never<br>Occasionally<br>Daily	171<br>26<br>20	78.8<br>12.0<br>9.2
Ever chaw chat	Never<br>Occasionally	152<br>65	70.0<br>30.0

HIV testing history and serostatus of family members of index cases

Most family members (90.9%) had ever been tested, and 57 (9.1%) had not been tested. Of the ever-tested participants, 186 (32.9%) had positive status. Of those who had never been tested or whose results were negative (437), 10 (2.8%) were newly identified positives. The overall prevalence among family members was 31.50% (196 out of 623 tested and known results). (Table 3)

Table 3: HIV serostatus and testing history-related factors of family members of index cases in Sodo Town,Southern Ethiopia, 2021

Variables	Category	Frequency	Percentage
Have you ever tested for HIV	Yes<br>No	566<br>57	90.9<br>9.1
What was the result if you had been tested(n=566)	Positive<br>Negative	186<br>380	32.9<br>67.1
If never been tested or the result was negative, what is the current result? (practically confirmed)(n=437)	Positive<br>Negative	10<br>427	2.29<br>97.71
Final current HIV serostatus	Positive<br>Negative	196<br>427	31.5<br>68.5

In a sub-group analysis of only biological children, 353 (86.9%) have ever been tested, and 53 (13.1%) have not been tested. Of the ever-tested participants, 37 (10.5%) had positive status. Of those who had never been tested or whose results were negative, eight (2.8%) were newly identified positives. Of all the biological child respondents, 45 (11.1%) were positive and 361 (88.9%) were negative. (Table 4)

Table 4:HIV serostatus and testing history of biological children of index cases, Sodo Town, Southern Ethiopia,2021 (n=406)

Variables	Category	Frequency	Percentage
Have you ever tested for HIV	Yes<br>No	353<br>53	86.9<br>13.1
What was the result if you had been tested(n=353)	Positive<br>Negative	37<br>316	10.5<br>89.5
If never been tested or the result was negative, what is the current result? (practically confirmed)(n=369)	Positive<br>Negative	8<br>361	2.17<br>97.83
Final current HIV serostatus	Positive<br>Negative	45<br>361	11.1<br>88.9

In a sub-group analysis of only spousal partners, nearly one-fourth (23%) had STI symptoms or a history of STI in the 12 months prior to data collection.All (100%) heard about HIV testing and counseling service provision and thought testing is important to family members of PLHIVs. The majority (98.2%) have been tested, while four (1.8%) have not.ever-tested participants, 149 (70%) had positive status. Of those who had never been tested or whose results were negative, 2 (3%) were newly identified positives. From all the spousal partner respondents, 151 (69.6%) were positive and 66 (30.4%) were negative. (Table 5)

Table 5:STIs and HIV testing history of spousal partners of index cases in Sodo Town, Southern Ethiopia, 2021 (n=217)

Variables	Category	Frequency	Percentage
had history or symptoms of STIs	Yes<br>No	50<br>167	23.0<br>77.0
Heard about HIV testing and counseling service provision to family members PLHIVs	Yes<br>No	217<br>0	100<br>0

Do you think testing is important to family members of PLHIVs	Yes<br>No	217<br>0	100<br>0
Have you ever tested for HIV	Yes<br>No	213<br>4	98.2<br>1.8
What was the result if you had been tested(n=213)	Positive<br>Negative	149<br>64	70.0<br>30.0
If never been tested or the result was negative, what is the current result? (practically confirmed)(n=68)	Positive<br>Negative	2<br>66	2.94<br>97.06
Final current HIV serostatus	Positive<br>Negative	151<br>66	69.6<br>30.4

Characteristics of index cases of family members

From a total of 623 index cases, 386 (62%) disclosed their status to at least one family member within a month of diagnosis, and the majority of 476 (76.4%) started ART immediately as they were eligible. Most (97% and 79.9%) of the index cases had current and at enrollment working functional status, respectively. Five hundred fifty eight (89.6%) had a current suppressed viral load and 330 (53%) had a low CD4 count (350 cells/ml) at ART enrollment. The majority (505, 81.1%) and (406, 65.2%) of the index cases had no history of treatment failure and poor adherence, respectively. (Table 6)

Table 6: Index case-related factors of family members of index cases, Sodo Town, Southern Ethiopia, 2021

Variables	Category	Frequency	Percentage
Notification of disclosure of HIV status of index cases	>/1month<br><1month	237<br>386	38.0<br>62.0
Immediate ART initiationof index cases	Yes<br>No	476<br>147	76.4<br>23.6
Functional status at enrollmentof index cases	Bedridden<br>Ambulatory<br>Working	40<br>85<br>498	6.4<br>13.6<br>79.9
Current functional statusof index cases	Bedridden<br>Ambulatory	5<br>14	0.8<br>2.2

	Working	604	97
WHO stage at enrollmentof index cases	Stage One<br>Stage Two<br>stage Three<br>stage Four	265<br>167<br>152<br>39	42.5<br>26.8<br>24.4<br>6.3
Current WHO stageof index cases	Stage One<br>Stage Two<br>Stage Three<br>stage Four	593<br>25<br>0<br>5	95.2<br>4.0<br>0<br>0.8
Duration of infection since knownof index cases	<60 month<br>60-120 month<br>>/120 month	164<br>189<br>270	26.3<br>30.3<br>43.3
Time to start ART since known infectionof index cases	<=1 month<br>2-11 month<br>>=12 month	254<br>235<br>134	40.8<br>37.7<br>21.5
Base line CD4of index cases	<350 cells/ml<br>>/350 cells/ml	330<br>293	53.0<br>47.0
Current Viral Load(VL) statusof index cases	Unsuppressed<br>Suppressed	65<br>558	10.4<br>89.6
History of treatment failureof index cases	Yes<br>No	118<br>505	18.9<br>81.1
History of poor adherenceof index cases	Yes<br>No	217<br>406	34.8<br>65.2

Associated factors with HIV seropositive status among biological children

Immediate ART initiation, if eligible, functional status at enrollment, WHO stage at enrollment, duration of infection since known and baseline CD4 of index cases were found a candidate significantly associated variable for a seropositive status of biological children of index case and their AOR was calculated after these five variables entered together in binary logistic regression model (Table 7).

Multivariate analysis in binary logistic regression found that three out of five variables were significantly associated with the outcome variable. Biological children whose eligible index cases started ART immediately were 85% less likely to be seropositive than those who didn't (AOR = 0.148; 95%CI: 0.067-0.325). Biological children whose index cases' functional status was bedridden or ambulatory at enrollment were 7.71 times more likely to be seropositive than those whose status was working (AOR = 7.71; 95%CI: 3.5-17). Biological children whose index case baseline CD4 level was 350 cells/ml were 8.06 times more likely to be seropositive than those whose CD4 level was > 350 cells/ml (AOR = 8.06; 95%CI: 1.8-36)(Table 7).

Table 7: Factors associated with seropositive status among biological children of index cases in Sodo Town, 2021 (n=406)

Variables	Category	Serostatusof biological children		95%CI	
		Positive	Negative	COR	AOR
Immediate ART initiation of index cases	Yes	13(3.2%)	300(73.9%)	0.083(0.041-0.167)	0.148(0.067-0.325)***
	No	32(7.9%)	61(15.0%)	1	1
Functional status at enrollment of index cases	Bedridden or Ambulatory	32(7.9%)	51(12.6%)	14.96(7.36-30.4)	7.71(3.5-17)***
	Working	13(3.2%)	310(76.4%)	1	1
WHO stage at enrollmentof index cases	Stage1 or 2	10(2.5%)	276(68.0%)	1	1
	Stage3 or 4	35(8.6%)	85(20.9%)	11.7(5.4-23.9)	1.53(0.512-4.55)
Duration of infection since knownof index cases	<60 month	3(0.7%)	95(23.4%)	1	1
	60-120 month	13(3.2%)	121(29.8%)	3.4(0.942-12.3)	0.597(0.119-2.99)
	>/120 month	29(7.1%)	145(35.7%)	6.33(1.88-21.4)	0.853(0.181-4.02)
Base line CD4 of index cases	<350	43(10.6%)	167(41.1%)	24.98(5.96-104)	8.06(1.8-36)**

	>/350	2(0.5%)	194(47.8%)	1	1
* Statistically significant (p=0.01–<0.05), ** Strong statistical significance (p=0.001–<0.01), *** Very strong statistical significance (p< 0.001)					

Associated factors with HIV seropositive status among spousal partners

The bivariate analysis showed sex, wealth index, ever used a condom, had a history or symptoms of STI in the 12 months before the survey among spousal partners and factors from the index cases such as; notification of disclosure status of HIV, ART initiation, if eligible, functional status at enrollment, WHO stage at enrollment and duration of infection since known were candidate variables for multivariable binary logistic regression analysis. In multivariable logistic regression analysis, had a history or symptoms of STI, and from the index factors like notification of disclosure of HIV status, ART initiation status, functional status at enrollment, WHO stage at enrollment, and duration of infection since known were factors significantly associated with HIV seropositive status of spousal partners of index cases. Spousal partners who had a history or symptoms of STI in the previous 12 months were 5.7 times more likely to be seropositive than their counterparts (AOR = 5.7; 95% CI: 1.86–17.5).Spousal partners whose index cases disclosedtheir status within a month of diagnosis were 94% less likely to be seropositive than those whose index cases stayed a month or more (AOR=0.062; CI: 0.024-0.159).Spousal partners whose index cases started ART immediately as eligible were 83% less likely to be seropositive than those whose index cases started immediately (AOR = 0.172; 95%CI: 0.044–0.675). Spousal partners whose index cases were WHO stage III or IV at enrollment were 7.27 times more likely to be seropositive than those whose index cases were WHO stage I or II (AOR = 7.27; 95%CI: 2.37–22.3). Spouses of index cases who had known their status for more than 10 years were 5.9 times more likely to be seropositive than those who had known their status for less than 5 years (AOR = 5.9; 95%CI: 1.8–14.4)(Table8).

Table 8: Factors associated with seropositive status among spousal partners in Sodo Town, 2021 (n=217)

Variables	Category	Serostatus of spousal partner		95%CI	
		Positive	Negative	COR	AOR
Sex	Male	76(35.0%)	42(19.4%)	1	1
	Female	75(34.6%)	24(11.1%)	1.73(0.953-3.13)	1.84(0.75-4.52)
Wealth index	Fristpercentile	46(21.2%)	23(10.6%)	0.645(0.317-	0.551(0.193-

				1.31)	1.57)
	Secondpercentile	43(19.8%)	23(10.6%)	0.603(0.295-1.23)	0.345(0.125-1.949)
	Thirdpercentile	62(28.6%)	20(9.2%)	1	1
Ever used a condom	Yes	59(27.2%)	39(18.0%)	1	1
	No	92(42.4%)	27(12.4%)	2.25(1.25-4.06)	1.37(0.561-3.36)
Had or symptoms of an STI	Yes	41(18.9%)	9(4.1%)	2.36(1.07-5.2)	5.7(1.86-17.5)**
	No	110(50.7%)	57(26.3%)	1	1
Notification of Disclosure of index cases	>/1month	106(48.8%)	10(4.6%)	1	1
	<1month	45(20.7%)	56(25.8%)	0.076(0.036-0.162)	0.062(0.024-0.159)***
Immediate ART initiationof index cases	Yes	100(46.1%)	63(29.0%)	0.093(0.028-0.312)	0.172(0.044-0.675)*
	No	51(23.5%)	3(1.4%)	1	1
Functional status at enrollment of index cases	Bedridden or Ambulatory	40(18.4%)	2(0.9%)	11.5(2.7-49.3)	1.23(0.198-7.68)
	Working	111(51.2%)	64(29.5%)	1	1
WHO stage at enrollment of index cases	Stage1or2	86(39.6%)	60(27.6%)	1	1
	Stage3or4	65(30.0%)	6(2.8%)	7.56(3.08-18.6)	7.27(2.37-22.3)**
Duration of infection since knownof index cases	<60 month	31(14.3%)	35(16.1%)	1	1
	60-120 month	40(18.4%)	15(6.9%)	3(1.4-6.47)	3.04(0.994-9.32)
	>/120 month	80(36.9%)	16(7.4%)	5.65(2.74-11.6)	5.09(1.8-14.4)**

* Statistically significant (p=0.01–<0.05), ** Strong statistical significance (p=0.001–<0.01), *** Very strong statistical significance (p < 0.001).

DISCUSSION

In this study, the seropositive status among family members was (31.5%) with a 95%CI of (27.6-35.2%). This implies that family members of the index case are at high risk of HIV infection. The prevalence is consistent with the study conducted at Felege Hiwot Referral Hospital, Northwest Ethiopia, which was 30%,[8] and Zimbabwe 32.6%[10]. However, it is lower than a study conducted in South Africa 38% [11]. Moreover, it is higher than the prevalence of 22% in a study done in Malawi [12].

The possible explanations for this difference might be due to variation in socio-demographic and economic characteristics, prevention strategies, and implementation differences across the nations.

In a sub-group-analysis of the prevalence of seropositive status among family members of index cases only on biological children, the prevalence of seropositive status in our study area was found to be 11.1% with a 95%CI of (8.4–14.5%). This finding is consistent with a study done at FelegeHiwot Referral Hospital, Northwest Ethiopia, which found 10.7%[8].

But our finding is much higher compared with other studies, including the Lesotho study that showed a prevalence of 1.4%, Malawi at 4%, and Kenya at 7.4% [13-15].

Possible explanations for this difference might be implementation differences (time of implementation, service coverage) of the prevention of mother-to-child transmission (PMTCT) strategy and socio-demographic and economic differences between nations.

In a sub-group-analysis of the prevalence of seropositive status among family members of index cases only on spousal partners, in our study area, was found to be 69.6% with a 95%CI (63.1-75.6%).

This finding is almost similar to a study done at FelegeHiwot Referral Hospital, Northwest Ethiopia; which was 71.5%[8]. But, this finding is lower than in a study conducted in SSA 76.23%[16]. On the other side, this finding is higher than a study conducted in Ethiopia, EDHS, 2016 28%[4].

The possible explanation for this wide gap with the national prevalence might be that participants of index cases were the only new index cases in EDHS, 2016. Likewise, this finding is also higher than the study conducted on 37.7% in Nigeria and 61.9% in Tanzania [17, 18].

The possible explanations for this difference might be ART service coverage, prevention, implementation, and monitoring strategy differences from one country to another country.

This finding revealed that biological children whose index cases started ART immediately, based on eligibility criteria, were 85% less likely to be seropositive than those whose index cases didn't start. This might be the fact that initiation of ART increases CD4 and inversely decreases the viral load level of index cases, eventually preventing transmission of HIV to their biological children[7].

This study showed that biological children whose index cases were bedridden or ambulatory functional status at enrollment were 7.71 times more likely to be seropositive than those whose status was working. This finding also revealed that biological children whose index case baseline CD4 level was 350 cells/ml were 8.06 times more likely to be seropositive than those whose CD4 level was > 350 cells/ml. Even though there is no study on these specific age groups and almost 90% of biological children acquire the infection through vertical transmission from infected mother to child, these factors are supported by the national PMTCT guideline of Ethiopia [7]. As index cases do not start ART as per the eligibility criteria, the CD4 level declines and lead to viral replication which exposes to different opportunistic infections that finally become a client functional status deterioration and vertical viral transmission to a biological child.

This study revealed that spousal partners who had a history or symptoms of an STI in the 12 months before the survey were 5.7 times more likely to be seropositive than their counterparts. It is consistent with the study conducted in 30 provinces of China [19].

The possible explanation might be that since STI and HIV have a linear relationship, and STI may be characterized by genital sores or ulcers, inguinal swelling that is exposed to HIV transmission. This finding is supported by EDHS, 2016 [4].

This finding showed that spousal partners whose index cases disclosed their status within a month of diagnosis were 94% less likely to be seropositive than those whose index cases stayed a month or more after diagnosis. This prevention potential implies that disclosure to spousal partners facilitates discussion on HIV and raises spousal partner awareness about their risk and need to test. Disclosure also has an important health benefits. It increases social support, fosters closer relationship to others, increases testing uptake, create good prevention opportunities like, condom utilization, and other prevention methods, improves treatment adherence and retention, which increases CD4 and suppressed viral load level that leads to halting of transmission. This finding is supported by the National Comprehensive HIV Care Guideline of Ethiopia, 2018[6].

This finding revealed that those spousal partners whose index cases started ART immediately as eligible by eligibility criteria were 83% less likely to be seropositive than those whose index partners didn't start. This finding is consistent with a study conducted in Addis Ababa, Ethiopia [20].

This might be since ART initiation based on the eligibility criteria leads to the index case CD4 increase and viral load level suppression that decreases transmission to his or her spousal partner.

This study showed that spousal partners whose index cases had WHO status stage III or IV at enrollment were 7.25 times more likely to be seropositive than spousal partners whose index cases were WHO stage I or II. It is consistent with the study conducted in Thailand and Northern Vietnam[21, 22].

This is due to the fact that those who were stage III or IV had severe opportunistic infections that exposed the index case to decreased CD4 levels and increased viral load levels, which led to high viral transmission to spousal partners.

This study revealed that spousal partners whose index case lived with the virus since they knew their status for more than 10 years were 5.09 times more likely to be seropositive than those who knew their status for less than 5 years. It is consistent with the study conducted in Thailand and Rakia, Uganda [21, 23].

This implies that as the duration of the relationship between spousal partners increases, the rate of HIV transmission also increases.

Strength and Limitation of the Study

Since the factors of biological children and spousal partners of index cases are different, a separate sub-group analysis was the strength of this study.

Family members whose index cases hadn't disclosed their status or had follow-up outside of Sodo Town were not included in this study. Obstetric, labor, delivery, and infant factors, as well as other modes of transmission other than MTCT, were excluded as biological child factors.

CONCLUSION

In this study, HIV among family members of index cases in general and specifically in both biological children and spousal partners in Sodo Town is high..late ART initiation as per the eligibility criteria, bedridden or ambulatory functional status during enrollment, and baseline CD4 350 cells/ml of index cases were the major factors of seropositive among biological children of index cases. Whereas among spousal partners with HIV-history or symptoms of STI, and from the index case side, late notification of disclosure of status, late ART initiation as per the eligibility criteria, WHO stage III or IV at enrollment, and duration of HIV infection since known > 10 years were factors for the seropositive. So, in the context of the town, holistic efforts to promote the prevention of HIV among family members, including index cases, are critical to ending the HIV epidemic.

ACKNOWLEDGMENT

We are grateful to the Wolaita Sodo University, College of Medicine and Health Sciences for providing ethical clearance for this study. We are also indebted to our data collectors, supervisors, and the study participants.

Funding: no funding was received.

Author's contribution

Conceptualization: Alemayehu Kefale

Data curation: Alemayehu Kefale, Dereje Haile, Amene Abebe, Kassa Daka

Formal analysis: Alemayehu Kefale, Dereje Haile, Amene Abebe, Abdulbasit Sherefa

Investigation: : Alemayehu Kefale, Dereje Haile, Amene Abebe, Abdulbasit Sherefa, Animut Adiss, Muluken Gunta, Kassa Daka

Methodolgy: Alemayehu Kefale, Dereje Haile, Amene Abebe, Abdulbasit Sherefa, Animut Adiss, Muluken Gunta, Kassa Daka

Project Administration: Alemayehu Kefale, Animut Adiss, Muluken Gunta

Resources: Alemayehu Kefale, Animut Adiss, Muluken Gunta

Software: Alemayehu Kefale, Dereje Haile, Amene Abebe, Abdulbasit Sherefa, Kassa Daka

Supervision: Alemayehu Kefale, Dereje Haile, Amene Abebe, Abdulbasit Sherefa, Animut Adiss, Muluken Gunta

Validation: Alemayehu Kefale, Dereje Haile, Amene Abebe

Visualization: Alemayehu Kefale, Dereje Haile, Amene Abebe

Writing original draft: Alemayehu Kefale, Dereje Haile

Writing – review & editing: Alemayehu Kefale, Dereje Haile, Amene Abebe, Abdulbasit Sherefa, Animut Adiss, Muluken Gunta

References

1. WHO, *Partner and Family-Based Index Case Testing: A Standard Operating Procedure (SOP)* 2015: p. 3-20.
2. UNAIDS, *FACT SHEET 2021 Global Hiv Statistics.* Ending the AIDS epidemic, 2021(June): p. 1-3.
3. info, U.a.A., *Country factsheets, Ethiopia* Unaids, 2021: p. 1-6.
4. Agency, C.S., *Ethiopian Demographic and Health Survey(EDHS), 2016, HIV Report*. 2018.
5. HAPCO, *HIV Prevention in Ethiopia National Road Map 2018 - 2020.* 2018. **https:// e**(November 2018): p. 25-54.
6. FMOH, E., *National Consolidated Guidelines for Comprehensive Hiv Prevention , Care and treatment.* 2018: p. 1-238.

7. MOH, *National Comprehensive and Integrated Prevention of Mother-Child Transmission of HIV Guideline* 2018. **6**(1).
8. Belayneh, T.W., *Assessment of Tracing Family Members of HIV Positive People for HIV Testing and Associated Factors among ART Clinic Attendants at Felege Hiwot Referral Hospital, Northwest Ethiopia.* Journal of Pharmacy and Alternative Medicine, 2017. **15**: p. 16-28.
9. Tamirat Melis Berhe, L.L., Addissu Alemayehu, Desalegn Ajema, Mustefa Glagn, Samuel Dessu, *HIV-Positive Status Disclosure and Associated Factors among HIV-Positive Adult Patients Attending Art Clinics at Public Health Facilities of Butajira Town Southern Ethiopia.* AIDS Research and Treatment, 2020. **2020**.
10. Nyikadzino Mahachi, A.M., Taurayi A Tafuma, Peter Mawora, Liz Kariuki, Bazghina-werq Semo,Moses H Bateganya, Tendai Nyagura, Getrude Ncube, Mike B Merrigan, Otto N Chabikuli and Mulamuli Mpofu, *Sustained high HIV case-finding through index testing and partner notification services: experiences from three provinces in Zimbabwe.* the International AIDS Society, 2019. **22**(S3): p. 23-30.
11. Dvora Joseph Davey, K.M.W., Claire Serrao, Marlien Prins, Madaline Feinberg, Ntokozo Mtonjana, Khanyo Hlophe, Lindiwe Zuma, Senate Sejake, and Todd Malone, *HIV Positivity and Referral to Treatment Following Testing of Partners and Children of PLHIV Index Patients in Public Sector Facilities in South Africa.* Journal of Acquired Immune Deficiency Syndromes, 2019. **81**(4): p. 365-370.
12. MALAWI: Improvement, Q.S., F O R, *INDEX CASE TESTING: A PROMISING STRATEGY FOR ACHIEVING HIV EPIDEMIC CONTROL.* Management Sciences for Health, 2018(June): p. 7-10.
13. Makhahliso Jubilee, F.J.P., Knowledge Chipango, Kenoakae Pule,Albert Machinda, Noah Taruberekera, *HIV index testing to improve HIV positivity rate and linkage to care and treatment of sexual partners, adolescents and children of PLHIV in Lesotho.* PLoS ONE, 2019. **14**(3): p. 1-12.
14. Saeed Ahmed, R.A.S., Katie Simon, Nora E. Rosenberg, Elijah Kavuta, Mwelura Harawa, Spencer Dick, Frank Linzie, Peter N. Kazembe and Maria H. Kim, *Index case-finding facilitates identification and linkage to care of children and young adults living with HIV/AIDS in Malawi.* Tropical Medicine and International Health, 2019. **22**(8): p. 1021-1029.
15. Anjuli D WAGNER, C.M.W., Irene N NJUGUNA, Elizabeth MALECHE-OBIMBO, Kenneth SHERR, Irene W INWANI, James P HUGHES, Dalton C WAMALWA, Grace C JOHN-STEWART, and Jennifer A SLYKER, *Active referral of children of HIV-positive adults reveals high prevalence of undiagnosed HIV.* Journal of Acquired Immune Deficiency Syndromes, 2016. **73**(5): p. e83-e89.
16. Mihretie Gedfew, M.D., Belayneh Mengist, Hailie Amaha, Dessalegn Hailie and Bekalu Bewket, *Prevalence of HIV Sero-discordance among Couples in Sub Saharan Africa, 2019, Systematic Review and Meta Analysis.* International Journal of Women’s Health Care, 2020. **5**(1): p. 1-9.
17. Semiu Olatunde Gbadamosi, I.U.I., William Nii Ayitey Menson, John Olajide Olawepo, Tamara Bruno, Amaka Grace Ogidi, Dina V. Patel, John Okpanachi Oko, Chima Ariel Onoka, Echezona Edozie Ezeanolue, *Targeted HIV testing for male partners of HIVpositive pregnant women in a high prevalence setting in Nigeria.* PLoS ONE, 2019. **14**(1): p. 1-12.
18. Won, C.K.M.P.A.C.C.B.M.N.R.K.W.M.E.M.R.L.K.C.V., *Addressing the First 90: A Highly Effective Partner Notification Approach Reaches Previously Undiagnosed Sexual Partners in Tanzania.* AIDS and Behavior, 2017. **21**(8): p. 2551–2560.
19. Houlin Tang, Z.W., Yurong Mao, Javier Cepeda, Jamie Morano, *Risk Factor Associated with Negative Spouse HIV Seroconversion among Sero-Different Couples: A Nested Case-Control Retrospective Survey Study in 30 Counties in Rural China.* PLOS ONE, 2016. **11**(10): p. 1-18.
20. Kerebih Bantigen, L.K., Haweni Negeri, Mekonen Kebede, Addisu Wassie, Keralem Bishaw, Getaye Tesema, *Rate of HIV Seroconversion Among Seronegative Male Partners Living with HIV Positive Women.* HIV/AIDS - Research and Palliative Care, 2021. **13**: p. 125–134.

21. Sasisopin Kiertiburanakul, P.W., Angsana Phuphuakrat, Darunee Chotiprasitsakul, Somnuek Sungkanuparph, *Prevalence of HIV infection access to HIV care and response to antiretroviral therapy among partners of HIV infected individuals in Thailand*

PLoS ONE, 2018. **13**(6): p. 1-12.

22. Vu Van Tam, D.D.C., Tobias Alfven, Ho Dang Phuc, Nguyen Thi Kim Chuc, Nguyen Phuong Hoa, Vinod Diwan and Mattias Larsson, *HIV sero‐discordance among married HIV patients initiating anti‐retroviral therapy in northern Vietnam.* AIDS Research and Therapy, 2016. **13**(1): p. 1-7.

23. Nabukenya, A.M., Nambuusi, Aminah, Matovu, Joseph K.B, *Risk factors for HIV infection among married couples in Rakai, Uganda: A cross-sectional study.* BMC Infectious Diseases, 2020. **20**(1): p. 1-8.

Manuscript Click here to access/download;Manuscript;Manuscript R2.docx

Prevalence Of HIV Seropositive Status and Associated Factors Among Family Members Of Index Cases Of Antiretroviral Clinical Attendants in Sodo Town, Southern Ethiopia

Alemayehu Kefale[1], Kassa Daka[2], Amene Abebe[2], Dereje Haile[2], Kebreab Paulos[2], Abdulbasit Sherefa[3], Animut Addis[4] Muluken Gunta[1], Asamino Ayza[1]

[1]Wolaita Zone Health Department, Wolaita Sodo, Southern Ethiopia

[2]School of Public Health, Wolaita Sodo University, Sodo, Southern Ethiopia

[3]Deparment of Public Health, Worabe University, Worabe, Southern Ethiopia

[4]Department of Disease Prevention and Control Core Process, ICAP, Hawassa, Ethiopia

First author email: alemayehukefale@yahoo.com: AK

Corresponding author email: derehaile2010@gmail.com: DH

Abstract

Background:

Human immunodeficiency virus is primarily transmitted through sexual contact with an infected partner and babies born to mothers infected with the virus. Partners of people living with HIV and children whose parents have HIV are at higher risk of contracting HIV unless they take preventive measures. This study aimed at identifying prevalence and determinants of HIV infection among family members of index cases on antiretroviral treatment (ART)

Methods:

A community-based cross-sectional study was conducted among 623 randomly selected family members of HIV index cases in Sodo Town from February to June 2021. A pre-tested structural questionnaire was used to collect data. Binary logistic regression was used to identify variables independently associated with the outcome variable. The adjusted odds ratio (AOR) with 95% confidence interval (CI) was used to show the strength of association, and a *P*-value 0.05 was used as a cut-off point to determine the level of statistical significance of point estimate..

Results:

This study revealed that 31.5% (95%CI: 27.6-35.2%)of family members of index cases were HIV seropositive. In subgroup analysis, this study also revealed that 11.1%95%CI (8.4–14.5%) of biological children and 69.6% 95%CI (63.1–75.6%) of spousal partners of index cases were HIV seropositive. Immediate ART initiation of index cases (AOR = 0.148, 95%CI: 0.067-0.325), being bedridden or ambulatory functional status at enrollment (AOR = 7.71, 95%CI: 3.5-17), and baseline CD4 level of 350 cells/ml (AOR = 8.06, 95%CI: 1.8-36) were statistically significant with the outcome variable among biological children. Among spousal partners, STI history or symptoms (AOR = 5.7, 95%CI: 1.86–17.5), early disclosure (AOR = 0.062, 95%CI: 0.024-0.159), immediate ART initiation (AOR = 0.172, 95%CI: 0.044–0.675), and duration of infection (AOR = 5.09, 95%CI: 1.8–14.4) were statistically associated with the outcome variable.

Conclusion:

As evidenced by our data, the risk of HIV among family members of index cases is high. Interventions like immediate ART initiation, early disclosure, screening, and early treatment of STIs for minimizing HIV transmission might be given.

Keywords: HIV infection, HIV seropositive, biological children, spousal partners, index case,.

Introduction

Index testing is a voluntary process where counsellors or health care providers ask a newly diagnosed HIV positive patient or an HIV positive patient already accessing HIV treatment to list all partners who could have been exposed to HIV through them, the index cases.

[1].

ICT has two models of testing(1)Facility Based ICT that a counselor talks with the index cases on ART at the health facility,and gets the names and contact information for sexual partner(s) and biological children under 15 years old in Antiretroviral Treatment (ART) service giving facility and (2) community-based ICT: a counsellor traces sexual partner(s) of index cases and their biological children who are under 15 years of age through home-to-home visits, offers HTS, and links newly identified People Living with HIV (PLHIV) to ART services [1]

According to the United Nations Program on HIV/AIDS (UNAIDS), 37.7 million people worldwide were living with HIV/AIDS in 2021, including 1.7 million children under the age of 15 years. [2]. In Eastern and Southern Africa (ESA), 20.6 million PLHIV were living in 2021, and of which 670,000 people were newly infected by HIV [2]. Despite this burden, a transformative and remarkable progress has been made in the past decades with regards to the expansion of ART indicating that from of the total number of PLHIVs in ESA in 2021, 16 million were on treatment [2].In 2021, in Ethiopia, there were an estimated 620,000 PLHIV, of which 44,000 (7.1%) were children under 15 years of age and 360,000 (62%) were females [3].

There were 12,000 new annual infections, among which 2,800 were children under 15 years of age. There were 13,000 annual deaths (11,000) adults and 2,000 children under 15 years of age. Of the total PLHIV in Ethiopia, 483,127 (465,457 adults and 17,670 children under 15 years of age) were receiving ART[3]. According to the Ethiopian Demographic and Health Survey (EDHS) 2016, the national HIV prevalence in adults aged 15–49 years was 0.9 % (0.7 % in males and 1.2% in females)[4].

'HIV/AIDS in Ethiopia is regularly categorized as “generalized“ among the adult population, , with a significant variation in the burden of HIV across a geographic area and population subgroups[5]. The urban HIV prevalence is seven times higher than the rural HIV prevalence. Among the regional states, the HIV prevalence ranges from 4.8% in Gambella to below 0.1 in Ethiopian Somali region [6]. There is also a disproportionally high HIV burden among population groups such as sexual partners of PLHIV, female commercial sex workers, long truck drivers, prisoners, widowed and divorced urban women, and mobile workers, where the HIV prevalence rate is 28%, 23%, 4.9%, 4.2%, 3.5%, and 1.5%, respectively[4, 7].

.

Ethiopia has changed its universal HIV testing strategy, which is testing all individuals in the population who were at risk to yield-based target test and treat strategy. Family members of index cases as a target population for this strategy [8].For the implementation, the country has adopted the World Health Organization (WHO) Partner and Family-based Index Case Testing (PFBICT) or ICT strategy; a voluntary process where counselors and/or healthcare workers ask index cases to list all sexual partners in the past year and under15-year-old biological children. This breaks the chain of transmission by offering HTS to people who are exposed to HIV to provide preventive services for those testing HIV-negative, and if positive, link them to cared and treatment services, a cascade that will contribute to attaining the 95-95-95 targets. Some index cases might not have yet disclosed their HIV status, exposing the family members to an ongoing risk of contracting HIV. Therefore, index cases' concerns should be addressed to improve disclosure and testing service uptake among sexual partners and under 15-years old biological children [1].In Ethiopia, the majority of HIV infections among spousal partners are heterosexual, and 90% of HIV transmission among biological children under the age of 15 is through mother-to-child transmission (MTCT)[4, 9].

This indicates that HIV-negative spousal partners of index cases and biological children under 15 years of age whose parents have HIV or family members of index cases are at a higher risk of contracting HIV. To reduce the risk of HIV transmission, family members of index cases would need to know their HIV status. Testing for HIV and linking those who are positive to care and treatment services will improve the health outcomes of the seropositive family members [7].

Nevertheless a study conducted on the assessment of tracing family members of HIV- positive people for HIV testing and associated factors among ART clinic attendants at a referral hospital in Northwest Ethiopia revealed that many children and adults were traced and tested for HIV. Yet, there were many untested partners, children and other individuals living with index cases who were not tested[10].

However, no similar study was conducted to assess HIV seropositive status and associated factors among family members of index cases and ART clinic attendants in this town. This study aimed to assess the prevalence of seropositive status and associated factors among family members of index cases among ART clinic attendants in Sodo Town. 'HIV serostatus information on family members of index cases, which will contribute to improving HIV care and testament services and epidemic control in Ethiopia'.

Materials and methodsStudy Area and Period

The study was conducted at Sodo Town, which is located in Wolaita Zone, South Ethiopia. The Town, which is the capital city of the Zone, is located 380 km south of Addis Ababa, the capital city of Ethiopia, and 157 km south of Hawassa, the capital city of Southern Nations, Nationalities, and Peoples Region (SNNPR). Sodo Town is further divided into three sub-cities and 25 kebele (lowest administrative entity)”.

According to the 2021 population and housing census projection, Sodo Town has a total population of 244,817, of which 121,184 are males and 123,633 are females. There are two hospitals, three health centers, and 25 health posts. There are 3 ART service-delivery facilities in the town, of which two are hospitals and the remaining one is a health center. This study was conducted from February 18–June 30, 2021.

Study design

The study was a community-based cross-sectional study.

Source population

The source population of this study seems all patients coming to the health facilities involved in this study Study population

All family members aged 2–14 years old, biological children and spousal partners of index cases of ART clinic attendants in Sodo Town

Study participants

The study subjects seem all index cases coming at ART clinics at the study health facilities and their family members. Inclusion and Exclusion criteria

Inclusion criteria

An index case living in Sodo who at least had one biological child aged 2–14 years or a spousal partner.

Biological children 2–14 years old and an index case living in Sodo who at least had one biological child aged 2–14 years or a spousal partner.

Exclusion criteria

Index cases who didn’t disclose their status

Biological children (2–14 years old) and spousal partners who couldn’t respond due to serious illness or not willing to participate

Sample size, Sampling technique, and procedure

The ART clinics at all three health facilities in Sodo Town (Sodo Health, Sodo Teaching and Referral Hospital, and Sodo Christian General Hospital) were included as study sites. According to the report of each facility, the total HIV-positive patients currently on ART at the end of

September 2020 was 2,317 (Sodo HC = 662, Sodo Teaching and Referral Hospital = 1530, and Sodo Christian General Hospital = 125). And among these 2,213 were adults aged $\geq$18 years. As a first step from the PFBICT registration book, we identified all adult clients living in in Sodo Town, which were 1,144. By using HIV testing positive dates between each spousal partner, we then identified 834index cases. Of the total index case (834), only 723 who had at least one family member were selected and given a serial code. The unique ART code numbers given by the national program to the facilities were used to identify each study facility." In a study done at public health facilities in Butajira Town, Southern Ethiopia, HIV-positive status disclosure among HIV-positive adult patients attending ART clinics, the magnitude of non-disclosure was 10%[11].

Of 723 index patients, 72 were expected not to disclose and 651 were expected to disclose their status to their family members. Since all index cases were manageable, we included them all and selected them as total index cases. Finally, from each disclosed index case, only one family member was selected by lottery method, as a result, had a total of 651 family members from all ART sites.

Study variables

Dependent variable

HIV seropositive status among biological children of index cases

HIV seropositive status among spousal partners of index cases

Independent variables

Socio-demographic and economic variables: age, sex, residence, marital status, education, wealth status, and occupation.

Index case-related factors: duration of a relationship, ART initiation status, enrollment status, history of treatment failure, history of adherence, WHO stage, viral load (VL), CD4+ and disclosure status

Sexual and substance use behavior of spousal partners: condom use, substance use (alcohol drinking, chat chewing &tobacco smoking), number of lifetime sexual partnership or multiple sexual partners, and type of current marriage (polygamous/monogamous)

HIV testing and STIs history: ever tested or not, sexually transmitted infections.

Data collection instrument and procedure

The data collection tool was an interviewer-administrator structured questioner developed through the review of related literature. It contains information on socio-demographic and economic factors, sexual and substance use behaviors of spousal partners, HIV testing and STIs-related history factors, and index case-related factors.

Following disclosure of HIV status by the index cases, data collectors fixed appointments with them and their families for home visits At the start of the visit, the purpose of the study was explained to study participants and informed verbal consent obtained from spousal partners and consent for all biological children was obtained from the parents, and assent was obtained from children 12-14 years of age. In those who were HIV-positive, only interviews were adminstered by the investigators,, but if their status was unknown or negative, the participants were interviewed and tested for HIV. . Parents were interviewed on behalf of biological children. The testing procedure strictly followed the current national testing algorithm.. Unique ART numbers were used to link primary index case data with that of the family members and with their secondary data in patients' records for analysis.

Operational definitions

Index case (:) first diagnosed spousal partner with HIV infection in the family

Biological children(:)-children born from the index case and aged 2-14 years

Spousal partner(:)individuals who are currently married or engaged or in a steady relationship

Family members of index case(:)biological children and/or spousal partner

HIV seropositive status (:) being reactive as per the national HIV testing algorithm test

Treatment failure (:) clinically stage III or IV, CD4+ <200cells/ml or viral load >/1000 copies/ml after 6 months of ART treatment

Disclosure(:) Early, when HIV positive family members share their status to his family members within one month, and late, if not disclosed within a month or disclosed after a month of diagnosis

Condom use (:) always, if used at each sexual intercourse, sometimes, if not used at every sexual intercourse e.g. for only prevention of pregnancy and never, not used at all

ART initiation status (:) Immediate, if clients started ART whenever eligible, and late, if not started ART though eligible to start based on eligibility criteria.

Viral load status (:) suppressed, if number of copies /ml <1000, and unsuppressed, if number of copies /ml>/1000 after taking ART at least for 6.months in the last 12 months.

Data management and quality control

The English version of the questionnaire was translated into the Amharic language and checked for comprehension and consistency. Then it was translated back into English. The Amharic version was used for to collect data. The questionnaire was pre-tested by the data collectors and the supervisors in 5% of the sample at Boditi Health Center. The results were used to modify the questionnaire as appropriate.. Eight data collectors and two supervisors working in the ART clinic were recruited and trained. Two-day training introduced the objective of the study, familiarized data collectors with the questionnaire, the sampling procedure and interviewing techniques, and the HIV testing procedures. The supervisors and the principal investigator had a close follow-up of the day-to-day data collection process to ensure completeness and consistency of the questionnaire administered each day. The collected data were rechecked for its completeness and consistency by the supervisors and principal investigators before being transferred into computer software. A non-overlapping numerical code was given for each questionnaire.

Data analysis procedure

EPI data 4.2 statistical software was used for data entry, cleaning, and codingof the variables. The data were exported to SPSS version 25 for analysis. Descriptive statistics were used to summarize the data and check for the distribution of the study variables. A bivariate analysis was done by using a binary logistic regression. A*P*-value of 0.25 was used as a cut-off point to select candidate variables for multivariate logistic regression, the analysis used to determine the association between the independent and dependent variables having controlled for confounding effects. AOR with 95%CI was used to determine the level association. The Hosmer and Lemeshow's goodness of fit test was used to check for model fitness. A variance inflation factor (VIF)or tolerance test was done to check for correlation. A *P*-value of 0.05 was used as a cut-off pointto determine statistical significance and the results were presented in narratives and tables.”.

Ethical consideration

Ethical clearance was obtained from the Ethical Review Board of Wolaita Sodo University College of Health Science and Medicine. Formal permission letters for each health facility were written by the Zonal Health Department and Town Health Office. The purpose of the study was explained to the study participants. Both informed written and verbal consent were obtained from the parents. Moreover, assent was obtained from the children aged 12–14 years.

. The information obtained from the study participants was kept confidential. The HIV testing and interview were undertaken privately in a scheduled area. Family members who tested negative were counseled about further preventive measures, and those who were positives were linked to a nearby ART clinic where care and support were given according to the national guidelines.

RESULTS

Socio-demographic and economic characteristics of family members of index cases

A total of 623 family members of index cases in Sodo Town were Interviewed, which was a response rate of 94%.While the majority, 406 (65.2%), were biological children, nearly one-third 217(34.8%) were spousal partners. The mean (SD) age of biological children was 8.9 (± 3.66) and of the spousal partner was 36.9 (± 9.26) years. Of the respondents, nearly half (51.5%) were females, more than half (54.6%) were Orthodox Christians, and one-third (32.6%) of the respondents wealth status in the first quartile. Including 1072 biological children (2–14) years old (1.72 biological children per index case), a total of 2932 family members (4.71 family members per index case) were identified from 623 index cases (Table 1).

Table 1 Socio-demographic and economic characteristics of members of index cases in Sodo Town, Southern Ethiopia, 2021(N=623).

Variables	Category	Frequency	Percentage
Sex	Male	302	48.5
	Female	321	51.5
Age	<5	87	14.0
	5-9	126	20.2
	10-14	192	30.8
	15-29	43	6.9
	≥30	175	28.1
Religion	Orthodox	340	54.6
	Protestant	249	40.0
	Muslim	26	4.2
	Catholic	8	1.3
Wealth index	First quartiles	203	32.6
	Second quartile	211	33.9
	Third quartile	209	33.5

Family size	1-3<br>4-6<br>≥7	129<br>447<br>47	20.7<br>71.7<br>7.5
2-14 yrs. old biological child size	<3<br>≥3	506<br>117	81.2<br>18.8
Alive parents	Father<br>Mother<br>Both	26<br>123<br>474	4.2<br>19.7<br>76.1
Relation to the index	Spouse<br>Biological child	217<br>406	34.8<br>65.2
Even though we expected 651 index cases disclosures from 723 eligible index cases to determine sample size, 662 were disclosed during data collection and used as the denominator to determine non-response rate.			

Sexual and substance use behavior of spousal partners of index case

Of the 217 spousal partners, nearly one-fourth (23%) had 10 or more lifetime sexual partners. The majorities (84.8%) were in a monogamous relationship; 69.1% had been living together with their spouse for more than ten years; and (68.2%) had an age difference of ten years or more between spousal partners. Despite the fact that most (76.5%) had been educated on condom use, more than half (60.8%) had never used one during sexual intercourse with their spousal partners. While the majority (78.8%) had never smoked tobacco, 70% had chewed chat, 9.7% had drunk alcohol daily (Table 2).

Table 2 Sexual and substance use behavior of spousal partners of index cases in Sodo Town, Southern Ethiopia, 2021(N=217)

Variables	Category	Frequency	Percentage
Number of life time sexual partners	<5<br>5-9<br>≥10	89<br>78<br>50	41.0<br>35.9<br>23.0
How do you describe the type of your current	Polygamous	33	15.2

marriage	Monogamous	184	84.8
Is this your first marriage or partnership	Yes<br>No	28<br>189	12.9<br>87.1
Are you the first spouse for your spouse	Yes<br>No	17<br>200	7.8<br>92.2
Duration of spousal partners living together in year	<5<br>5-9<br>≥10	26<br>41<br>150	12.0<br>18.9<br>69.1
Age difference between spousal partners	<5<br>5-9<br>≥10	22<br>47<br>148	10.1<br>21.7<br>68.2
Ever discussed condom use with your spousal partner	Yes<br>No	166<br>51	76.5<br>23.5
Ever used a condom during sexual intercourse with your spousal partner	Yes<br>No	98<br>119	45.2<br>54.8
How regularly did you use condoms during sexual intercourse with spousal partner	Never<br>Sometimes<br>Always	132<br>72<br>13	60.8<br>33.2<br>6.0
Ever drunk alcohol	Never<br>Occasionally<br>Daily	42<br>154<br>21	19.4<br>71.0<br>9.7
Ever used Tobacco	Never<br>Occasionally<br>Daily	171<br>26<br>20	78.8<br>12.0<br>9.2
Ever chaw chat	Never<br>Occasionally	152<br>65	70.0<br>30.0

HIV testing history and serostatus of family members of index cases

Of the 267 family members, most (90.9%) had ever been tested for HIV, and 57 (9.1%) had never been tested. Of the ever-tested participants, 186 (32.9%) had positive status. Of those who

had never been tested or whose results were negative (437), 10 (2.8%) were newly identified positives. The overall prevalence among family members was 31.5% (196/623 cases). (Table 3)

Table 3: HIV serostatus and testing history-related factors of family members of index cases in Sodo Town, Southern Ethiopia, 2021

Variables	Category	Frequency	Percentage
Have you ever tested for HIV(N=623)	Yes<br>No	566<br>57	90.9<br>9.1
What was the result if you had been tested(N=566)	Positive<br>Negative	186<br>380	32.9<br>67.1
If never been tested or the result was negative, what is the current result? (practically confirmed)(N=437)	Positive<br>Negative	10<br>427	2.29<br>97.71
Final current HIV serostatus (N=623)	Positive<br>Negative	196<br>427	31.5<br>68.5

In a sub-group analysis of the biological children (N=406), 353 (86.9%) had ever been tested, and 53 (13.1%) have not been tested. Of the ever-tested participants, 37 (10.5%) had positive status. Of those who had never been tested or whose results were negative, eight (2.8%) were newly identified positives. Of all the biological child respondents, 45 (11.1%) were positive and 361 (88.9%) were negative (Table 4).

Table 4: HIV serostatus and testing history of biological children of index cases, Sodo Town, Southern Ethiopia, 2021 (N=406)

Variables	Category	Frequency	Percentage
Have you ever tested for HIV	Yes<br>No	353<br>53	86.9<br>13.1
What was the result if you had been tested(n=353)	Positive<br>Negative	37<br>316	10.5<br>89.5
If never been tested or the result was negative, what is the current result? (practically confirmed)(n=369)	Positive<br>Negative	8<br>361	2.17<br>97.83
Final current HIV serostatus	Positive<br>Negative	45<br>361	11.1<br>88.9

In a sub-group analysis of the spousal partners (N=217)nearly one-fourth (23%) had STI symptoms or a history of STI in the 12 months prior to data collection. All (100%) heard about HIV testing and counseling service provision and thought testing would be important to family members of PLHIVs. A majority (98.2%) of them were ever tested, and 149 (70%) had HIV positive status.. Of those who had never been tested or whose results were negative, two (3%) were newly identified HIV positive patients. Of the spousal partners respondents, 151 (69.6%) were HIV positive and 66 (30.4%) were negative (Table 5)

Table 5: Sexually transmitted Infections and HIV testing history of spousal partners of index cases in Sodo Town, Southern Ethiopia, 2021 (N=217)

Variables	Category	Frequency	Percentage
Had history or symptoms of STIs (N=217)	Yes<br>No	50<br>167	23.0<br>77.0
Heard about HIV testing and counseling service provision to family members PLHIVs(N=217)	Yes<br>No	217<br>0	100<br>0
Do you think testing is important to family members of PLHIVs (N=217)?	Yes<br>No	217<br>0	100<br>0
Have you ever tested for HIV(N=217)?	Yes<br>No	213<br>4	98.2<br>1.8
What was the result if you had been tested (N=213)?	Positive<br>Negative	149<br>64	70.0<br>30.0
If never been tested or the result was negative, what is the current result? (practically confirmed) (N=68)	Positive<br>Negative	2<br>66	2.94<br>97.06
Final current HIV serostatus (N=217)	Positive<br>Negative	151<br>66	69.6<br>30.4

Characteristics of index cases of family members

From a total of 623 index cases, 386 (62%) disclosed their status to at least one family member within a month of diagnosis, and the majority of 476 (76.4%) started ART immediately as they were eligible. Most, 97% and 79.9%, of the index cases had current and at enrollment working functional status, respectively. Five hundred fifty eight (89.6%) had a current suppressed viral load and 330 (53%) had a low CD4 count (350 cells/ml) at ART enrollment. The majority, 505

(81.1%) and 406 (65.2%)of the index cases had no history of treatment failure and poor adherence, respectively (Table 6).

Table 6**: Clinical and behavioral characteristics of HIV index cases in Sodo town, Southern Ethiopia, 2021 (N=623)**

Variables	Category	Frequency	Percentage
Notification of disclosure of HIV status	≥1month	237	38.0
	<1month	386	62.0
Immediate ART initiation	Yes	476	76.4
	No	147	23.6
Functional status at enrollment	Bedridden	40	6.4
	Ambulatory	85	13.6
	Working	498	79.9
Current functional status	Bedridden	5	0.8
	Ambulatory	14	2.2
	Working	604	97
WHO stage at enrollment	Stage One	265	42.5
	Stage Two	167	26.8
	stage Three	152	24.4
	stage Four	39	6.3
Current WHO stage	Stage One	593	95.2
	Stage Two	25	4.0
	Stage Three	0	0
	stage Four	5	0.8
Duration of infection since known	<60 month	164	26.3
	60-120 month	189	30.3
	≥120 month	270	43.3
Time to start ART since known infection	≤1 month	254	40.8
	2-11 month	235	37.7
	≥12 month	134	21.5
Base line CD4	<350 cells/ml	330	53.0

	≥350 cells/ml	293	47.0
Current Viral Load(VL) status	Unsuppressed<br>Suppressed	65<br>558	10.4<br>89.6
History of treatment failure	Yes<br>No	118<br>505	18.9<br>81.1
History of poor adherence	Yes<br>No	217<br>406	34.8<br>65.2

Factors associated with HIV seropositive status among biological children

The bivariate binary logistic regression found five variables that affected the HIV seropositive status of the family members of index cases of HIV. These were immediate ART initiation if eligible; functional status at enrollment; WHO stage at enrollment; duration of infection since known; and baseline CD4 of index cases of HIV(Table 7).

The multivariate analysis in binary logistic regression showed that three out of five variables were significantly associated with the outcome variable. Biological children whose eligible index cases started ART immediately were 85% less likely to be seropositive than those who didn't (AOR = 0.148; 95%CI: 0.067-0.325). Biological children whose index cases' functional status was bedridden or ambulatory at enrollment were 7.71 times more likely to be seropositive compared to those whose status was working (AOR = 7.71; 95%CI: 3.5-17). Biological children whose index case baseline CD4 level was 350 cells/ml were 8.06 times more likely to be seropositive compared to those whose CD4 level was > 350 cells/ml (AOR = 8.06; 95%CI: 1.8-36)(Table 7).

Table 7: Factors associated with seropositive status among biological children of index cases in Sodo Town, 2021 (N=406)

Variables	Category	Serostatus of biological children		95%CI	
		Positive	Negative	COR	AOR
Immediate ART initiation	Yes	13(3.2%)	300(73.9%)	0.083(0.041-0.167)	0.148(0.067-0.325)***
	No	32(7.9%)	61(15.0%)	1	1

Functional status at enrollment	Bedridden or Ambulatory	32(7.9%)	51(12.6%)	14.96(7.36-30.4)	7.71(3.5-17)***
	Working	13(3.2%)	310(76.4%)	1	1
WHO stage at enrollment	Stage1 or 2	10(2.5%)	276(68.0%)	1	1
	Stage3 or 4	35(8.6%)	85(20.9%)	11.7(5.4-23.9)	1.53(0.512-4.55)
Duration of infection since known	<60 month	3(0.7%)	95(23.4%)	1	1
	60-120 month	13(3.2%)	121(29.8%)	3.4(0.942-12.3)	0.597(0.119-2.99)
	≥120 month	29(7.1%)	145(35.7%)	6.33(1.88-21.4)	0.853(0.181-4.02)
Base line CD4	<350	43(10.6%)	167(41.1%)	24.98(5.96-104)	8.06(1.8-36)**
	≥350	2(0.5%)	194(47.8%)	1	1

*** Statistically significant (p=0.01–<0.05), ** Strong statistical significance (p=0.001–<0.01), *** Very strong statistical significance (p< 0.001)**

Factors associated with HIV seropositive status among spousal partners

The bivariate analysis showed sex, wealth index, ever used a condom, had a history or symptoms of STI in the 12 months before the survey among spousal partners and factors from the index cases such as; notification of disclosure status of HIV, ART initiation, if eligible, functional status at enrollment, WHO stage at enrollment and duration of infection since known were candidate variables for multivariable binary logistic regression analysis.

In multivariable logistic regression analysis, had a history or symptoms of STI ((AOR = 5.7; 95% CI: 1.86–17.5)), and from the index factors like notification of disclosure of HIV status (AOR=0.062; 95%CI: 0.024-0.159), ART initiation status(AOR = 0.172; 95%CI: 0.044–0.675), functional status at enrollment, WHO stage at enrollment(AOR = 7.27; 95%CI: 2.37–22.3, and duration of infection since known(AOR = 5.9; 95%CI: 1.8–14.4) were factors significantly associated with HIV seropositive status of spousal partners of index cases.

Spousal partners who had a history or symptoms of STI in the previous 12 months were 5.7 times more likely to be seropositive than their counterparts (AOR = 5.7; 95% CI: 1.86–17.5).Spousal partners whose index cases disclosed their status within a month of diagnosis were 94% less likely to be seropositive than those whose index cases stayed a month or more (AOR=0.062; CI: 0.024-0.159). Spousal partners whose index cases started ART immediately as eligible were 83% less likely to be seropositive than those whose index cases started immediately (AOR = 0.172; 95%CI: 0.044–0.675). Spousal partners whose index cases were WHO stage III or IV at enrollment were 7.27 times more likely to be seropositive than those whose index cases were WHO stage I or II (AOR = 7.27; 95%CI: 2.37–22.3). Spouses of index cases who had known their status for more than 10 years were 5.9 times more likely to be seropositive than those who had known their status for less than 5 years (AOR = 5.9; 95%CI: 1.8–14.4)(Table8).

Table 8: Factors associated with seropositive status among spousal partners in Sodo Town, 2021 (n=217)

Variables	Category	Serostatus of spousal partner		95%CI	
		Positive	Negative	COR	AOR
Sex	Male	76(35.0%)	42(19.4%)	1	1
	Female	75(34.6%)	24(11.1%)	1.73(0.953-3.13)	1.84(0.75-4.52)
Wealth index	Frist percentile	46(21.2%)	23(10.6%)	0.645(0.317-1.31)	0.551(0.193-1.57)
	Second percentile	43(19.8%)	23(10.6%)	0.603(0.295-1.23)	0.345(0.125-1.949)
	Third percentile	62(28.6%)	20(9.2%)	1	1
Ever used a condom	Yes	59(27.2%)	39(18.0%)	1	1
	No	92(42.4%)	27(12.4%)	2.25(1.25-4.06)	1.37(0.561-3.36)
Had or symptoms of an STI	Yes	41(18.9%)	9(4.1%)	2.36(1.07-5.2)	5.7(1.86-17.5)**

	No	110(50.7%)	57(26.3%)	1	1
Notification of Disclosure of index cases	≥1month	106(48.8%)	10(4.6%)	1	1
	<1month	45(20.7%)	56(25.8%)	0.076(0.036-0.162)	0.062(0.024-0.159)***
Immediate ART initiation of index cases	Yes	100(46.1%)	63(29.0%)	0.093(0.028-0.312)	0.172(0.044-0.675)*
	No	51(23.5%)	3(1.4%)	1	1
Functional status at enrollment of index cases	Bedridden or Ambulatory	40(18.4%)	2(0.9%)	11.5(2.7-49.3)	1.23(0.198-7.68)
	Working	111(51.2%)	64(29.5%)	1	1
WHO stage at enrollment of index cases	Stage1or 2	86(39.6%)	60(27.6%)	1	1
	Stage3 or 4	65(30.0%)	6(2.8%)	7.56(3.08-18.6)	7.27(2.37-22.3)**
Duration of infection since known of index cases	<60 month	31(14.3%)	35(16.1%)	1	1
	60-120 month	40(18.4%)	15(6.9%)	3(1.4-6.47)	3.04(0.994-9.32)
	≥120 month	80(36.9%)	16(7.4%)	5.65(2.74-11.6)	5.09(1.8-14.4)**

*** Statistically significant (p=0.01–<0.05), ** Strong statistical significance (p=0.001–<0.01), *** Very strong statistical significance (p < 0.001).**

DISCUSSION

In this study, the HIV seropositive status among family members was 31.5% (95%CI; 27.6-35.2%).. This implies that family members of the index cases are at high risk for HIV infection.. The prevalence is consistent with the findings of studies conducted at Felege Hiwot Referral Hospital, Northwest Ethiopia and Zimbabwe which was 30% [10] and 32.6%[12] respectively.

However, it is lower lower than the prevalence reported from South Africa of 38% [13] and higher than the prevalence reported from Malawi of 22% [14]. The possible explanations for this difference might be due to variation in socio-demographic and economic characteristics, prevention strategies, and implementation differences across the nations.

In a sub-group-analysis of the prevalence of seropositive status among family members of index cases only on biological children, the prevalence of seropositive status in our study area was found to be 11.1% with a 95%CI of (8.4–14.5%). This finding is consistent with a study done at FelegeHiwot Referral Hospital, Northwest Ethiopia, which found 10.7%[10]. But our finding is much higher compared with other studies, including the Lesotho study that showed a prevalence of 1.4%, Malawi at 4%, and Kenya at 7.4% [15-17]. Possible explanations for this difference might be implementation differences (time of implementation, service coverage) of the prevention of mother-to-child transmission (PMTCT) strategy and socio-demographic and economic differences between nations.

In a sub-group-analysis of the prevalence of seropositive status among family members of index cases only on spousal partners, in our study area, was found to be 69.6% with a 95%CI (63.1-75.6%).

This finding is almost similar to a study done at FelegeHiwot Referral Hospital, Northwest Ethiopia; which was 71.5%[10]. But, this finding is lower than in a study conducted in SSA 76.23%[18]. On the other side, this finding is higher than a study conducted in Ethiopia, EDHS, 2016 28%[4].The possible explanation for this wide gap with the national prevalence might be that participants of index cases were the only new index cases in EDHS, 2016. Likewise, this finding is also higher than the study conducted on 37.7% in Nigeria and 61.9% in Tanzania [19, 20]. The possible explanations for this difference might be ART service coverage, prevention, implementation, and monitoring strategy differences from one country to another country.

This finding revealed that biological children whose index cases started ART immediately, based on eligibility criteria, were 85% less likely to be seropositive than those whose index cases didn't start. This might be the fact that initiation of ART increases CD4 and inversely decreases the viral load level of index cases, eventually preventing transmission of HIV to their biological children[9].

This study showed that biological children whose index cases were bedridden or ambulatory functional status at enrollment were 7.7 times more likely to be seropositive than those whose status was working. This finding also revealed that biological children whose index case baseline CD4 level was 350 cells/ml were 8.1 times more likely to be seropositive than those whose CD4 level was > 350 cells/ml. There is no study report related to this in this specific age group and almost 90% of biological children acquire the HIV infection through vertical transmission. However, the importance of these factors is emphasized by the national PMTCT guideline of Ethiopia [9]. In index cases who do not start ART as per the eligibility criteria, their CD4 level declines. This leads to viral replication, increase in vulnerabilities to opportunistic infections, and deterioration in patients functional status, thereby increasing the possibility of vertical viral transmission of HIV. This study revealed that spousal partners who had a history or symptoms of an STI in the 12 months before the survey were 5.7 times more likely to be seropositive than

their counterparts. It is consistent with the study conducted in 30 provinces of China [21]. The possible explanation might be that since STI and HIV have a linear relationship, and STI may be characterized by genital sores or ulcers, inguinal swelling that is exposed to HIV transmission. This finding is supported by EDHS, 2016 [4].

This finding of this study shows that spousal partners whose index cases disclosed their status within a month of diagnosis were 94% less likely to be seropositive than those whose index cases who stayed for a month or more after diagnosis. This implies that disclosure of their HIV positive status to their spousal partners raises awareness about their risk and need for testing. Disclosure also has important health benefits. It increases social support, fosters closer relationship to others, increases testing uptake, creates good prevention opportunities like condom utilization, and other prevention methods, and improves treatment adherence and retention, which increases CD4 and suppresses viral load level that leads to halting of transmission. This finding is supported by the National Comprehensive HIV Care Guideline of Ethiopia, 2018[8].

This finding revealed that those spousal partners whose index cases started ART immediately as on meeting the eligibility criteria criteria were 83% less likely to be seropositive than those whose index partners didn’t start immediately. This finding is consistent with the findings of a study conducted in Addis Ababa, Ethiopia [22]. This might be because ART initiation based on the eligibility criteria could lead to increase in index case CD4 count and VL suppression that decreases HIV transmission to spousal partners This study showed that spousal partners whose index cases who were at WHO clinical stage III or IV at enrollment were 7.25 times more likely to be seropositive than spousal partners whose index cases who were WHO clinical stage I or II. This finding is with the study conducted in Thailand and North Vietnam[23, 24]. This could be due to the fact that index cases who were at WHO stage III or IV had severe opportunistic infections that exposed the index case to decreased their CD4 levels and increased the viral load levels resulting in high viral transmission to spousal partners.

This study also revealed that spousal partners whose index cases lived with the virus since they knew their status for more than 10 years were 5.09 times more likely to be seropositive than those who knew their status for less than 5 years. It is consistent with the study conducted in Thailand and Rakia, Uganda [23, 25].

This implies that as the duration of the relationship between spousal partners increases, the rate of HIV transmission also increases.

Strength and Limitation

Because the factors of biological children and spousal partners of index cases are different, being able to address both outcomes in one study was the strength of this study.Family members whose

index cases did not disclosed the[illegible]atus or had follow-up outside of Sodo Town were not included in the study. Obstetric and as well as modes of transmission other than MTCT were excluded as the study focused on biological factors

Conclusion

In this study, HIV among family members of index cases in general and specifically in both biological children and spousal partners in Sodo Town is high. Late ART initiation as per the eligibility criteria, bedridden or ambulatory functional status during enrollment, and baseline CD4 350 cells/ml of index cases were the major factors associated with HIV seropositive status. in the spousal partners with HIV history or symptoms of STI, and whereas among the index cases, late notification of disclosure status, late ART initiation as per the eligibility criteria, WHO stage III or IV at enrollment, and duration of HIV infection since known of > 10 years were factors for the seropositive. In the context of Sodo Town, comprehensive HIV prevention, care, and treatment services need be promoted and implemented among family members of the index cases to increase the achievements of program targets in HIV epidemic control.

ACKNOWLEDGMENT

We are grateful to the Wolaita Sodo University, College of Medicine and Health Sciences for providing ethical clearance for this study. We are also indebted to our data collectors, supervisors, and the study participants.

Funding: no funding was received.

Author's contribution

Conceptualization: Alemayehu Kefale

Data curation: Alemayehu Kefale, Dereje Haile, Amene Abebe, Kassa Daka

Formal analysis: Alemayehu Kefale, Dereje Haile, Amene Abebe, Abdulbasit Sherefa

Investigation: : Alemayehu Kefale, Dereje Haile, Amene Abebe, Abdulbasit Sherefa, Animut Adiss, Muluken Gunta, Kassa Daka

Methodolgy: Alemayehu Kefale, Dereje Haile, Amene Abebe, Abdulbasit Sherefa, Animut Adiss, Muluken Gunta, Kassa Daka

Project Administration: Alemayehu Kefale, Animut Adiss, Muluken Gunta

Resources: Alemayehu Kefale, Animut Adiss, Muluken Gunta

Software: Alemayehu Kefale, Dereje Haile, Amene Abebe, Abdulbasit Sherefa, Kassa Daka

Supervision: Alemayehu Kefale, Dereje Haile, Amene Abebe, Abdulbasit Sherefa, Animut Adiss, Muluken Gunta

Validation: Alemayehu Kefale, Dereje Haile, Amene Abebe

Visualization: Alemayehu Kefale, Dereje Haile, Amene Abebe

Writing original draft: Alemayehu Kefale, Dereje Haile

Writing – review & editing: Alemayehu Kefale, Dereje Haile, Amene Abebe, Abdulbasit Sherefa, Animut Adiss, Muluken Gunta

References

1. WHO, *Partner and Family-Based Index Case Testing: A Standard Operating Procedure (SOP)*

2015: p. 3-20.

2. UNAIDS, *FACT SHEET 2021 Global Hiv Statistics.* Ending the AIDS epidemic, 2021(June): p. 1-3.
3. info, U.a.A., *Country factsheets, Ethiopia* Unaids, 2021: p. 1-6.
4. Agency, C.S., *Ethiopian Demographic and Health Survey(EDHS), 2016, HIV Report*. 2018.
5. *Ethiopian Public Health Institute (EPHI). Ethiopia Population-based HIV Impact Assessment*

(EPHIA) 2017-2018: Final Report. Addis Ababa: EPHI; August 2020.

6. CSA, E., WHO, *Min-Ethiopian Demographic Health Survey 2019.* 2019.
7. HAPCO, *HIV Prevention in Ethiopia National Road Map 2018 - 2020.* 2018. **https:// e**(November 2018): p. 25-54.
8. FMOH, E., *National Consolidated Guidelines for Comprehensive Hiv Prevention , Care and treatment.* 2018: p. 1-238.
9. MOH, *National Comprehensive and Integrated Prevention of Mother-Child Transmission of HIV Guideline* 2018. **6**(1).
10. Belayneh, T.W., *Assessment of Tracing Family Members of HIV Positive People for HIV Testing and Associated Factors among ART Clinic Attendants at Felege Hiwot Referral Hospital, Northwest Ethiopia.* Journal of Pharmacy and Alternative Medicine, 2017. **15**: p. 16-28.
11. Tamirat Melis Berhe, L.L., Addissu Alemayehu, Desalegn Ajema, Mustefa Glagn, Samuel Dessu, *HIV-Positive Status Disclosure and Associated Factors among HIV-Positive Adult Patients Attending Art Clinics at Public Health Facilities of Butajira Town Southern Ethiopia.* AIDS Research and Treatment, 2020. **2020**.
12. Nyikadzino Mahachi, A.M., Taurayi A Tafuma, Peter Mawora, Liz Kariuki, Bazghina-werq Semo,Moses H Bateganya, Tendai Nyagura, Getrude Ncube, Mike B Merrigan, Otto N Chabikuli and Mulamuli Mpofu, *Sustained high HIV case-finding through index testing and partner notification services: experiences from three provinces in Zimbabwe.* the International AIDS Society, 2019. **22**(S3): p. 23-30.

13. Dvora Joseph Davey, K.M.W., Claire Serrao, Marlien Prins, Madaline Feinberg, Ntokozo Mtonjana, Khanyo Hlophe, Lindiwe Zuma, Senate Sejake, and Todd Malone, *HIV Positivity and Referral to Treatment Following Testing of Partners and Children of PLHIV Index Patients in Public Sector Facilities in South Africa.* Journal of Acquired Immune Deficiency Syndromes, 2019. **81**(4): p. 365-370.
14. MALAWI: Improvement, Q.S., F O R, *INDEX CASE TESTING: A PROMISING STRATEGY FOR ACHIEVING HIV EPIDEMIC CONTROL.* Management Sciences for Health, 2018(June): p. 7-10.
15. Makhahliso Jubilee, F.J.P., Knowledge Chipango, Kenoakae Pule,Albert Machinda, Noah Taruberekera, *HIV index testing to improve HIV positivity rate and linkage to care and treatment of sexual partners, adolescents and children of PLHIV in Lesotho.* PLoS ONE, 2019. **14**(3): p. 1-12.
16. Saeed Ahmed, R.A.S., Katie Simon, Nora E. Rosenberg, Elijah Kavuta, Mwelura Harawa, Spencer Dick, Frank Linzie, Peter N. Kazembe and Maria H. Kim, *Index case-finding facilitates identification and linkage to care of children and young adults living with HIV/AIDS in Malawi.* Tropical Medicine and International Health, 2019. **22**(8): p. 1021-1029.
17. Anjuli D WAGNER, C.M.W., Irene N NJUGUNA, Elizabeth MALECHE-OBIMBO, Kenneth SHERR, Irene W INWANI, James P HUGHES, Dalton C WAMALWA, Grace C JOHN-STEWART, and Jennifer A SLYKER, *Active referral of children of HIV-positive adults reveals high prevalence of undiagnosed HIV.* Journal of Acquired Immune Deficiency Syndromes, 2016. **73**(5): p. e83-e89.
18. Mihretie Gedfew, M.D., Belayneh Mengist, Hailie Amaha, Dessalegn Hailie and Bekalu Bewket, *Prevalence of HIV Sero-discordance among Couples in Sub Saharan Africa, 2019, Systematic Review and Meta Analysis.* International Journal of Women's Health Care, 2020. **5**(1): p. 1-9.
19. Semiu Olatunde Gbadamosi, I.U.I., William Nii Ayitey Menson, John Olajide Olawepo, Tamara Bruno, Amaka Grace Ogidi, Dina V. Patel, John Okpanachi Oko, Chima Ariel Onoka, Echezona Edozie Ezeanolue, *Targeted HIV testing for male partners of HIVpositive pregnant women in a high prevalence setting in Nigeria.* PLoS ONE, 2019. **14**(1): p. 1-12.
20. Won, C.K.M.P.A.C.C.B.M.N.R.K.W.M.E.M.R.L.K.C.V., *Addressing the First 90: A Highly Effective Partner Notification Approach Reaches Previously Undiagnosed Sexual Partners in Tanzania.* AIDS and Behavior, 2017. **21**(8): p. 2551–2560.
21. Houlin Tang, Z.W., Yurong Mao, Javier Cepeda, Jamie Morano, *Risk Factor Associated with Negative Spouse HIV Seroconversion among Sero-Different Couples: A Nested Case-Control Retrospective Survey Study in 30 Counties in Rural China.* PLOS ONE, 2016. **11**(10): p. 1-18.
22. Kerebih Bantigen, L.K., Haweni Negeri, Mekonen Kebede, Addisu Wassie, Keralem Bishaw, Getaye Tesema, *Rate of HIV Seroconversion Among Seronegative Male Partners Living with HIV Positive Women.* HIV/AIDS - Research and Palliative Care, 2021. **13**: p. 125–134.
23. Sasisopin Kiertiburanakul, P.W., Angsana Phuphuakrat, Darunee Chotiprasitsakul, Somnuek Sungkanuparph, *Prevalence of HIV infection access to HIV care and response to antiretroviral therapy among partners of HIV infected individuals in Thailand*

PLoS ONE, 2018. **13**(6): p. 1-12.

24. Vu Van Tam, D.D.C., Tobias Alfven, Ho Dang Phuc, Nguyen Thi Kim Chuc, Nguyen Phuong Hoa, Vinod Diwan and Mattias Larsson, *HIV sero‐discordance among married HIV patients initiating anti‐retroviral therapy in northern Vietnam.* AIDS Research and Therapy, 2016. **13**(1): p. 1-7.
25. Nabukenya, A.M., Nambuusi, Aminah, Matovu, Joseph K.B, *Risk factors for HIV infection among married couples in Rakai, Uganda: A cross-sectional study*. BMC Infectious Diseases, 2020. **20**(1): p. 1-8.

Click here to access/download
Supporting Information
SPSS data 2021 PCA.sav

Click here to access/download
Supporting Information
Revised manuscript with track change.docx

Prevalence ~~REVALENCE~~ Of~~F~~ HIV Seropositive ~~EROPOSITIVE~~ Status ~~TATUS~~ and ~~AND~~ Associated ~~SSOCIATED~~ Factors~~ACTORS~~ Among ~~MONG~~ Family ~~AMILY~~ Members ~~EMBERS~~ Of~~F~~ Index~~NDEX~~ Cases~~ASES~~ Of~~F~~ Antiretroviral~~RT~~ Clinical ~~LINIC~~ Attendants in Sodo Town, Southern Ethiopia ~~TTENDANTSINSODO TOWN, SOUTHERN ETHIOPIA~~

Alemayehu Kefale[1], Kassa Daka[2], Amene Abebe ~~-~~[2], Dereje Haile[2], Kebreab Paulos[2], Abdulbasit Sherefa[3], Animut Addis[4]~~,~~ Muluken Gunta[1], Asamino Ayza[1]

[1]Wolaita Zone Health Department, Wolaita Sodo, Southern Ethiopia

[2]School of Public Health, Wolaita Sodo University, Sodo, Southern Ethiopia

[3]Deparment of Public Health, Worabe University, Worabe, Southern Ethiopia

~~[4]~~4Department of Disease Prevention and Control Core Process, ICAP, Hawassa, Ethiopia

Formatted: Not Superscript/ Subscript

First author email: alemayehukefale@yahoo.com: AK

Corresponding author email: derehaile2010@gmail.com: DH

Abstract

Background:

Human immunodeficiency virus is primarily transmitted through sexual contact with an infected partner and babies born to mothers infected with the virus~~heterosexual partners and vertical transmission to biological children~~. Partners of people living with HIV and children whose parents have HIV are at higher risk of contracting HIV unless they take preventive measures. This study aimed at identifying prevalence and determinants of HIV infection among family members of index cases on antiretroviral treatment (ART)~~This study assessed the prevalence of HIV seropositive status and associated factors among family members of index cases of ART clinic attendants in Sodo Town, Southern Ethiopia.~~

Methods:

A community-based cross-sectional study was conducted among 623 randomly selected family members of HIV index cases in Sodo Town from February to June 2021. A pre-tested structural questionnaire was used to collect data. Binary logistic regression was used to ~~investigate~~ identify variables independently associated with the outcome variable. The adjusted odds ratio (AOR) with 95% confidence interval (CI) was used to show the strength of association, and a *P*-value 0.05 was used as a cut-off point to determine the level of statistical significance of point estimate.~~used to declare the cut-off point in determining the level of significance~~.

Formatted: Font: (Default) Times New Roman, 12 pt, Italic

Results:

This study revealed that 31.5% (95%CI: 27.6-35.2%)~~revealed that 31.5% with 95%CI (27.6-35.2%)~~ of family members of index cases were HIV seropositive. In subgroup analysis, this study also revealed that 11.1%~~ with~~ 95%CI (8.4–14.5%) of biological children and 69.6% ~~with~~ 95%CI (63.1–75.6%) of spousal partners of index cases were HIV seropositive. Immediate ART initiation of index cases (AOR = 0.148, 95%CI: 0.067-0.325), being bedridden or ambulatory functional status at enrollment (AOR = 7.71, 95%CI: 3.5-17), and baseline CD4 level of 350 cells/ml (AOR = 8.06, 95%CI: 1.8-36) were statistically significant with the outcome variable among biological children. Among spousal partners, STI history or symptoms (AOR = 5.7, 95%CI: 1.86–17.5), early disclosure (AOR = 0.062, 95%CI: 0.024-0.159), immediate ART initiation (AOR = 0.172, 95%CI: 0.044–0.675), and duration of infection ~~since known~~ (AOR = 5.09, 95%CI: 1.8–14.4) were statistically ~~significant~~ associated with the outcome variable.

Conclusion:

As evidenced by our data, the risk of HIV among family members of index cases is high. Interventions like immediate ART initiation, early disclosure, screening, and early treatment of STIs for minimizing HIV transmission should might be given.

Keywords: HIV infection, HIV seropositive, biological children, spousal partners, index case,Seropositive status, HIV among biological children, HIV among spousal partners.

Introduction

Index testing is a voluntary process where counsellors or health care providers ask a newly diagnosed HIV positive patient or an HIV positive patient already accessing HIV treatment to list all partners who could have been exposed to HIV through them, the index casesIndex case testing (ICT) refers to HIV testing and counseling (HTC) service providers giving counseling for people diagnosed with the Human Immunodeficiency Virus (HIV) and offering HTC to their biological children under 15 years of age and sexual partners.

[1].

Index case testingICT has two models of testing:(1)Facility Based ICT that a counselor talks with the index cases on ART at the health facility,index case and gets the names and contact information for sexual partner(s) and biological children under 15 years old in Antiretroviral Treatment (ART) service giving facility and (2) community-based ICT: a counsellor traces sexual partner(s) of index cases and their biological children who are under 15 years of age through home-to-home visits, offers HTS, and links newly identified People Living with HIV (PLHIV) to ART services Community Based ICT: a counselors trace sexual partner(s) and biological children under 15 years old in the community through home to home visit of index cases house, offer HTS, and links new People Living with Human Immunodeficiency Virus (PLHIV) to ART[1]

According to the United Nations Program on HIV/AIDS (UNAIDS), 37.7 million people worldwide were living with HIV/AIDS in 2021, including 1.7 million children under the age of 15 years.

[2]. In Eastern and Southern Africa (ESA), 20.6 million PLHIV were living in 2021, ed and of which 670,000 people were newly infected by HIV [2]. Despite this burden, a transformative and remarkable progress has been made in the past decades with regards to the expansion of ART indicating that from a total ofof the total number of PLHIVs in ESA in 2021, sixteen million (16 million) were on treatment [2].

In 2021, in Ethiopia, there were an estimated 620,000 PLHIV, among of which 44,000 (7.1%) were children under 15 years of age and 360,000 (62%) were females [3].

There were ~~also~~ 12,000 new annual infections, among which 2,800 were children under 15 years of age., ~~with~~ There were 13,000 annual deaths (11,000) adults and 2,000 children under 15 years ~~old~~of age. Of the total PLHIV~~s~~ in Ethiopia, 483,127 (465,457 adults and 17,670 children under 15 years of age) were receiving ART[3].

According to the Ethiopian Demographic and Health Survey (EDHS) 2016, the national HIV prevalence in adults aged 15–49 years was 0.9 ~~percent~~ % (0.7 ~~percent~~ % in males and 1.2% in females)[4].

'HIV/AIDS in Ethiopia is regularly categorized as “generalized“ among the adult population, ~~With this general prevalence, the current epidemiology of HIV is heterogeneous~~, with a significant variation in the burden of HIV across a geographic~~al~~ area and population subgroups[5]. The urban HIV prevalence is seven times higher than the rural HIV prevalence. Among the regional states, the HIV prevalence ranges from 4.8% in Gambella to below 0.1 in Ethiopian Somali region ~~a~~[6]. There is also a disproportionally high HIV burden among population groups such as sexual partners of PLHIV, female commercial sex workers, long truck drivers, prisoners, widowed and divorced urban women, and mobile workers, where the HIV prevalence rate is 28%, 23%, 4.9%, 4.2%, 3.5%, and 1.5%, respectively[4, 7].

~~The epidemic in Ethiopia is concentrated in key populations and priority populations. Female sex workers (FSW) are important populations in Ethiopia.Priority populations are widowed, separated or divorced women; truck drivers; family members of index cases; and people who work in hot spot areas, both mobile and resident~~[7].

Ethiopia has changed its universal HIV testing strategy, which is testing all individuals in the population who were at risk ~~testing all individuals as a mass~~ to yield-based target ~~testing~~ test and treat strategy. Family members of index cases as a target population for this strategy ~~index case clients are one of these areas~~ [8].

For the implementation, the country has adopted the World Health Organization (WHO) Partner and Family-~~b~~Based Index Case Testing (PFBICT) or ~~Index Case Testing (~~ICT~~)~~ strategy; a voluntary process where counselors and/or healthcare workers ask index cases to list all sexual partners in the past year and under-15-year-~~-~~old biological children. ~~to~~ This breaks the chain of transmission by offering HTS to people who ~~have been~~are exposed to HIV to ~~give~~ provide preventive ~~services~~ services for those testing HIV-negative, ~~for negative~~ and if positive, link them to cared and treatment services, a cascade that will contribute to attaining the ~~, if positive, linkage and treatment that increase case detection and attain~~ 95-95-95 targets. Some index cases might not have yet disclosed their HIV status, exposing the family members to an ongoing risk of contracting HIV~~while family members have an ongoing risk of contracting HIV~~. ~~So~~Therefore, index cases' concerns should be addressed to improve disclosure and testing service uptake among sexual partners and under 15-years old biological children ~~under 15 years of biological children~~[1].

In Ethiopia, the majority of HIV infections among spousal partners are heterosexual, and 90% of HIV transmission among biological children under the age of 15 is through mother-to-child transmission (MTCT)[4, 9].

This indicates that HIV-negative spousal partners of index cases and biological children under 15 years of age whose parents have HIV or family members of index cases are at a higher risk of contracting HIV. To reduce the risk of HIV transmission, family members of index cases would need to know their HIV status. Testing for HIV and linking those who are positive to care and treatment services will improve the health outcomes of the seropositive family members ~~Testing for HIV and linking to care for those identified by the families of the index cases will improve the health outcomes of the seropositive family members~~[7].

Nevertheless~~But~~ a study conducted on the assessment of tracing family members of HIV-positive people for HIV testing and associated factors among ~~ART clinic attendants~~ ART clinic attendants at a referral hospital in Northwest Ethiopia revealed that many children and adults were traced and tested for HIV. Yet, there were many untested partners, children and other individuals living with index cases who were not tested~~at FelegeHiwot Referral Hospital, Northwest Ethiopia, reveals that 25.8% of index cases were not tested. Even though there were a significant number of family members not tested in this study, 30% of family members were seropositive~~[10].

~~Therefore, family members need to be tested, and those who become HIV positive should be linked to care and support services and start treatment. Those who become negative should get appropriate counseling on risk reduction.~~

However, no similar study was conducted ~~no study was conducted~~ to assess HIV seropositive status and associated factors among family members of index cases and ART clinic attendants in this town. This study aimed to assess the prevalence of seropositive status and associated factors among family members of index cases among ART clinic attendants in Sodo Town. 'HIV serostatus information on family members of index cases, which will contribute to improving HIV care and testament services and epidemic control in Ethiopia'~~The results of this study would emphasize the importance of obtaining the family members' information in routine clinical practice and could facilitate ending the HIV/AIDS epidemic vision in 2030~~.

Materials and methods~~METHODS AND MATERIALS~~

-Study Area and Period

The study was conducted at Sodo Town, which is located in Wolaita Zone, South Ethiopia. The Town, which is the capital city of the Zone, is located 380 km south of Addis Ababa, the capital city of Ethiopia, and 157 km south of Hawassa, the capital city of Southern Nations, Nationalities, and Peoples Region (SNNPR).~~SNNPR~~. Sodo Town ~~has been~~is further divided into three~~3~~ sub-cities and 25 kebele (lowest administrative entity)"~~kebele~~.

According to the 2021 population and housing census projection, Sodo Town has a total population of 244,817, of which 121,184 are males and 123,633 are females. There are two hospitals, three health centers, and 25 health posts. There are 3 ART service-delivery facilities

service-giving facilities in the town, of which two2 are hospitals and the remaining one1 is a health center. This study was conducted from February 18–June 30, 2021.

Study design

The study was a community-based cross-sectional studyA community-based cross-sectional study was conducted.

PopulationSource population

The source population of this study seems all patients coming to the health facilities involved in this study All family members aged 2–14 years old, biological children and spousal partners of index cases of ART clinic attendants in Sodo Town were considered as the source population.

Study population

All family members aged 2–14 years old, biological children and spousal partners of index cases of ART clinic attendants in Sodo Town

Study participants

The study subjects seem "All index cases coming at ART clinics at the study health facilities and their family members.". Selected family members, biological children aged 2–14, and spousal partners of index cases of ART clinic attendants in Sodo Town

Inclusion and Exclusion criteria

Inclusion criteria

An index case living in Sodo who at least had one biological child aged 2–14 years or a spousal partnerIndex cases who had at least one family member aged 2–14 years old were biological children or spousal partners.

Biological children 2–14 years old and an index case living in Sodo who at least had one biological child aged 2–14 years or a spousal partnerspousal partners of index cases who lived in Sodo Town were included.

Exclusion criteria

Index cases who didn't disclose their status

Biological children (2–14 years old) and spousal partners who couldn't respond due to serious illness or not willing to participate.

Sample size, Sampling technique, and procedure

The ART clinics at all three health facilities in Sodo Town (Sodo Health, Sodo Teaching and Referral Hospital, and Sodo Christian General Hospital) were included as study sites. All of the three (Sodo Health Center, Sodo Teaching and Referral Hospital, and Sodo Christian General Hospital) ART services giving health facilities in the town were included. According to the report of each facility, the total HIV--positive clients patients currently on ART (COA) at the end of September 2020 was 2,317 (Sodo HC = 662, Sodo Teaching and Referral Hospital = 1530, and Sodo Christian General Hospital = 125). Aand among these 2,213 were adults aged ≥ 18 years old. Primarily As a first step from the PFBICT Partner and Family-Based Index Case testing registration book, we identified all adult clients only living in in Sodo Town, which were Sodo Town, and there were 1,144. By using HIV Secondly, by using HIV testing positive dates between each spousal partner, we then identified 834we identified 834 index cases. Of the total Then, from the total index case (834), only 723 who had at least one family member were selected and given a serial code. The unique ART code numbers given by the national program to the facilities were used to identify each study facility.”The ART code numbers of facilities were used to identify each facility. In a study done at public health facilities in Butajira Town, Southern Ethiopia, HIV-positive status disclosure among HIV-positive adult patients attending ART clinics, the magnitude of non-disclosure was 10%[11].

Of 723 index patientsBy considering this from 723 index clients, 72 were expected to notnot to disclose and 651 were expected to disclose their status to their family members. Since all index cases were manageable, we included them all and and selected them as total index casesselected them as total index cases. Finally, from each disclosed index case, only one family member was selected by lottery method, as a result, had a total for a total of 651 family members from all ART sites.

Study variables

Dependent variable

HIV seropositive status among biological children of index cases

HIV seropositive status among spousal partners of index cases

Independent variables

Socio-demographic and economic variables: - age, sex, residence, marital status, education, wealth status, and occupation.

Index case-related factors: ~~-~~ duration of a relationship, ART initiation status, enrollment status, history of treatment failure, history of adherence, WHO stage, viral load (VL), CD4+ and disclosure status

Sexual and substance use behavior of spousal partners: ~~-~~ condom use, substance use (alcohol drinking, chat chewing &tobacco smoking), number of lifetime sexual partnership or multiple sexual partners, and type of current marriage (polygamous/monogamous)

HIV testing and STIs history: ~~-~~ ever tested or not, sexually transmitted infections.

Data collection instrument and procedure

The data collection tool was an interviewer-administrator structured questioner developed through the review of related literature. It contains information on socio-demographic and economic factors, sexual and substance use behaviors of spousal partners, HIV testing and STIs-related history factors, and index case-related factors.

Following disclosure of HIV status by the index cases, data collectors fixed appointments with them and their families for home visits ~~After addressing the disclosure issue, data collectors were appointed to index cases with their family members for visiting home.~~ At the start of the visit~~Then, during visiting~~, the purpose of the study was explained to study participants and informed verbal consent obtained from spousal partners ~~consent for spousal partners~~ and consent for all biological children was obtained from the parents, and assent was obtained from children 12-14 years of age. ~~and assent for biological children was taken.~~ In those who were HIV-positive, only interviews were adminstered by the investigators,~~Positive serostatus participants were only interviewed~~, but if their status was unknown or negative, the participants were interviewed and tested for HIV. ~~testing and interview were undergone simultaneously~~. Parents ~~or guardians~~ were interviewed on behalf of biological children. The testing procedure strictly followed the current national testing algorithm.~~The testing procedure was strictly followed by the current national testing algorithm~~. Unique ART numbers were used to link primary index case data with that of the family members and with their secondary data in patients' records for analysis. ~~For the data of the index case, unique ART numbers were used to review secondary data and link it with the primary data of their family members.~~

Operational definitions

Index case~~:~~ (:)- first diagnosed spousal partner with HIV infection in the family

Biological children(:)~~;~~-children born from the index case and aged 2-14 years ~~old~~

Spousal partner(:)~~;~~ individuals who are currently married or engaged or in a steady relationship

Family members of index case(:)~~;~~ biological children and/or spousal partner

HIV seropositive status(:)~~: —~~ being reactive as per the national HIV testing ~~algorithmic~~ algorithm test

Treatment failure(:)~~;~~ clinically stage III or IV, CD4+ <200cells/ml or viral load >/1000 copies/ml after 6 months of ART treatment

Disclosure(:)~~;~~ Early,~~=~~when HIV positive family members share ~~his~~ their status to his family members within one month, and late,~~=~~if not disclosed within a month or disclosed after a month of diagnosis

Condom use(:)~~;~~ always,~~=~~if used at each sexual intercourse, sometimes,~~=~~if not used at every sexual intercourse e.g. for only prevention of pregnancy and~~&~~ never,~~=~~not used at all

ART initiation status(:)~~; =~~Immediate, if clients started ART whenever eligible, and late, = if not started ART though eligible to start based on eligibility criteria.

Viral load status(:)~~;~~ suppressed,~~=~~ if number of copies /ml <1000, and unsuppressed,~~=~~ if number of copies /ml>/1000 after taking ART at least for 6.months in the last 12 months.

Data management and quality control

The English version of the questionnaire was translated ~~The English version was translated~~ into~~to~~ the Amharic language and checked for comprehension and consistency. Then it was translated back into English. The Amharic version was used for ~~gathering information~~to collect data. The questionnaire was pre-tested by the data collectors and the supervisors in 5% of the sample at Boditi Health Center~~Five percent of the questioners were pre-tested in the Boditi health center, which had similarities with the study population~~. The results were used to modify the questionnaire as appropriate.~~Then it was modified or contextualized~~. Eight data collectors and two supervisors working in the ART clinic were recruited and trained. Two-day training introduced the objective of the study, familiarized data collectors with the questionnaire, the sampling procedure and interviewing techniques, and the HIV testing procedures~~The two-day training addressed the objective of the study, the introduction of the questionnaire, sampling procedure, HIV testing procedure, and interviewing~~. ~~The data collectors and supervisors participated in pre-testing of the questionnaire for its understanding, which was not included in the study, and the result was used to correct unclear ideas and statements.~~ The supervisors ~~During data collection, the supervisors~~ and the principal investigator had a close follow-up of the day-to-day data collection process to ensure completeness and consistency of the questionnaire administered each day. The collected data were ~~After data collection, the collected information~~

was rechecked for its completeness and consistency by the supervisors and principal investigators before being transferred into computer software. A non-overlapping numerical code was given for each questionnaire.

Data analysis procedure

EPI data 4.2 statistical software was used for data entry, cleaning, and codingof the variables. The data were exported to SPSS version 25 for analysis. Descriptive statistics were used to summarize the data and check for the distribution of the study variables.A bivariate analysis was done by using a binary logistic regression. A*P*-value of 0.25 was used as a cut-off point to select candidate variables for multivariate logistics regression, the analysis used to determine the association between the independent and dependent variables having controlled for confounding effects. AORwith 95%CI was used to determine the level association. The Hosmer and Lemeshow's goodness of fit test was used to check for model fitness. A variance inflation factor (VIF)or tolerance test was done to check for correlation. A *P*-value of 0.05 was used as a cut-off pointto determine statistical significance and the results were presented in narratives and tables."EPI data 4.2 statistical software was used for editing, coding, and data entry, and then exported to SPSS version 25 for cleaning and analysis. A descriptive summary to see the distribution of the study participants with variables under the study was done. Bivariate analysis between dependent and independent variables was performed by using a binary logistic regression model; a P-value of 0.25 was used to select candidate variables for multivariate logistics regression. A VIF or tolerance test was done to check the correlation. Multivariate logistic regression analysis was done to adjust for possible confounding variables. A P-value of 0.05 was used as a cut-off point. An Adjusted Odds Ratio (AOR) with a 95% confidence interval (CI) was used to determine the level of association. The Hosmer and Lemeshow's goodness of fit test was used to check the model's fitness. Results were presented in narrative and tables.

Ethical consideration

Ethical clearance was obtained from the Ethical Review Board of Wolaita Sodo University College of Health Science and Medicine. Formal permission letters for each health facility were written by the Zonal Health Department and Town Health Office in decentralized order. The purpose of the study was explained to the study participants and both informed written and verbal consent from each spousal partner and consent from partners for their biological children plus assent from children aged 12-14 years assent for biological children from their parents were obtained. The information obtained from the study participants was kept confidential. The HIV testing and interview were undertaken privately in a separate scheduled area. Family members who tested negative were counseled about further preventive measures, and those who were positives were positives were linked to a nearby ART clinic where care and support were given according to the national guidelines.

RESULTS

Socio-demographic and economic characteristics of family members of index cases

A total of 623 family members of index cases in Sodo Town were Interviewed, which was a response rate of 94%.interviewed with a response rate of 94%. While the majority, 406 (65.2%), were biological childrenWhile the majority were biological children (406, 65.2%), nearly one-third 217(217, 34.8%) were spousal partners. The mean (SD) age of biological children was 8.9 (+ 3.66) and of the spousal partner was 36.9 (+ 9.26) years.The mean age of biological children was 8.9 ± 3.66 and the spousal partner was 36.9 ± 9.26 years. Of the respondents, nearly half (51.5%) were females, more than half (54.6%) were Orthodox Christians, and one-third (32.6%) of the respondents wealth status in the first quartilehad Frist percentile wealth status. Including 1072 biological children (2–14) years old (1.72 biological children per index case), a total of 2,932 family members (4.71 family members per index case) were identified from 623 index cases (Table 1).

Table 1 Socio-demographic and economic characteristics of **members of index cases in Sodo Town, Southern Ethiopia, 2021(N=623)**family members of index case in Sodo Town, Southern Ethiopia, 2021(n=623).

Variables	Category	Frequency	Percentage
Sex	Male	302	48.5
	Female	321	51.5
Age	<5	87	14.0
	5-9	126	20.2
	10-14	192	30.8
	15-29	43	6.9
	≥>=30	175	28.1
Religion	Orthodox	340	54.6
	Protestant	249	40.0
	Muslim	26	4.2
	Catholic	8	1.3
Wealth index	Frist percentilequartiles	203	32.6
	SecondpercentileSecond quartile	211	33.9
	Third quartilepercentile	209	33.5

Family size	1-3<br>4-6<br>≥>=7	129<br>447<br>47	20.7<br>71.7<br>7.5
2-14 yrs. old biological child size	<3<br>≥>=3	506<br>117	81.2<br>18.8
Alive parents	Father<br>Mother<br>Both	26<br>123<br>474	4.2<br>19.7<br>76.1
Relation to the index	Spouse<br>Biological child	217<br>406	34.8<br>65.2
Even though we expected 651 index cases disclosures from 723 eligible index cases, to determine sample size, 662 were disclosed during data collection and used as the denominator to determine non-response rateused for to determine non-response rate.			

Sexual and substance use behavior of spousal partners of index case

Of the 217 spousal partners, nNearly one-fourth (23%) had 10 or more lifetime sexual partners. The majority (84.8%) were in a monogamous relationship; 69.1% had been living together with their spouse for more than ten years; and (68.2%) had an age difference of ten years or more between spousal partners. Despite the fact that most (76.5%) had been educated on condom use, more than half (60.8%) had never used one during sexual intercourse with their spousal partners. While the majority (78.8%) had never smoked tobacco, 70% had chewed chat, 9.7% had drunk alcohol daily While the majority had never smoked tobacco (78.8%) and chaw chat (70%), 9.7% drank alcohol daily (Table 2).

Table 2 Sexual and substance use behavior of spousal partners of index cases in Sodo Town, Southern Ethiopia, 2021(Nn=217)

Variables	Category	Frequency	Percentage
Number of life time sexual partners	<5<br>5-9<br>≥=/>10	89<br>78<br>50	41.0<br>35.9<br>23.0
How do you describe the type of your current	Polygamous	33	15.2

marriage	Monogamous	184	84.8
Is this your first marriage or partnership	Yes<br>No	28<br>189	12.9<br>87.1
Are you the first spouse for your spouse	Yes<br>No	17<br>200	7.8<br>92.2
Duration of spousal partners living together in year	<5<br>5-9<br>≥>=10	26<br>41<br>150	12.0<br>18.9<br>69.1
Age difference between spousal partners	<5<br>5-9<br>≥>=10	22<br>47<br>148	10.1<br>21.7<br>68.2
Ever discussed condom use with your spousal partner	Yes<br>No	166<br>51	76.5<br>23.5
Ever used a condom during sexual intercourse with your spousal partner	Yes<br>No	98<br>119	45.2<br>54.8
How regularly did you use condoms during sexual intercourse with spousal partner	Never<br>Sometimes<br>Always	132<br>72<br>13	60.8<br>33.2<br>6.0
Ever drunk alcohol	Never<br>Occasionally<br>Daily	42<br>154<br>21	19.4<br>71.0<br>9.7
Ever used Tobacco	Never<br>Occasionally<br>Daily	171<br>26<br>20	78.8<br>12.0<br>9.2
Ever chaw chat	Never<br>Occasionally	152<br>65	70.0<br>30.0

HIV testing history and serostatus of family members of index cases

Of the 267 family members, most (90.9%) ~~Most family members (90.9%)~~ had ever been tested for HIV, and 57 (9.1%) had never ~~not~~ been tested. Of the ever-tested participants, 186 (32.9%)

had positive status. Of those who had never been tested or whose results were negative (437), 10 (2.8%) were newly identified positives. The overall prevalence among family members was 31.5~~0~~% (196/623 cases~~196 out of 623 tested and known results~~). (Table 3)

Table 3: HIV serostatus and testing history-related factors of family members of index cases in Sodo Town, Southern Ethiopia, 2021

Variables	Category	Frequency	Percentage
Have you ever tested for HIV(N=623)	Yes	566	90.9
	No	57	9.1
What was the result if you had been tested(N~~n~~=566)	Positive	186	32.9
	Negative	380	67.1
If never been tested or the result was negative, what is the current result? (practically confirmed)(N~~n~~=437)	Positive	10	2.29
	Negative	427	97.71
Final current HIV serostatus (N=623)	Positive	196	31.5
	Negative	427	68.5

In a sub-group analysis of ~~only~~ the biological children (N=406), 353 (86.9%) had~~have~~ ever been tested, and 53 (13.1%) have not been tested. Of the ever-tested participants, 37 (10.5%) had positive status. Of those who had never been tested or whose results were negative, eight (2.8%) were newly identified positives. Of all the biological child respondents, 45 (11.1%) were positive and 361 (88.9%) were negative~~.~~ (Table 4).

Table 4: HIV serostatus and testing history of biological children of index cases, Sodo Town, Southern Ethiopia, 2021 (N~~n~~=406)

Variables	Category	Frequency	Percentage
Have you ever tested for HIV	Yes	353	86.9
	No	53	13.1
What was the result if you had been tested(n=353)	Positive	37	10.5
	Negative	316	89.5
If never been tested or the result was negative, what is the current result? (practically confirmed)(n=369)	Positive	8	2.17
	Negative	361	97.83
Final current HIV serostatus	Positive	45	11.1

	Negative	361	88.9

In a sub-group analysis of the spousal partners (N=217)In a sub-group analysis of only spousal partners, nearly one-fourth (23%) had STI symptoms or a history of STI in the 12 months prior to data collection. All (100%) heard about HIV testing and counseling service provision and thought testing would be is important to family members of PLHIVs. A majority (98.2%) of them were ever tested, and 149 (70%) had HIV positive status.The majority (98.2%) have been tested, while four (1.8%) have not.ever-tested participants, 149 (70%) had positive status. Of those who had never been tested or whose results were negative, two (3%) were newly identified HIV positive patients.2 (3%) were newly identified positives. Of the spousal partners From all the spousal partner respondents, 151 (69.6%) were positive and were HIV positive and 66 (30.4%) were negative (Table 5)66 (30.4%) were negative. (Table 5)

Table 5: Sexually transmitted InfectionsSTIs and HIV testing history of spousal partners of index cases in Sodo Town, Southern Ethiopia, 2021 (Nn=217)

Variables	Category	Frequency	Percentage
had Had history or symptoms of STIs (N=217)	Yes<br>No	50<br>167	23.0<br>77.0
Heard about HIV testing and counseling service provision to family members PLHIVs(N=217)	Yes<br>No	217<br>0	100<br>0
Do you think testing is important to family members of PLHIVs (N=217)?	Yes<br>No	217<br>0	100<br>0
Have you ever tested for HIV(N=217)?	Yes<br>No	213<br>4	98.2<br>1.8
What was the result if you had been tested (Nn=213)?	Positive<br>Negative	149<br>64	70.0<br>30.0
If never been tested or the result was negative, what is the current result? (practically confirmed) (Nn=68)	Positive<br>Negative	2<br>66	2.94<br>97.06
Final current HIV serostatus (N=217)	Positive<br>Negative	151<br>66	69.6<br>30.4

Characteristics of index cases of family members

From a total of 623 index cases, 386 (62%) disclosed their status to at least one family member within a month of diagnosis, and the majority of 476 (76.4%) started ART immediately as they were eligible. Most, 97% and 79.9%, of Most (97% and 79.9%) of the index cases had current and at enrollment working functional status, respectively. Five hundred fifty eight (89.6%) had a current suppressed viral load and 330 (53%) had a low CD4 count (350 cells/ml) at ART enrollment. The majority, 505 (81.1%) and 406 (65.2%)The majority (505, 81.1%) and (406, 65.2%) of the index cases had no history of treatment failure and poor adherence, respectively. (Table 6).

Table 6: **Clinical and behavioral characteristics of HIV index cases in Sodo town, Southern Ethiopia, 2021 (N=623)**Index case related factors of family members of index cases, Sodo Town, Southern Ethiopia, 2021

Variables	Category	Frequency	Percentage
Notification of disclosure of HIV status of index cases	≥>/1month	237	38.0
	<1month	386	62.0
Immediate ART initiationof index cases	Yes	476	76.4
	No	147	23.6
Functional status at enrollment of index cases	Bedridden	40	6.4
	Ambulatory	85	13.6
	Working	498	79.9
Current functional status of index cases	Bedridden	5	0.8
	Ambulatory	14	2.2
	Working	604	97
WHO stage at enrollment of index cases	Stage One	265	42.5
	Stage Two	167	26.8
	stage Three	152	24.4
	stage Four	39	6.3
Current WHO stage of index cases	Stage One	593	95.2
	Stage Two	25	4.0
	Stage Three	0	0
	stage Four	5	0.8

Duration of infection since known ~~of index cases~~	<60 month<br>60-120 month<br>≥~~>/~~120 month	164<br>189<br>270	26.3<br>30.3<br>43.3
Time to start ART since known infection ~~of index cases~~	≤~~<=~~1 month<br>2-11 month<br>≥~~>=~~12 month	254<br>235<br>134	40.8<br>37.7<br>21.5
Base line CD4~~of index cases~~	<350 cells/ml<br>≥~~>/~~350 cells/ml	330<br>293	53.0<br>47.0
Current Viral Load(VL) status ~~of index cases~~	Unsuppressed<br>Suppressed	65<br>558	10.4<br>89.6
History of treatment failure ~~of index cases~~	Yes<br>No	118<br>505	18.9<br>81.1
History of poor adherence ~~of index cases~~	Yes<br>No	217<br>406	34.8<br>65.2

~~Associated factors~~Factors associated with HIV seropositive status among biological children

The bivariate binary logistic regression found five variables that affected the HIV seropositive status of the family members of index cases of HIV. These were immediate ART initiation if eligible; functional status at enrollment; WHO stage at enrollment; duration of infection since known; and baseline CD4 of index cases of HIV. ~~Immediate ART initiation, if eligible, functional status at enrollment, WHO stage at enrollment, duration of infection since known and baseline CD4 of index cases were found a candidate significantly associated variable for a seropositive status of biological children of index case and their AOR was calculated after these five variables entered together in binary logistic regression model~~ (Table 7).

The m~~M~~ultivariate analysis in binary logistic regression ~~found~~ showed that three out of five variables were significantly associated with the outcome variable. Biological children whose eligible index cases started ART immediately were 85% less likely to be seropositive than those who didn’t (AOR = 0.148; 95%CI: 0.067-0.325). Biological children whose index cases' functional status was bedridden or ambulatory at enrollment were 7.71 times more likely to be seropositive ~~than~~ compared to those whose status was working (AOR = 7.71; 95%CI: 3.5-17).

Biological children whose index case baseline CD4 level was 350 cells/ml were 8.06 times more likely to be seropositive ~~than~~ compared to those whose CD4 level was > 350 cells/ml (AOR = 8.06; 95%CI: 1.8-36)(Table 7).

Table 7: Factors associated with seropositive status among biological children of index cases in Sodo Town, 2021 (N~~n~~=406)

Variables	Category	Serostatus of biological children		95%CI	
		Positive	Negative		
				COR	AOR
Immediate ART initiation ~~of index cases~~	Yes	13(3.2%)	300(73.9%)	0.083(0.041-0.167)	0.148(0.067-0.325)***
	No	32(7.9%)	61(15.0%)	1	1
Functional status at enrollment ~~of index cases~~	Bedridden or Ambulatory	32(7.9%)	51(12.6%)	14.96(7.36-30.4)	7.71(3.5-17)***
	Working	13(3.2%)	310(76.4%)	1	1
WHO stage at enrollment ~~of index cases~~	Stage1 or 2	10(2.5%)	276(68.0%)	1	1
	Stage3 or 4	35(8.6%)	85(20.9%)	11.7(5.4-23.9)	1.53(0.512-4.55)
Duration of infection since known ~~of index cases~~	<60 month	3(0.7%)	95(23.4%)	1	1
	60-120 month	13(3.2%)	121(29.8%)	3.4(0.942-12.3)	0.597(0.119-2.99)
	≥~~>/~~120 month	29(7.1%)	145(35.7%)	6.33(1.88-21.4)	0.853(0.181-4.02)
Base line CD4 ~~of index cases~~	<350	43(10.6%)	167(41.1%)	24.98(5.96-104)	8.06(1.8-36)**
	≥~~>/~~350	2(0.5%)	194(47.8%)	1	1
~~* Statistically significant (p=0.01-<0.05), ** Strong statistical significance (p=0.001-<0.01), *** Very strong statistical significance (p< 0.001)~~					

*** Statistically significant (p=0.01–<0.05), ** Strong statistical significance (p=0.001–<0.01), *** Very strong statistical significance (p< 0.001)**

~~Associated factors~~Factors associated with HIV seropositive status among spousal partners

The bivariate analysis showed sex, wealth index, ever used a condom, had a history or symptoms of STI in the 12 months before the survey among spousal partners and factors from the index cases such as; notification of disclosure status of HIV, ART initiation, if eligible, functional status at enrollment, WHO stage at enrollment and duration of infection since known were candidate variables for multivariable binary logistic regression analysis.

In multivariable logistic regression analysis, had a history or symptoms of STI ((AOR = 5.7; 95% CI: 1.86–17.5)), and from the index factors like notification of disclosure of HIV status (AOR=0.062; 95%CI: 0.024-0.159), ART initiation status(AOR = 0.172; 95%CI: 0.044–0.675), functional status at enrollment, WHO stage at enrollment(AOR = 7.27; 95%CI: 2.37–22.3, and duration of infection since known(AOR = 5.9; 95%CI: 1.8–14.4) were factors significantly associated with HIV seropositive status of spousal partners of index cases.

Spousal partners who had a history or symptoms of STI in the previous 12 months were 5.7 times more likely to be seropositive than their counterparts (AOR = 5.7; 95% CI: 1.86–17.5).Spousal partners whose index cases disclosed their status within a month of diagnosis were 94% less likely to be seropositive than those whose index cases stayed a month or more (AOR=0.062; CI: 0.024-0.159). Spousal partners whose index cases started ART immediately as eligible were 83% less likely to be seropositive than those whose index cases started immediately (AOR = 0.172; 95%CI: 0.044–0.675). Spousal partners whose index cases were WHO stage III or IV at enrollment were 7.27 times more likely to be seropositive than those whose index cases were WHO stage I or II (AOR = 7.27; 95%CI: 2.37–22.3). Spouses of index cases who had known their status for more than 10 years were 5.9 times more likely to be seropositive than those who had known their status for less than 5 years (AOR = 5.9; 95%CI: 1.8–14.4)(Table8).

Table 8: Factors associated with seropositive status among spousal partners in Sodo Town, 2021 (n=217)

Variables	Category	Serostatus of spousal partner		95%CI	
		Positive	Negative	COR	AOR
Sex	Male	76(35.0%)	42(19.4%)	1	1
	Female	75(34.6%)	24(11.1%)	1.73(0.953-3.13)	1.84(0.75-4.52)

Wealth index	Frist percentile	46(21.2%)	23(10.6%)	0.645(0.317-1.31)	0.551(0.193-1.57)
	Second percentile	43(19.8%)	23(10.6%)	0.603(0.295-1.23)	0.345(0.125-1.949)
	Third percentile	62(28.6%)	20(9.2%)	1	1
Ever used a condom	Yes	59(27.2%)	39(18.0%)	1	1
	No	92(42.4%)	27(12.4%)	2.25(1.25-4.06)	1.37(0.561-3.36)
Had or symptoms of an STI	Yes	41(18.9%)	9(4.1%)	2.36(1.07-5.2)	5.7(1.86-17.5)**
	No	110(50.7%)	57(26.3%)	1	1
Notification of Disclosure of index cases	≥>/1month	106(48.8%)	10(4.6%)	1	1
	<1month	45(20.7%)	56(25.8%)	0.076(0.036-0.162)	0.062(0.024-0.159)***
Immediate ART initiation of index cases	Yes	100(46.1%)	63(29.0%)	0.093(0.028-0.312)	0.172(0.044-0.675)*
	No	51(23.5%)	3(1.4%)	1	1
Functional status at enrollment of index cases	Bedridden or Ambulatory	40(18.4%)	2(0.9%)	11.5(2.7-49.3)	1.23(0.198-7.68)
	Working	111(51.2%)	64(29.5%)	1	1
WHO stage at enrollment of index cases	Stage1or 2	86(39.6%)	60(27.6%)	1	1
	Stage3 or 4	65(30.0%)	6(2.8%)	7.56(3.08-18.6)	7.27(2.37-22.3)**
Duration of infection since known of index cases	<60 month	31(14.3%)	35(16.1%)	1	1
	60-120 month	40(18.4%)	15(6.9%)	3(1.4-6.47)	3.04(0.994-9.32)
	≥>/120 month	80(36.9%)	16(7.4%)	5.65(2.74-	5.09(1.8-

				11.6)	14.4)**
~~* Statistically significant (p=0.01–<0.05), ** Strong statistical significance (p=0.001–<0.01), *** Very strong statistical significance (p < 0.001).~~					

*** Statistically significant (p=0.01–<0.05), ** Strong statistical significance (p=0.001–<0.01), *** Very strong statistical significance (p < 0.001).**

DISCUSSION

In this study, the HIV seropositive status among family members was 31.5% (95%CI; 27.6-35.2%).~~(31.5%) with a 95%CI of (27.6-35.2%)~~. This implies that family members of the index cases are at high risk for HIV infection.~~the index case are at high risk of HIV infection~~. The prevalence is consistent with- the findings of studies ~~the study~~ conducted at Felege Hiwot Referral Hospital, Northwest Ethiopia and Zimbabwe, which was 30%,[10] and ~~Zimbabwe~~ 32.6%[12] respectively. However, it is lower lower than the prevalence reported from South Africa of 38% ~~than a study conducted in South Africa 38%~~ [13] and~~. Moreover,~~ higher than the prevalence reported from Malawi of 22% ~~it is higher than the prevalence of 22% in a study done in Malawi~~ [14].

The possible explanations for this difference might be due to variation in socio-demographic and economic characteristics, prevention strategies, and implementation differences across the nations.

In a sub-group-analysis of the prevalence of seropositive status among family members of index cases only on biological children, the prevalence of seropositive status in our study area was found to be 11.1% with a 95%CI of (8.4–14.5%). This finding is consistent with a study done at FelegeHiwot Referral Hospital, Northwest Ethiopia, which found 10.7%[10].

But our finding is much higher compared with other studies, including the Lesotho study that showed a prevalence of 1.4%, Malawi at 4%, and Kenya at 7.4% [15-17].

Possible explanations for this difference might be implementation differences (time of implementation, service coverage) of the prevention of mother-to-child transmission (PMTCT) strategy and socio-demographic and economic differences between nations.

In a sub-group-analysis of the prevalence of seropositive status among family members of index cases only on spousal partners, in our study area, was found to be 69.6% with a 95%CI (63.1-75.6%).

-This finding is almost similar to a study done at FelegeHiwot Referral Hospital, Northwest Ethiopia; which was 71.5%[10]. But, this finding is lower than in a study conducted in SSA

76.23%[18]. On the other side, this finding is higher than a study conducted in Ethiopia, EDHS, 2016 28%[4].

The possible explanation for this wide gap with the national prevalence might be that participants of index cases were the only new index cases in EDHS, 2016. Likewise, this finding is also higher than the study conducted on 37.7% in Nigeria and 61.9% in Tanzania [19, 20].

The possible explanations for this difference might be ART service coverage, prevention, implementation, and monitoring strategy differences from one country to another country.

This finding revealed that biological children whose index cases started ART immediately, based on eligibility criteria, were 85% less likely to be seropositive than those whose index cases didn't start. This might be the fact that initiation of ART increases CD4 and inversely decreases the viral load level of index cases, eventually preventing transmission of HIV to their biological children[9].

This study showed that biological children whose index cases were bedridden or ambulatory functional status at enrollment were 7.7~~1~~ times more likely to be seropositive than those whose status was working. This finding also revealed that biological children whose index case baseline CD4 level was 350 cells/ml were 8.1~~06~~ times more likely to be seropositive than those whose CD4 level was > 350 cells/ml. There is no study report related to this in this specific age group and almost 90% of biological children acquire the HIV infection through vertical transmission. However, the importance of these factors is emphasized by the national PMTCT guideline of Ethiopia~~Even though there is no study on these specific age groups and almost 90% of biological children acquire the infection through vertical transmission from infected mother to child, these factors are supported by the national PMTCT guideline of Ethiopia~~ [9]. In index cases who do not start ART as per the eligibility criteria, their CD4 level declines. This leads to viral replication, increase in vulnerabilities to opportunistic infections, and deterioration in patients functional status, thereby increasing the possibility of vertical viral transmission of HIV. ~~As index cases do not start ART as per the eligibility criteria, the CD4 level declines and lead to viral replication which exposes to different opportunistic infections that finally become a client functional status deterioration and vertical viral transmission to a biological child.~~

This study revealed that spousal partners who had a history or symptoms of an STI in the 12 months before the survey were 5.7 times more likely to be seropositive than their counterparts. It is consistent with the study conducted in 30 provinces of China [21].

The possible explanation might be that since STI and HIV have a linear relationship, and STI may be characterized by genital sores or ulcers, inguinal swelling that is exposed to HIV transmission. This finding is supported by EDHS, 2016 [4].

This finding of this study shows ~~This finding showed~~ that spousal partners whose index cases disclosed their status within a month of diagnosis were 94% less likely to be seropositive than those whose index cases who stayed for a month ~~index cases stayed a month~~ or more after diagnosis. This ~~prevention potential~~ implies that disclosure of their HIV positive status to their

spousal partners disclosure to spousal partners facilitates discussion on HIV and raises spousal partner raises awareness about their risk and need for testingto test. Disclosure also has an important health benefits. It increases social support, fosters closer relationship to others, increases testing uptake, creates good prevention opportunities like, condom utilization, and other prevention methods, and improves treatment adherence and retention, which increases CD4 and suppressessd viral load level that leads to halting of transmission. This finding is supported by the National Comprehensive HIV Care Guideline of Ethiopia, 2018[8].

This finding revealed that those spousal partners whose index cases started ART immediately as on meeting the eligibility criteria eligible by eligibility criteria were 83% less likely to be seropositive than those whose index partners didn't start immediately. This finding is consistent with the findings of a study with a study conducted in Addis Ababa, Ethiopia [22].

This might be because ART initiation based on the eligibility criteria could lead to increase in index case CD4 count and VL suppression that decreases HIV transmission to spousal partners This might be since ART initiation based on the eligibility criteria leads to the index case CD4 increase and viral load level suppression that decreases transmission to his or her spousal partner.

This study showed that spousal partners whose index cases who were at WHO clinical stage III cases had WHO status stage III or IV at enrollment were 7.25 times more likely to be seropositive than spousal partners whose index cases who were WHO clinical stage I or IIcases were WHO stage I or II. This finding is It is consistent with the study conducted in Thailand and Northern Vietnam[23, 24].

This could be due to the fact that index cases who were at WHO stage This is due to the fact that those who were stage III or IV had severe opportunistic infections that exposed the index case to decreased their CD4 levels and increased the viral load levels resulting in decreased CD4 levels and increased viral load levels, which led to high viral transmission to spousal partners.

This study also revealed that spousal partners whose index cases lived with the virus since they knew their status for more than 10 years were 5.09 times more likely to be seropositive than those who knew their status for less than 5 years. It is consistent with the study conducted in Thailand and Rakia, Uganda [23, 25].

This implies that as the duration of the relationship between spousal partners increases, the rate of HIV transmission also increases.

Strength and Limitation of the Study

Since Because the factors of biological children and spousal partners of index cases are different, being able to address both outcomes in one study a separate sub-group analysis was the strength of this study.

Family members whose index cases did not disclosed their status or had follow-up outside of Sodo Town were not included in the study. Obstetric and as well as modes of transmission other than MTCTwere excluded as the study focused on biological factors~~Family members whose index cases hadn't disclosed their status or had follow-up outside of Sodo Town were not included in this study. Obstetric, labor, delivery, and infant factors, as well as other modes of transmission other than MTCT, were excluded as biological child factors~~.

Conclusion

In this study, HIV among family members of index cases in general and specifically in both biological children and spousal partners in Sodo Town is high. ~~,~~L~~l~~ate ART initiation as per the eligibility criteria, bedridden or ambulatory functional status during enrollment, and baseline CD4 350 cells/ml of index cases were the major factors associated with HIV seropositive status~~the major factors of seropositive among biological children of index cases~~. ~~Whereas among~~in the spousal partners with HIV~~-~~history or symptoms of STI, and whereas among the index cases~~from the index case side~~, late notification of disclosure ~~of~~ status, late ART initiation as per the eligibility criteria, WHO stage III or IV at enrollment, and duration of HIV infection since known of > 10 years were factors for the seropositive. In the context of Sodo Town, comprehensive HIV prevention, care, and treatment services need be promoted and implemented among family members of the index cases to increase the achievements of program targets in HIV epidemic control~~So, in the context of the town, holistic efforts to promote the prevention of HIV among family members, including index cases, are critical to ending the HIV epidemic~~.

ACKNOWLEDGMENT

We are grateful to the Wolaita Sodo University, College of Medicine and Health Sciences for providing ethical clearance for this study. We are also indebted to our data collectors, supervisors, and the study participants.

Funding: no funding was received.

Author's contribution

Conceptualization: Alemayehu Kefale

Data curation: Alemayehu Kefale, Dereje Haile, Amene Abebe, Kassa Daka

Formal analysis: Alemayehu Kefale, Dereje Haile, Amene Abebe, Abdulbasit Sherefa

Investigation: : Alemayehu Kefale, Dereje Haile, Amene Abebe, Abdulbasit Sherefa, Animut Adiss, Muluken Gunta, Kassa Daka

Methodolgy: Alemayehu Kefale, Dereje Haile, Amene Abebe, Abdulbasit Sherefa, Animut Adiss, Muluken Gunta, Kassa Daka

Project Administration: Alemayehu Kefale, Animut Adiss, Muluken Gunta

Resources: Alemayehu Kefale, Animut Adiss, Muluken Gunta

Software: Alemayehu Kefale, Dereje Haile, Amene Abebe, Abdulbasit Sherefa, Kassa Daka

Supervision: Alemayehu Kefale, Dereje Haile, Amene Abebe, Abdulbasit Sherefa, Animut Adiss, Muluken Gunta

Validation: Alemayehu Kefale, Dereje Haile, Amene Abebe

Visualization: Alemayehu Kefale, Dereje Haile, Amene Abebe

Writing original draft: Alemayehu Kefale, Dereje Haile

Writing – review & editing: Alemayehu Kefale, Dereje Haile, Amene Abebe, Abdulbasit Sherefa, Animut Adiss, Muluken Gunta

References

1. WHO, *Partner and Family-Based Index Case Testing: A Standard Operating Procedure (SOP)*

2015: p. 3-20.

2. UNAIDS, *FACT SHEET 2021 Global Hiv Statistics.* Ending the AIDS epidemic, 2021(June): p. 1-3.
3. info, U.a.A., *Country factsheets, Ethiopia* Unaids, 2021: p. 1-6.
4. Agency, C.S., *Ethiopian Demographic and Health Survey(EDHS), 2016, HIV Report*. 2018.
5. *Ethiopian Public Health Institute (EPHI). Ethiopia Population-based HIV Impact Assessment*

(EPHIA) 2017-2018: Final Report. Addis Ababa: EPHI; August 2020.

6. CSA, E., WHO, *Min-Ethiopian Demographic Health Survey 2019.* 2019.
7. HAPCO, *HIV Prevention in Ethiopia National Road Map 2018 - 2020.* 2018. **https:// e**(November 2018): p. 25-54.
8. FMOH, E., *National Consolidated Guidelines for Comprehensive Hiv Prevention , Care and treatment.* 2018: p. 1-238.
9. MOH, *National Comprehensive and Integrated Prevention of Mother-Child Transmission of HIV Guideline* 2018. **6**(1).
10. Belayneh, T.W., *Assessment of Tracing Family Members of HIV Positive People for HIV Testing and Associated Factors among ART Clinic Attendants at Felege Hiwot Referral Hospital, Northwest Ethiopia.* Journal of Pharmacy and Alternative Medicine, 2017. **15**: p. 16-28.
11. Tamirat Melis Berhe, L.L., Addissu Alemayehu, Desalegn Ajema, Mustefa Glagn, Samuel Dessu, *HIV-Positive Status Disclosure and Associated Factors among HIV-Positive Adult Patients Attending Art Clinics at Public Health Facilities of Butajira Town Southern Ethiopia.* AIDS Research and Treatment, 2020. **2020**.
12. Nyikadzino Mahachi, A.M., Taurayi A Tafuma, Peter Mawora, Liz Kariuki, Bazghina-werq Semo,Moses H Bateganya, Tendai Nyagura, Getrude Ncube, Mike B Merrigan, Otto N Chabikuli and Mulamuli Mpofu, *Sustained high HIV case-finding through index testing and partner notification services: experiences from three provinces in Zimbabwe.* the International AIDS Society, 2019. **22**(S3): p. 23-30.
13. Dvora Joseph Davey, K.M.W., Claire Serrao, Marlien Prins, Madaline Feinberg, Ntokozo Mtonjana, Khanyo Hlophe, Lindiwe Zuma, Senate Sejake, and Todd Malone, *HIV Positivity and Referral to Treatment Following Testing of Partners and Children of PLHIV Index Patients in*

Public Sector Facilities in South Africa. Journal of Acquired Immune Deficiency Syndromes, 2019. **81**(4): p. 365-370.

14. MALAWI: Improvement, Q.S., F O R, *INDEX CASE TESTING: A PROMISING STRATEGY FOR ACHIEVING HIV EPIDEMIC CONTROL.* Management Sciences for Health, 2018(June): p. 7-10.
15. Makhahliso Jubilee, F.J.P., Knowledge Chipango, Kenoakae Pule,Albert Machinda, Noah Taruberekera, *HIV index testing to improve HIV positivity rate and linkage to care and treatment of sexual partners, adolescents and children of PLHIV in Lesotho.* PLoS ONE, 2019. **14**(3): p. 1-12.
16. Saeed Ahmed, R.A.S., Katie Simon, Nora E. Rosenberg, Elijah Kavuta, Mwelura Harawa, Spencer Dick, Frank Linzie, Peter N. Kazembe and Maria H. Kim, *Index case-finding facilitates identification and linkage to care of children and young adults living with HIV/AIDS in Malawi.* Tropical Medicine and International Health, 2019. **22**(8): p. 1021-1029.
17. Anjuli D WAGNER, C.M.W., Irene N NJUGUNA, Elizabeth MALECHE-OBIMBO, Kenneth SHERR, Irene W INWANI, James P HUGHES, Dalton C WAMALWA, Grace C JOHN-STEWART, and Jennifer A SLYKER, *Active referral of children of HIV-positive adults reveals high prevalence of undiagnosed HIV.* Journal of Acquired Immune Deficiency Syndromes, 2016. **73**(5): p. e83-e89.
18. Mihretie Gedfew, M.D., Belayneh Mengist, Hailie Amaha, Dessalegn Hailie and Bekalu Bewket, *Prevalence of HIV Sero-discordance among Couples in Sub Saharan Africa, 2019, Systematic Review and Meta Analysis.* International Journal of Women's Health Care, 2020. **5**(1): p. 1-9.
19. Semiu Olatunde Gbadamosi, I.U.I., William Nii Ayitey Menson, John Olajide Olawepo, Tamara Bruno, Amaka Grace Ogidi, Dina V. Patel, John Okpanachi Oko, Chima Ariel Onoka, Echezona Edozie Ezeanolue, *Targeted HIV testing for male partners of HIVpositive pregnant women in a high prevalence setting in Nigeria.* PLoS ONE, 2019. **14**(1): p. 1-12.
20. Won, C.K.M.P.A.C.C.B.M.N.R.K.W.M.E.M.R.L.K.C.V., *Addressing the First 90: A Highly Effective Partner Notification Approach Reaches Previously Undiagnosed Sexual Partners in Tanzania.* AIDS and Behavior, 2017. **21**(8): p. 2551–2560.
21. Houlin Tang, Z.W., Yurong Mao, Javier Cepeda, Jamie Morano, *Risk Factor Associated with Negative Spouse HIV Seroconversion among Sero-Different Couples: A Nested Case-Control Retrospective Survey Study in 30 Counties in Rural China.* PLOS ONE, 2016. **11**(10): p. 1-18.
22. Kerebih Bantigen, L.K., Haweni Negeri, Mekonen Kebede, Addisu Wassie, Keralem Bishaw, Getaye Tesema, *Rate of HIV Seroconversion Among Seronegative Male Partners Living with HIV Positive Women.* HIV/AIDS - Research and Palliative Care, 2021. **13**: p. 125–134.
23. Sasisopin Kiertiburanakul, P.W., Angsana Phuphuakrat, Darunee Chotiprasitsakul, Somnuek Sungkanuparph, *Prevalence of HIV infection access to HIV care and response to antiretroviral therapy among partners of HIV infected individuals in Thailand*

PLoS ONE, 2018. **13**(6): p. 1-12.

24. Vu Van Tam, D.D.C., Tobias Alfven, Ho Dang Phuc, Nguyen Thi Kim Chuc, Nguyen Phuong Hoa, Vinod Diwan and Mattias Larsson, *HIV sero‑discordance among married HIV patients initiating anti‑retroviral therapy in northern Vietnam.* AIDS Research and Therapy, 2016. **13**(1): p. 1-7.
25. Nabukenya, A.M., Nambuusi, Aminah, Matovu, Joseph K.B, *Risk factors for HIV infection among married couples in Rakai, Uganda: A cross-sectional study.* BMC Infectious Diseases, 2020. **20**(1): p. 1-8.

Manuscript #: PONE-D-22-04620

Title: Seropositive Status and Associated Factors among Family Members of Index Cases of ART clinic Attendants in Sodo Town, Southern Ethiopia

Comments to the Author

1. Is the manuscript technically sound, and do the data support the conclusions?

The manuscript must describe a technically sound piece of scientific research with data that supports the conclusions. Experiments must have been conducted rigorously, with appropriate controls, replication, and sample sizes. The conclusions must be drawn appropriately based on the data presented.

Reviewer #1: Yes

---

2. Has the statistical analysis been performed appropriately and rigorously?

Reviewer #1: Yes

---

3. Have the authors made all data underlying the findings in their manuscript fully available?

The PLOS Data policy requires authors to make all data underlying the findings described in their manuscript fully available without restriction, with rare exception (please refer to the Data Availability Statement in the manuscript PDF file). The data should be provided as part of the manuscript or its supporting information, or deposited to a public repository. For example, in addition to summary statistics, the data points behind means, medians and variance measures should be available. If there are restrictions on publicly sharing data—e.g. participant privacy or use of data from a third party—those must be specified.

Reviewer #1: Yes

---

4. Is the manuscript presented in an intelligible fashion and written in standard English?

PLOS ONE does not copyedit accepted manuscripts, so the language in submitted articles must be clear, correct, and unambiguous. Any typographical or grammatical errors should be corrected at revision, so please note any specific errors here.

Reviewer #1: No

---

5. Review Comments to the Author

Please use the space provided to explain your answers to the questions above. You may also include additional comments for the author, including concerns about dual publication, research ethics, or publication ethics. (Please upload your review as an attachment if it exceeds 20,000 characters)

General Comments:

focused objective and has generated the required data and performed an appropriate analysis of the data and interpretation of the results. It has generated conclusions warranted by the data. However, the language of the submitted manuscript for from adequate, both from the perspective of clearly conveying what is intended and quality standards of scientific publications. Here, review comments are provided that the authors may wish to consider improving the quality of the manuscript, particularly in terms of content organization, formatting, and language to make the manuscript potentially publishable in PLOS.

Authors need to use the 'PLoS Guidelines to Authors' in the formatting (line spacing, text alignment) as well as in organizing the content in the different sections of the manuscript. Please note that some of the comments given in the different sections below clearly indicate this.

The manuscript requires heavy language editing – authors may wish to enlist support from a language editor. Much language editing in the manuscript is done as part of this review (please see below comments in each section)

Thank you for your patience and meticulous observation of this manuscript and your point-by-point comments. We have carefully addressed the comments raised accordingly.

Specific comments:

Title/Title Page

The title should be written in sentence case (capitalize only the first word of the title and proper names); expand ART to Antiretroviral Treatment

Response: thank you for your comment; we have corrected and highlighted it in the revised manuscript

Line 1: Missing space between Seropositiveand Status

Response: thank you for your comment; we have corrected and highlighted it in the revised manuscript

Line 3: Missing space between words. Correct CLINICATTENDANTSINSODO to Clinic Attendants in Sodo

Response: thank you for your comment; we have corrected and highlighted it in the revised manuscript

Line 4: Would edit Abebe [2]Dereje asread Abebe[2], Dereje

Response: thank you for your comment; we have corrected and highlighted it in the revised manuscript

Line 5: Would remove the extra comma between Addis[4] and Muluken

Response: thank you for your comment; we have corrected and highlighted it in the revised manuscript

Lines 6-9: Would edit the affiliation for each author (include department, University, or organization affiliation, and its location, including city, state/province, and country

Response: thank you for your comment; we have corrected and highlighted it in the revised manuscript

Would add on this page: first author's e-mail address followed by name initials in parenthesis

Response: thank you for your comment; we have corrected and highlighted it in the revised manuscript

On this page: also indicate authors' contribution(s) using symbols as given in PLoS One "Title, Author, Affiliation Formatting Guidelines".

Abstract:

Lines 30-31: the sentence needs editing; suggest "... transmitted through … sexual contact with an infected partner and babies born to mothers infected with the virus."

Response: thank you for your comment; we have corrected and highlighted it in the revised manuscript

Lines 33-35: The sentence is the title repeated as is; suggest restating this in a form of an objective ... Like "This study aimed at identifying prevalence and determinants of HIV infection among family members of index cases on antiretroviral treatment (ART)."

Response: thank you for your comment; we have corrected and highlighted it in the revised manuscript

Line 38: Would Insert HIV between "of" and "index"

Response: thank you for your comment; we have corrected and highlighted it in the revised manuscript

Line 39: ".. collected data ……" on what ?

Response: thank you for your comment; we have corrected and highlighted it in the revised manuscript

Line 39: Would add space after the stop after data.

Response: thank you for your comment; we have corrected and highlighted it in the revised manuscript

Line39: Would replace "investigate" with "identify"

Response: thank you for your comment; we have corrected and highlighted it in the revised manuscript

Line40: Helpful to add the expanded form for AOR upfront here so that we use the acronym in the rest f the manuscript; i.e., adjusted odds ratio (AOR)

Response: thank you for your comment; we have corrected and highlighted it in the revised manuscript

Line41: Same here. 95% Confidence interval (CI)

Response: thank you for your comment; we have corrected and highlighted it in the revised manuscript

Lines41: The P in P-values is written in upper case as is and italicised (*P*). Please do this across the manuscript.

Response: thank you for your comment; we have corrected and highlighted it in the revised manuscript

Lines41-42: last part of the sentence requires editing. Suggest changing "----used to declare the cut-off point in determining the level of significance" to "---- used as a cut-off point to determine the level of statistical significance of point estimate."

Response: thank you for your comment; we have corrected and highlighted it in the revised manuscript

Line 45:Would edit"----- revealed that 31.5% with 95%CI (27.6-35.2%)' as "----- revealed that 31.5% (95%CI: 27.6-35.2%); need to use same formant across the manuscript.

Response: thank you for your comment; we have corrected and highlighted it in the revised manuscript**.**

Lines 46-47: Would edit:(…11.1% with 95%CI (8.4–14.5%..) as (…11.1% (95%CI: 8.4–14.5%)

Response: thank you for your comment; we have corrected and highlighted it in the revised manuscript

Line 47:Would edit:"…and 69.6% with 95%CI (63.1–75.6%..)" as"….. and 69.6% (95%CI: 63.1–75.6%)

Response: thank you for your comment; we have corrected and highlighted it in the revised manuscript

Line 50:Would insert 'of' between "level" and "350"

Response: thank you for your comment; we have corrected and highlighted it in the revised manuscript

Line 51:Would edit "….. significant with …." as "….. significantly associated with …"

Response: thank you for your comment; we have corrected and highlighted it in the revised manuscript

Line 54:Would edit:"…. infection since known …", may remove 'since known' as this does not add an y useful information.

Response: thank you for your comment; we have corrected and highlighted it in the revised manuscript

Line 59:Would change 'should be' to 'might be' or 'need to'; one needs to avoid strong words like should in conclusions/recommendations.

Response: thank you for your comment; we have corrected and highlighted it in the revised manuscript**.**

Introduction

Line 60: May consider editing as "HIV infection, HIV seropositive, biological children, spousal partners, index case,".

Response: thank you for your comment; we have corrected and highlighted it in the revised manuscript**.**

Line 63: Definition is incomplete, and the language requires editing. Option: "Index testing is a voluntary process where counsellors or health care providers ask a newly diagnosed HIV positive patient or an HIV

positive patient already accessing HIV treatment to list all partners who could have been exposed to HIV through them, the index cases" [1].

Response: thank you for your comment; we have corrected and highlighted it in the revised manuscript**.**

Line 57: Just use the acronym ‘ICT’; already used at first enter above with the expanded forms.

Response: thank you for your comment; we have corrected and highlighted it in the revised manuscript**.**

Line 67: Insert space “ ….models of testing:(1)Facility…..” so that it reads“ ….models of testing (1) Facility…..”

Line:67:Would edit “… (1)Facility Based ICT …” as “ ……. (1) Facility-based ICT …”

Response: thank you for your comment; we have corrected and highlighted it in the revised manuscript**.**

Line 68; Would edit “…. index case and gets the names ..” to read “…. index cases on ART at the health facility, and gets the names .. “

Response: thank you for your comment; we have corrected and highlighted it in the revised manuscript**.**

Line 69: Would replace ‘old’ with “of age’

Response: thank you for your comment; we have corrected and highlighted it in the revised manuscript**.**

Line 69: Would Insert a comma after ‘old’ and delete “in Antiretroviral Treatment (ART) service giving facility.

Response: thank you for your comment; we have corrected and highlighted it in the revised manuscript**.**

Line 69-72: Sentence after ‘(2)’ needs revision, as it has many grammatic errors; suggest: “community-based ICT: a counsellor traces sexual partner(s) of index cases and their biological children who are under 15 years of age through home-to-home visits, offers HTS, and links newly identified People Living with HIV (PLHIV) to ART services [1].

Response: thank you for your comment; we have corrected and highlighted it in the revised manuscript**.**

Line 75. Would add ‘year’ at the end of the sentence.

Response: thank you for your comment; we have corrected and highlighted it in the revised manuscript**.**

Line 76. Paragraph starting with this line is a continuation of the above; Would keep this para with the above.

Response: thank you for your comment; we have corrected and highlighted it in the revised manuscript**.**

Line 76: Would edit “….. PLHIV were lived and …” as “…. PLHIV were living in 2021, and of which ….”

Response: thank you for your comment; we have corrected and highlighted it in the revised manuscript**.**

Line 77: In “… transformative and ---” would add ‘a’ before transformative.

Response: thank you for your comment; we have corrected and highlighted it in the revised manuscript**.**

Line 78: Would edit " …. expansion of ART indicating that from total of …."as "…… as indicated by the total number of …"

Response: thank you for your comment; we have corrected and highlighted it in the revised manuscript**.**

Line 79: Would edit "…. sixteen million (16 million) …" as "..16 million…"

Response: thank you for your comment; we have corrected and highlighted it in the revised manuscript**.**

Line 79: Would need space between treatment and the citation in"…. on treatment[2] ….".Would do this consistently all along.

Response: thank you for your comment; we have corrected and highlighted it in the revised manuscript**.**

Line 80: Would replace "…among which …" by "… of which …."

Response: thank you for your comment; we have corrected and highlighted it in the revised manuscript**.**

Line 82: Would keep this paragraph starting with line with the one above paragraph.

Response: thank you for your comment; we have corrected and highlighted it in the revised manuscript**.**

Line 82: Would delete 'also"

Response: thank you for your comment; we have corrected and highlighted it in the revised manuscript**.**

Line 82.Would replace …among which…." With "… of which …"

Response: thank you for your comment; we have corrected and highlighted it in the revised manuscript**.**

Line 83: Would edit "… of age, with 13,000 …" to read " of age. There were 13,000…."

Response: thank you for your comment; we have corrected and highlighted it in the revised manuscript**.**

Line 83: Would edit " …. under 15 years old." as "….. under 15 years of age. …."

Response: thank you for your comment; we have corrected and highlighted it in the revised manuscript**.**

Line 84: Would edit "PLHIVs" should be "PLHIV"

Response: thank you for your comment; we have corrected and highlighted it in the revised manuscript**.**

Line 86: Would merge the paragraph starting with "according to the Ethiopian …." with the above paragraph

Response: thank you for your comment; we have corrected and highlighted it in the revised manuscript**.**

Line 87: Would edit "….was 0.9 percent (0.7 percent …." as "… was 0.9% (0.7% …..", and be consistent all along.

Response: thank you for your comment; we have corrected and highlighted it in the revised manuscript**.**

Line 88: Would replace “With this general prevalence, the current epidemiology of HIV is heterogeneous, with ….” with 'HIV/AIDS in Ethiopia is regularly categorized as “generalized“ among the adult population, with …….”

Response: thank you for your comment; we have corrected and highlighted it in the revised manuscript**.**

Line 90: Would replace “…. Geographical ….” with ““…. Geographic ….”

Response: thank you for your comment; we have corrected and highlighted it in the revised manuscript**.**

Line 90: Would insert a reference (e.g., the Ethiopian Population-based HIV Impact Assessment 2020) at end of the line (after subgroups)

Response: thank you for your comment; we have added and highlighted it in the revised manuscript**.**

Line 92: Would insert after “below 0.1” a reference (e.g., EPHI, Mini EDHS 2018/19).

Response: thank you for your comment; we have corrected and highlighted it in the revised manuscript**.**

Line 92: Would replace ‘Somalia” with ‘Ethiopian Somali Region”

Response: thank you for your comment; we have corrected and highlighted it in the revised manuscript**.**

Line 97:Paragraph starting with this is redundant, repeating what is covered in the above paragraph. Would delete.

Response: thank you for your comment; we have deleted and highlighted it in the revised manuscript**.**

Line 101: Would insert HIV between universal and testing and edit “ …. testing strategy …”, as“…..test and treat strategy ….”

Response: thank you for your comment; we have corrected and highlighted it in the revised manuscript**.**

Line 101: Would edit “…. testing all individuals as a mass ……” as“ …. testing all individuals in the population who were at risk …”

Response: thank you for your comment; we have edited and highlighted it in the revised manuscript**.**

Line 102: Would edit :…. index case clients are one of these areas [6] …” as“….index cases as a target population for this strategy [6].

Response: thank you for your comment; we have edited and highlighted it in the revised manuscript**.**

Line 104: Would keep paragraph starting with this line with the above one.

Response: thank you for your comment; we have corrected and highlighted it in the revised manuscript**.**

Line 105: Would edit “….and Family-Based Index Case Testing ….” as“…..Family-based Index Case Testing ……”

Response: thank you for your comment; we have corrected and highlighted it in the revised manuscript**.**

Line 105: Would edit “……. or Index Case Testing (ICT) strategy ….” as “……. or the ICT strategy ….”

Response: thank you for your comment; we have corrected and highlighted it in the revised manuscript**.**

Line 107: Would edit "…..under-15-year old…" to"…..under 15-year-old…"

Response: thank you for your comment; we have corrected and highlighted it in the revised manuscript**.**

Line 107: Would edit: "…… biological children to break the chain of ……. " as "…… biological children. This breaks the chain of ……. " NB. This breaks a long sentence.

Response: thank you for your comment; we have corrected and highlighted it in the revised manuscript**.**

Line 108: Would edit: "….. who have been exposed to HIV …"as"….. who are exposed to HIV …"

Response: thank you for your comment; we have corrected and highlighted it in the revised manuscript**.**

Line 108: Would edit "….. to HIV to give preventive ….." as "to HIV, provide preventive …."

Response: thank you for your comment; we have corrected and highlighted it in the revised manuscript**.**

Line 109: Would edit "…… services for negative and, …." as"…… services for those testing HIV-negative, and, ….

Response: thank you for your comment; we have corrected and highlighted it in the revised manuscript**.**

Line 109: Would edit "…. if positive, linkage and treatment that increase case detection and attain …." as ". if positive, link them to cared and treatment services, a cascade that will contribute to attaining the.."

Response: thank you for your comment; we have corrected and highlighted it in the revised manuscript**.**

Lines 110-111: Would edit "…. while family members have an ongoing risk of contracting HIV." as"….. exposing the family members to an ongoing risk of contracting HIV."

Response: thank you for your comment; we have corrected and highlighted it in the revised manuscript**.**

Line 111:Would replace "So" with "Therefore"

Response: thank you for your comment; we have corrected and highlighted it in the revised manuscript**.**

Lines 112-113: Would edit "….under 15 years of biological children[1]." as "….under 15-years old biological children[1]."

Response: thank you for your comment; we have corrected and highlighted it in the revised manuscript**.**

Line 117: The paragraph starting with this line would rather be kept with the above one.

Response: thank you for your comment; we have corrected and highlighted it in the revised manuscript**.**

Line 120-122: Would replace "Testing for HIV and linking to care for those identified by the families of the index cases will improve the health outcomes of the seropositive family members[5]." with "Testing for HIV and linking those who are positive to care and treatment services will improve the health outcomes of the seropositive family members[5].

Response: thank you for your comment; we have corrected and highlighted it in the revised manuscript**.**

“Line 123: Would replace ‘But’ with :Nevertheless’

Response: thank you for your comment; we have corrected and highlighted it in the revised manuscript**.**

Line 123: Would edit “HIV positive” as “HIV-positive”; and do this consistently across the manuscript

Response: thank you for your comment; we have corrected and highlighted it in the revised manuscript**.**

Lines 123-127: “…people for HIV testing and associated factors among ART clinic attendants at FelegeHiwot Referral Hospital, Northwest Ethiopia, reveals that 25.8% of index cases were not tested. Even though there were a significant number of family members not tested in this study, 30% of family members were seropositive[8].” requires editing; Would consider “……. ART clinic attendants at a referral hospital in Northwest Ethiopia revealed that many children and adults were traced and tested for HIV. Yet, there were many untested partners, children and other individuals living with index cases who were not tested.

Response: thank you for your comment; we have edited and highlighted it in the revised manuscript**.**

Lines 128-130: Would delete this para; already addressed in the paragraphs above.

Response: thank you for your comment; we have deleted and highlighted it in the revised manuscript**.**

Line131: Paragraph starting with this line would rather be kept with paragraph above ending with ref #8.

Response: thank you for your comment; we have edited and highlighted it in the revised manuscript**.**

Line 131: Would edit “…. no study was conducted …” as“… no similar study was conducted …”

Response: thank you for your comment; we have edited and highlighted it in the revised manuscript**.**

‘Line 134: Would replace ‘’would’ with “will”

Response: thank you for your comment; we have replaced and highlighted it in the revised manuscript**.**

Lines 134-136: “ ….. the family members' information in routine clinical practice and could facilitate ending the HIV/AIDS epidemic vision in 2030.” requires editing; would suggest: :… 'HIV serostatus information on family members of index cases, which will contribute to improving HIV care and testament services and epidemic control in Ethiopia'.

Response: thank you for your comment; we have edited and highlighted it in the revised manuscript**.**

Methods

Line 138: Usually written as ‘Materials and Methods” or just “Methods”

Response: thank you for your comment; we have edited and highlighted it in the revised manuscript**.**

Line 142: Would expand SNNPR. Southern Nations, Nationalities, and Peoples Region (SNNPR).

Response: thank you for your comment; we have expanded and highlighted it in the revised manuscript**.**

Line 142: Would edit "… Sodo Town has been…" as"… Sodo Town is …"

Response: thank you for your comment; we have edited and highlighted it in the revised manuscript**.**

'Line 143: Would replace '3' with 'three'

Response: thank you for your comment; we have replaced and highlighted it in the revised manuscript**.**

Line 143: Would edit as " … kebele (lowest administrative entity)"

Response: thank you for your comment; we have edited and highlighted it in the revised manuscript**.**

Line 144: Would insert reference for "According to the 2021 population and housing census projection [ ]. …"

Response: thank you for your comment; we have edited and highlighted it in the revised manuscript**.**

Line 146: Would replace '3' with 'three'

Response: thank you for your comment; we have edited and highlighted it in the revised manuscript**.**

Line 146: Would edit: ".. service-giving facilities …." as".. service-delivery facilities …."

Response: thank you for your comment; we have edited and highlighted it in the revised manuscript**.**

Line 147: Would replace '2" with 'two" and '1' with 'one'

Response: thank you for your comment; we have edited and highlighted it in the revised manuscript**.**

Line 150: Would replace sentence with "The study was a community-based cross-sectional study"

Response: thank you for your comment; we have edited and highlighted it in the revised manuscript**.**

Line 151: Would edit as "source population"

Response: thank you for your comment; we have edited and highlighted it in the revised manuscript**.**

Line 152-153: The source population of this study seems all patients coming to the health facilities involved in this study. May consider editing the sentence accordingly.

Response: thank you for your comment; we have edited and highlighted it in the revised manuscript**.**

Line 155-156: The study subjects seem "All index cases coming at ART clinics at the study health facilities and their family members.". May consider editing the sentence accordingly.

Response: thank you for your comment; we have edited and highlighted it in the revised manuscript**.**

Line 162-163: May edit as "An index case living in Sodo who at least had one biological child aged 2–14 years or a spousal partner".

Response: thank you for your comment; we have edited and highlighted it in the revised manuscript**.**

Line 164-165: Would delete'living in Sodo'as it is included in the above sentence.

Response: thank you for your comment; we have deleted and highlighted it in the revised manuscript**.**

Line 169: Would add after illness “or not willing to participate.”

Response: thank you for your comment; we have added and highlighted it in the revised manuscript**.**

Line 171-172: May consider editing the sentence as “The ART clinics at all three health facilities in Sodo Town (Sodo Health, Sodo Teaching and Referral Hospital, and Sodo Christian General Hospital) were included as study sites.

Response: thank you for your comment; we have edited and highlighted it in the revised manuscript**.**

Line 173: Would edit “… HIV positive clients …” as“… HIV-positive patients…”

Response: thank you for your comment; we have edited and highlighted it in the revised manuscript**.**

Line 173: Would delete “(COA)”, not a conventional acronym/abbreviation.

Response: thank you for your comment; we have deleted and highlighted it in the revised manuscript**.**

Line 175: Would edit “…and among these 2,213…” as “…. And, among these, 2,213…”

Response: thank you for your comment; we have edited and highlighted it in the revised manuscript**.**

Line 175: Would delete ‘old’

Response: thank you for your comment; we have deleted and highlighted it in the revised manuscript**.**

Line 176: May change ‘Primarily’ to ‘As a first step’

Response: thank you for your comment; we have edited and highlighted it in the revised manuscript**.**

Line 176: Would replace “Partner and Family-Based Index Case testing” with “PFBICT’ – you already have this in line 105 above.

Response: thank you for your comment; we have replaced and highlighted it in the revised manuscript**.**

Line 177: Would delete ‘only’ in :.. clients only living ..”

Response: thank you for your comment; we have deleted and highlighted it in the revised manuscript**.**

Line 177: Would edit “… in Sodo Town, and there were..” as“… in Sodo Town, which were…..”

Response: thank you for your comment; we have edited and highlighted it in the revised manuscript**.**

Line 177: Would edit “Secondly, by using HIV….” as “By using HIV….”

Response: thank you for your comment; we have edited and highlighted it in the revised manuscript**.**

Line 178: Would edit “….we identified 834…”…. as we then identified 834…”

Response: thank you for your comment; we have edited and highlighted it in the revised manuscript.

Line 178: Would edit "….. Then, from the total ….." as "Of the total …."

Response: thank you for your comment; we have edited and highlighted it in the revised manuscript.

Line 180: Would edit "The ART code numbers of facilities were used to identify each facility….." as "The unique ART code numbers given by the national program to the facilities were used to identify each study facility."

Response: thank you for your comment; we have edited and highlighted it in the revised manuscript.

Line 184: Would edit "… By considering this from 723 index clients,…" as : Of 723 index patients, …"

Response: thank you for your comment; we have edited and highlighted it in the revised manuscript.

Line 184: Would edit "..to not.." as "… not to…"

Response: thank you for your comment; we have edited and highlighted it in the revised manuscript.

Line 186: Would edit "…. and selected them as total index cases."

Response: thank you for your comment; we have edited and highlighted it in the revised manuscript.

Line 187: Would edit "…. for a total…" as "… and, as a result, had a total …"

Response: thank you for your comment; we have edited and highlighted it in the revised manuscript.

Line 196: Would remove the "hyphen"

Response: thank you for your comment; we have removed and highlighted it in the revised manuscript.

Line 198: Would remove the "hyphen"

Response: thank you for your comment; we have removed and highlighted it in the revised manuscript.

Line 199: Would edit "VL" as "viral load (VL)"

Response: thank you for your comment; we have edited and highlighted it in the revised manuscript.

Line 200: Would remove the "hyphen"

Response: thank you for your comment; we have edited and highlighted it in the revised manuscript.

Line 202: Would insert a "comma: after 'sexual partners'

Response: thank you for your comment; we have inserted and highlighted it in the revised manuscript.

Line 203: Would remove the "hyphen:

Response: thank you for your comment; we have removed and highlighted it in the revised manuscript.

Lines 209-210: “After addressing the disclosure issue, data collectors were appointed to index cases with their family members for visiting home.” requires editing. Would suggest: “Following disclosure of HIV status by the index cases, data collectors fixed appointments with them and their families for home visits.”

Response: thank you for your comment; we have edited and highlighted it in the revised manuscript**.**

Line 210: Would edit “…Then, during visiting, …:” as :.. At the start of the visit, …"

Response: thank you for your comment; we have edited and highlighted it in the revised manuscript**.**

Line 211: Would edit “… consent for spousal partners…” as “… consent obtained from spousal partners…”

Response: thank you for your comment; we have edited and highlighted it in the revised manuscript**.**

Lines211-212: Would edit “… and assent for biological children was taken….” as “Consent for all biological children was obtained from the parents, and assent was obtained from children 12-14 years of age…”

Response: thank you for your comment; we have edited and highlighted it in the revised manuscript**.**

Line 212:Would edit “Positive serostatus participants were only interviewed, but …”as “In those who were HIV-positive, only interviews were adminste4ed by the investigators, but ...”

Response: thank you for your comment; we have edited and highlighted it in the revised manuscript**.**

Line 213: Would edit “…testing and interview were undergone simultaneously…” as “…..the participants were interviewed and tested for HIV.”

Response: thank you for your comment; we have edited and highlighted it in the revised manuscript**.**

Line 213: Would delete “or guardians”, otherwise, the word biological children should be removed from the manuscript and parent/guardian used consistently all along.

Response: thank you for your comment; we have removed and highlighted it in the revised manuscript**.**

Lines 214-215: “The testing procedure was strictly followed by the current national testing algorithm” requires editing; would suggest “The testing procedure strictly followed the current national testing algorithm.”

Response: thank you for your comment; we have edited and highlighted it in the revised manuscript**.**

Lines 215-216: “For the data of the index case, unique ART numbers were used to review secondary data and link it with the primary data of their family members.” requires editing; Would suggest “Unique ART numbers were used to link primary index case data with that of the family members and with their secondary data in patients' records for analysis.”

Response: thank you for your comment; we have edited and highlighted it in the revised manuscript**.**

Lines 218-231:cosnsitentlu use colons (:) after the bolded words to be defined. Would remove the "hyphens"

Response: thank you for your comment; we have removed and highlighted it in the revised manuscript**.**

Line 218: Would delete "old"

Response: thank you for your comment; we have deleted and highlighted it in the revised manuscript**.**

Line 222: Would edit "…..algorithmic test" as :….. algorithm"

Response: thank you for your comment; we have edited and highlighted it in the revised manuscript

Line 225-231: Would replace equal '=' with "comma"

Response: thank you for your comment; we have replaced and highlighted it in the revised manuscript

Line 225: Would edit "…. share his status to his family members…" as "…. share their status to their family members…"

Response: thank you for your comment; we have edited and highlighted it in the revised manuscript

Line 226: Would edit "…one month, late …" as "…one month, and late …"

Response: thank you for your comment; we have edited and highlighted it in the revised manuscript

Line 228: Would replace "&' with and.

Response: thank you for your comment; we have replaced and highlighted it in the revised manuscript

Line 229: Would remove '=' before immediate/

Response: thank you for your comment; we have removed and highlighted it in the revised manuscript

Line 234: Would edit: "…. The English version was translated …" as "…. The English version of the questionnaire was translated …"

Response: thank you for your comment; we have edited and highlighted it in the revised manuscript

Line 234: Edit "… to the…." as "… into …."

Response: thank you for your comment; we have edited and highlighted it in the revised manuscript

Lines 235-236: "… for gathering information ….." as "….to collect data …."

Response: thank you for your comment; we have edited and highlighted it in the revised manuscript

Line 236: "Five percent of the questioners were pre-tested in the Boditi…." .. not clear. Is it to say 'the questionnaire was pre-tested by the data collectors and the supervisors in 5% of the sample at Boditi Health Center, a site similar to the study health facilities?

Response: the questionnaire was pre-tested by the data collectors in 5% of the sample at Boditi Health center.

Line 237: Would edit "Then it was modified or contextualized." as "The results were used to modify the questionnaire as appropriate."

Response: thank you for your comment; we have edited and highlighted it in the revised manuscript

Line 239-240: Requires editing. Would suggest "… two-day training introduced the objective of the study, familiarized data collectors with the questionnaire, the sampling procedure and interviewing techniques, and the HIV testing procedures …"

Response: thank you for your comment; we have edited and highlighted it in the revised manuscript

Line 240-242: Would delete "The data collectors and supervisors participated in pre-testing of the questionnaire for its understanding, which was not included in the study, and the result was used to correct unclear ideas and statements." As this is incorporated covered as in the edited above.

Response: thank you for your comment; we have deleted and highlighted it in the revised manuscript

Line:343-243: You may edit "During data collection, the supervisors…." as "The supervisors …."

Response: thank you for your comment; we have edited and highlighted it in the revised manuscript

Line 245: Requires editing. Would suggest "After data collection, the collected information was" is replaced by "The collected data were …."

Response: thank you for your comment; we have edited and highlighted it in the revised manuscript

Line 235: Would delete "its: and in line 146 delete the "s" in principal investigators.

Response: thank you for your comment; we have deleted and highlighted it in the revised manuscript

Line 250-259: This section requires revision. Would suggest revising as "EPI data 4.2 statistical software was used for data entry, cleaning, and codingof the variables. The data were exported to SPSS version 25 for analysis. Descriptive statistics were used to summarize the data and check for the distribution of the study variables.A bivariate analysis was done by using a binary logistic regression. A*P*-value of 0.25 was used as a cut-off point to select candidate variables for multivariate logistics regression, the analysis used to determine the association between the independent and dependent variables having controlled for confounding effects. AORwith 95%CI was used to determine the level association. The Hosmer and Lemeshow's goodness of fit test was used to check for model fitness. A variance inflation factor (VIF)or tolerance test was done to check for correlation. A *P*-value of 0.05 was used as a cut-off pointto determine statistical significance and the results were presented in narratives and tables."

Response: thank you for your comment; we have revised and highlighted it in the revised manuscript

Line 263: Would delete "in decentralized order"

Response: thank you for your comment; we have deleted and highlighted it in the revised manuscript

Line 265: Would edit "…. assent for biological children from their parents…." as "…consent from partners for their biological children plus assent from children aged 12-14 years …"

Response: thank you for your comment; we have edited and highlighted it in the revised manuscript

Lines 266-267: Would edit "…. The testing and…." as "…. HIV testing and…."

Response: thank you for your comment; we have edited and highlighted it in the revised manuscript

Line 267: Would replace"…. separate .." with"…. secluded …"

Response: thank you for your comment; we have replaced and highlighted it in the revised manuscript

Line 268: Would edit "…. and positives were …." as "…. and those who were positives were …."

Response: thank you for your comment; we have edited and highlighted it in the revised manuscript

Results

Line 272: Would edit "… "…. interviewed with a response rate of 94%." as ""…. Interviewed, which was a response rate of 94%."

Response: thank you for your comment; we have edited and highlighted it in the revised manuscript

Line 273:Would edit "…While the majority were biological children (406, 65.2%),.." as While the majority, 406 (65.2%), were biological children…"

Response: thank you for your comment; we have edited and highlighted it in the revised manuscript

Lines 273-274: Would edit "… nearly one-third (217, 34.8%) " as "… nearly one-third, 217 (34.8%), …."

Response: thank you for your comment; we have edited and highlighted it in the revised manuscript

Lines 274-275: Would edit: "The mean age of biological children was 8.9 + 3.66 and the spousal partner was 36.9 + 9.26 years. …" as "The mean (SD) age of biological children was 8.9 (+ 3.66) and of the spousal partner was 36.9 (+ 9.26) years.

Response: thank you for your comment; we have edited and highlighted it in the revised manuscript

Lines 276- 277: "… had Frist percentile wealth status"; is this to mean a wealth status in the first quartile?

Response: thank you for your comment; yes, we have edited and highlighted it in the revised manuscript

Lines 277:278: Need to watch consistent use of comas or no commas in numbers (e, g,, 1072 and 2,932). Also across the manuscript

Response: thank you for your comment; yes, we have edited and highlighted it in the revised manuscript

Lines 280-281 (Table 1): Would edit as "…. members of index cases in Sodo Town, Southern Ethiopia, 2021(N=623).* You may move the content in the last row of the table and add it as a footnote starting with an asterisk (*) to link it with the title.

Response: thank you for your comment; yes, we have edited and highlighted it in the revised manuscript

Table 1: Do not understand the categories for the wealth "Wealth Index". I am wondering whether these are quartiles or centiles.

Response: thank you for your meticulous observation, we have edited and highlighted in the revised manuscript.

Table 1: Would insert ≥ symbol (from the Symbols menu) in place of >=

Table 1 (narrative in the last row) : "expected651" needs a space. Would remove the comma in "… cases, to …." as "N' we have 623 in the title and 662 here? Edit "…. used for to determine non-response rate." as "…. used as the denominator to determine non-response rate."

Response: thank you for your comment; yes, we have edited and highlighted it in the revised manuscript

Line 283: Would edit "… index case." as "…index cases."

Response: thank you for your comment; yes, we have edited and highlighted it in the revised manuscript

Line 284: Would provide the denominator upfront here like "Of the 217 spousal partners, nearly ….

Response: thank you for your comment; yes, we have edited and highlighted it in the revised manuscript

Line 288: Would edit "… with their spousal partner." as "… with their spousal partners."

Response: thank you for your comment; we have edited and highlighted it in the revised manuscript

Lines288-289: Would edit as "While the majority (78.8%) had never smoked tobacco, 70% had chewed chat, 9.7% had drunk alcohol daily."

Response: thank you for your comment; we have edited and highlighted it in the revised manuscript

Lines 290-291 (Table 2 Title): Change "index case" to "index cases" and 'n' to 'N'

In Table 2: Would insert ≥ in place of =/> and >=

Response: thank you for your comment; we have inserted and highlighted it in the revised manuscript

Line 294: Would be helpful to start by indicating the denominator. Might edit "Most family members (90.9%) …" as "Of the 267nfamily members, most (90.9%) …"

Response: thank you for your comment; we have edited and highlighted it in the revised manuscript

Line 294: Would add "for HIV" after "had ever been tested". and replace "not been" with "never been".

Response: thank you for your comment; we have edited and highlighted it in the revised manuscript

Line 297: Would edit “31.50%” as “31.5%” and “(196 out of 623 tested and known results).” as (196/623 cases).

Response: thank you for your comment; we have edited and highlighted it in the revised manuscript

Line 300 (Table 3 Title): “Town,Southern” needs a space

Response: thank you for your comment; we have edited and highlighted it in the revised manuscript

Table 3: Seems that the denominators for the variables vary. It might help to add the ‘N” for each variable as done for the two or add a ‘Total’ column

Response: thank you for your comment; we have edited and highlighted it in the revised manuscript

Line 302-303: Would change “only” to “the”, add (N=406) after children, and change “have” to “had”

Response: thank you for your comment; we have edited and highlighted it in the revised manuscript

Line 306: Would move the stop (.) to after Table 4

Response: thank you for your comment; we have edited and highlighted it in the revised manuscript

Line 307: “Table 1:HIV” needs a space

Response: thank you for your comment; we have edited and highlighted it in the revised manuscript

Line 308: “Ethiopia,2021” needs a space, and (n=406) should be (N=406)

Response: thank you for your comment; we have edited and highlighted it in the revised manuscript

Line 309: Would edit “In a sub-group analysis of only spousal partners” as “In a sub-group analysis of the spousal partners (N=217),…”

Response: thank you for your comment; we have edited and highlighted it in the revised manuscript

Line 310: “collection.All’ needs a space

Response: thank you for your comment; we have edited and highlighted it in the revised manuscript

Line 311: Would edit “..testing is important..” as “..testing would be important..”

Response: thank you for your comment; we have edited and highlighted it in the revised manuscript

Lines 311=313: May edit this as “A majority (98.2%) of them were ever tested, and 149 (70%) had HIV positive status.”

Response: thank you for your comment; we have edited and highlighted it in the revised manuscript

Lines 313-314: Would edit “…2 (3%) were newly identified positives.” as “two (3%) were newly identified HIV positive patients.”

Response: thank you for your comment; we have edited and highlighted it in the revised manuscript

Line314: Would edit:"From all the spousal partner respondents, .." as "Of the spousal partners, .."

Response: thank you for your comment; we have edited and highlighted it in the revised manuscript

Line 315: Would edit "… were positive and 66 (30.4%) were negative. (Table 5)" as " … were HIV positive and 66 (30.4%) were negative (Table 5).

Response: thank you for your comment; we have edited and highlighted it in the revised manuscript

Line 316: Would edit "Table 2:STIs.." as "Table 3: Sexually transmitted Infections …"

Response: thank you for your comment; we have edited and highlighted it in the revised manuscript

Line 317: Would edit "(n=217)" as "(N=217)"

Response: thank you for your comment; we have edited and highlighted it in the revised manuscript

Table 5, row 2: Would edit "had history.." as "Had history…"

Response: thank you for your comment; we have edited and highlighted it in the revised manuscript

Table 5, rows 4,5 and 6: Would add "?" at the end of each sentence

Response: thank you for your comment; we have added and highlighted it in the revised manuscript

Table 5, rows 6 and 7: Would edit the "n" as "N"

Response: thank you for your comment; we have edited and highlighted it in the revised manuscript

Line 322: Would edit: "Most (97% and 79.9%) of…" as "Most, 97% and 79.9%, of .. "

Response: thank you for your comment; we have edited and highlighted it in the revised manuscript

Lines 324-325: Would edit "The majority (505, 81.1%) and (406, 65.2%) .." as "The majority, 505 (81.1%) and 406 (65.2%) …"

Response: thank you for your comment; we have edited and highlighted it in the revised manuscript

Line 326: Would move the stop (.) to end of sentence.

Response: thank you for your comment; we have edited and highlighted it in the revised manuscript

Line 327: Table title need reframing. Would suggest "Clinical and behavioral characteristics of HIV index cases in Sodo town, Southern Ethiopia, 2021 (N=623)"

Response: thank you for your comment; we have edited and highlighted it in the revised manuscript

Table 6: Would delete "of index cases" from each row as this is redundant. The title indicates that the Table is on index cases. Space between word need to be corrects in most of the rows. Would also insert the symbol ≥ in where >= is used. Would also edit "Ever chaw chat" in the last row as Ever chawed chat.

Response: thank you for your comment; we have corrected and highlighted it in the revised manuscript

Line 329: Would edit “Associated factors with HIV seropositive status ….” as “ Factors associated with HIV seropositive status ..”

Response: thank you for your comment; we have edited and highlighted it in the revised manuscript

Line 331: Would add a comma between “known and”

Response: thank you for your comment; we have corrected and highlighted it in the revised manuscript

Lines 330-334: This is a long sentence and with important message that has not come out clearly. Please revise. Would consider describing the variables considered for the bivariate analysis and those selected based on the selection cut-off point (P<0.25).

Response: thank you for your comment; we have revised and highlighted it in the revised manuscript

Line 355: Would replace “found” with “showed”

Response: thank you for your comment; we have replaced and highlighted it in the revised manuscript

Line 341: Would replace “than” with “compared to”

Response: thank you for your comment; we have replaced and highlighted it in the revised manuscript

Line 334: Would replace “(n=406)” with “(N=406)”

Response: thank you for your comment; we have replaced and highlighted it in the revised manuscript

Table 7: Requires format editing – text font and alignment, space between words, moving the contents in the last row to a footnote.

Response: thank you for your comment; we have edited and highlighted it in the revised manuscript

Line 345: Would edit “Associated factors with HIV..” as “Factors associated with HIV…”

Response: thank you for your comment; we have edited and highlighted it in the revised manuscript

Line 346-365: Too much information in one long paragraph, and long sentences are used making it difficult to get the important messages. May help if the bivariate analysis results are given in one paragraph and those from a multivariable logistic regression analysis in a separate on. The variable that achieved significant association may just be presented with related AORs (no need of separate listing). May need to describe some of the factors in a way that is easier to get the message, like instead of “ever used a condom and had a history or symptoms of STI in the 12 months before the survey among spousal partners were associated with …” “ever using a condom and having a history or symptoms of STI in the 12 months before the survey were associated with …”. Also double check the spacing between words and punctuations.

Response: thank you for your comment; we have revised and highlighted it in the revised manuscript

Table 8: As for Table 7, stated above, requires format editing – text font and alignment, space between words, moving the contents in the last row to a footnote.

Response: thank you for your comment; we have edited and highlighted it in the revised manuscript

Discussion

Line 369: Would edit "... the seropositive…" as :… the HIV seropositive…"

Response: thank you for your comment; we have edited and highlighted it in the revised manuscript

Lines 369-370: Would edit "…(31.5%) with a 95%CI of (27.6-35.2%) .." as 31.5% (95%CI; 27.6-35.2%).

Response: thank you for your comment; we have edited and highlighted it in the revised manuscript

Line 370: Would edit "…. the index case are at high risk of HIV infection." as ".. the index cases are at high risk for HIV infection."

Response: thank you for your comment; we have edited and highlighted it in the revised manuscript

Line 371: Would edit ".. with the study.." as "..with the findings of studies.."

Response: thank you for your comment; we have edited and highlighted it in the revised manuscript

Line 372-374: Would edit "..Northwest Ethiopia, which was 30%,[8] and Zimbabwe 32.6%[10]. However, it is lower than a study conducted in South Africa 38% [11]. Moreover, it is higher than the prevalence of 22% in a study done in Malawi [12]." as "….. Northwest Ethiopia and Zimbabwe, which was 30%[8],and 32.6%[10], respectively. However, it is lower than the prevalence reported from South Africa of 38% [11], and higher than the prevalence reported from Malawi of 22% [12]."

Response: thank you for your comment; we have edited and highlighted it in the revised manuscript

Line 375-377: Would keep this with the above paragraph. Would edit "the nations." At end of the paragraph as "countries."

Response: thank you for your comment; we have edited and highlighted it in the revised manuscript

Lines 378-386: The paragraphs interpreting and discussing the results of a sub-group analysis to determine prevalence among biological children would rather be condensed in one/two paragraph(s) --- highlighting the finding, comparisons with results of other studies. Interpretation and implications of the similarities and differences …

Response: thank you for your comment; we have edited and highlighted it in the revised manuscript

Lines 387-403: Prevalence among spousal partners - same suggestions as above.

Line 405: Would use “7.7 times…” instead of “7.71 times…” at this stage (interpretation and discussion) . Same where such comparison in number terms are made (e.g., 8.1 instead of 8.06 times in line 407). It seems much easier for the reader to comprehend the message when in one decimal place. Just a suggestion!

Lines 407-411: long sentence with tortuous argument. Would break and edit as “There is no study report related to this in this specific age group and almost 90% of biological children acquire the HIV infection through vertical transmission. However, the importance of these factors is emphasized by the national PMTCT guideline of Ethiopia [7].

Response: thank you for your comment; we have edited and highlighted it in the revised manuscript

Line 411-414: Message has not come out clearly. Would edit as “In index cases who do not start ART as per the eligibility criteria, their CD4 level declines. This leads to viral replication, increase in vulnerabilities to opportunistic infections, and deterioration in patients functional status, thereby increasing the possibility of vertical viral transmission of HIV. ”

Response: thank you for your comment; we have edited and highlighted it in the revised manuscript

Line 415-420: STI and HIV: Suggest merging the two paragraphs into one. May consider suggestions given above.

Response: thank you for your comment; we have merged and highlighted it in the revised manuscript

Line 421: Would edit “This finding showed…. ” as “This finding of this study shows…..”

Response: thank you for your comment; we have edited and highlighted it in the revised manuscript

Line 422: Would edit “…index cases stayed a month…” as “…index cases who stayed for a month ..”

Response: thank you for your comment; we have edited and highlighted it in the revised manuscript

Line 423: Would delete “prevention potential”, and edit “…disclosure to spousal partners..” as “.. disclosure of their HIV positive status to their spousal partners….”

Response: thank you for your comment; we have edited and highlighted it in the revised manuscript

Line 424: Would edit “…to test ..” as “…For testing…”

Response: thank you for your comment; we have edited and highlighted it in the revised manuscript

Line 425: Would delete “an”

Response: thank you for your comment; we have deleted and highlighted it in the revised manuscript

Line 426: Would edit “create” as “creates” and remove the comma after “like”

Response: thank you for your comment; we have edited and highlighted it in the revised manuscript

Line 427: Would add “and” before “improves treatment ..”

Response: thank you for your comment; we have added and highlighted it in the revised manuscript

Line 428: Would Edit "… and suppressed viral load level that leads to …" as "… and suppresses VL level that leads to …"

Response: thank you for your comment; we have edited and highlighted it in the revised manuscript

Lines 429-430: "This finding is supported by the National Comprehensive HIV Care Guideline of Ethiopia, 2018[6]". May need to reframe this sentence to indicate that the finding of this study corroborates what is included in the guideline. What is given in guidelines supporting a research finding is difficult to comprehend.

Line 430-431: Would edit "… as eligible by eligibility criteria…" as "… on meeting the eligibility criteria…:

Response: thank you for your comment; we have edited and highlighted it in the revised manuscript

Line 432: "… didn't start immediately or did not star at all? Would specify. Would also edit: :…. with a study…" as :…. with the findings of a study…" as

Response: thank you for your comment; we have specified, edited, and highlighted it in the revised manuscript

Line 433: Would keep this paragraph with the above one. Would edit

Response: thank you for your comment; we have edited and highlighted it in the revised manuscript

Lines 433-434: Would edit :This might be since ART initiation based on the eligibility criteria leads to the index case CD4 increase and viral load level suppression that decreases transmission to his or her spousal partner.: as "This might be because ART initiation based on the eligibility criteria could lead to increase in index case CD4 count and VL suppression that decreases HIV transmission to spousal partners.

Response: thank you for your comment; we have edited and highlighted it in the revised manuscript

Line 436: Would edits "… cases had WHO status stage III.." as "… cases who were at WHO clinical stage III.."

Response: thank you for your comment; we have edited and highlighted it in the revised manuscript

Line 438: Would edit "… cases were WHO stage I or II…." as "… cases who were WHO clinical stage I or II….". Also edit "it is …" as :This finding is …" and '… Northern.. "as "…North…"

Response: thank you for your comment; we have edited and highlighted it in the revised manuscript

Line 440: Would keep this paragraph with the above one. Would edit "This is due to the fact that those who were stage" as "this could be is due to the fact that index cases who were at WHO stage …"

Response: thank you for your comment; we have edited and highlighted it in the revised manuscript

Line 441: Would edit "…decreased their CD4 levels and increased the viral load levels resulting in …"

Response: thank you for your comment; we have edited and highlighted it in the revised manuscript

Line 443: Would edit “This study revealed..” as “This study also revealed..”. Also edit “.. whose index case lived with..” as “… whose index cases lived with..”

Response: thank you for your comment; we have edited and highlighted it in the revised manuscript

Line 449: Would delete “… of the study”

Response: thank you for your comment; we have deleted and highlighted it in the revised manuscript

Line 450: Would replace “Since ..” with “Because…”

Response: thank you for your comment; we have replaced and highlighted it in the revised manuscript

Line 451: Would consider hanging “… a separate sub-group analysis..” to “being able to address both outcomes in one study…”

Response: thank you for your comment; we have edited and highlighted it in the revised manuscript

Line 452: Would keep this paragraph with the above. Would consider editing the paragraph as “Family members whose index cases did not disclosed their status or had follow-up outside of Sodo Town were not included in the study. Obstetric and as well as modes of transmission other than MTCTwere excluded as the study focused on biological factors.”

Response: thank you for your comment; we have edited and highlighted it in the revised manuscript

Line 453: Would keep conclusion in bolded lower case.

Response: thank you for your comment; we have edited and highlighted it in the revised manuscript

Line 457: Would edit “… high..late” as “… high. Late …”

Response: thank you for your comment; we have edited and highlighted it in the revised manuscript

Line 459: Would edit “… the major factors of seropositive…” as “… the major factors associated with HIV seropositive status”

Response: thank you for your comment; we have edited and highlighted it in the revised manuscript

Line 460: Would edits “Whereas among …” as “In the ….”. Also remove the hyphen in “HIV-history”

Response: thank you for your comment; we have edited and highlighted it in the revised manuscript

Line 461: Would edit “… and from the index case side,” as “whereas among the index cases, …”. Also remove “of’ in “…disclosure of status ‘’

Response: thank you for your comment; we have edited and highlighted it in the revised manuscript

Line 463: Would edit “… known > 10 years..” as “… known of > 10 years

Response: thank you for your comment; we have edited and highlighted it in the revised manuscript

Lines 463-465: Would revise as "In the context of Sodo Town, comprehensive HIV prevention, care, and treatment services need be promoted and implemented among family members of the index cases to increase the achievements of program targets in HIV epidemic control.

Response: thank you for your comment; we have revised and highlighted it in the revised manuscript

Acknowledgment:

Availability of data and materials

Funding

References

Suggest that the authors review and revise each reference as required to ensure that PLoS (Vancouver) Styles are met, There is also a need to meticulously revise the references taking care of text case, capitalization of proper names, punctuations, etc.

---

6. PLOS authors have the option to publish the peer review history of their article (what does this mean?). If published, this will include your full peer review and any attached files.

If you choose "no", your identity will remain anonymous but your review may still be made public.

Do you want your identity to be public for this peer review? For information about this choice, including consent withdrawal, please see our Privacy Policy.

Reviewer: Yes**.** Sileshi Lulseged

[NOTE: If reviewer comments were submitted as an attachment file, they will be attached to this email and accessible via the submission site. Please log into your account, locate the manuscript record, and check for the action link "View Attachments". If this link does not appear, there are no attachment files.]

While revising your submission, please upload your figure files to the Preflight Analysis and Conversion Engine (PACE) digital diagnostic tool, https://pacev2.apexcovantage.com/. PACE helps ensure that figures meet PLOS requirements. To use PACE, you must first register as a user. Registration is free. Then, login and navigate to the UPLOAD tab, where you will find detailed instructions on how to use the

tool. If you encounter any issues or have any questions when using PACE, please email PLOS at figures@plos.org. Please note that Supporting Information files do not need this step.

In compliance with data protection regulations, you may request that we remove your personal registration details at any time. (Remove my information/details). Please contact the publication office if you have any questions.

Filename not specified. Reply Forward
