# Supplementary material for: Prevalence of HIV seropositive status and associated factors among family members of index cases of antiretroviral clinical attendants in Sodo Town, Southern Ethiopia
Source: PLoS One. 2023 Feb 13;18(2):e0280571. doi: 10.1371/journal.pone.0280571 (PMC9924999; doi:10.1371/journal.pone.0280571)
Supplement: Attachment — Submitted filename: Review Comments.docx [file pone.0280571.s002.docx]

**Manuscript #:** PONE-D-22-04620

**Title:** Seropositive Status and Associated Factors among Family Members of Index Cases of ART clinic Attendants in Sodo Town, Southern Ethiopia

**Comments to the Author**

1. Is the manuscript technically sound, and do the data support the conclusions?

The manuscript must describe a technically sound piece of scientific research with data that supports the conclusions. Experiments must have been conducted rigorously, with appropriate controls, replication, and sample sizes. The conclusions must be drawn appropriately based on the data presented.

Reviewer #1: Yes

2. Has the statistical analysis been performed appropriately and rigorously?

Reviewer #1: Yes

3. Have the authors made all data underlying the findings in their manuscript fully available?

The [PLOS Data policy](http://www.plosone.org/static/policies.action#sharing) requires authors to make all data underlying the findings described in their manuscript fully available without restriction, with rare exception (please refer to the Data Availability Statement in the manuscript PDF file). The data should be provided as part of the manuscript or its supporting information, or deposited to a public repository. For example, in addition to summary statistics, the data points behind means, medians and variance measures should be available. If there are restrictions on publicly sharing data—e.g. participant privacy or use of data from a third party—those must be specified.

Reviewer #1: Yes

4. Is the manuscript presented in an intelligible fashion and written in standard English?

PLOS ONE does not copyedit accepted manuscripts, so the language in submitted articles must be clear, correct, and unambiguous. Any typographical or grammatical errors should be corrected at revision, so please note any specific errors here.

Reviewer #1: No

5. Review Comments to the Author

Please use the space provided to explain your answers to the questions above. You may also include additional comments for the author, including concerns about dual publication, research ethics, or publication ethics. (Please upload your review as an attachment if it exceeds 20,000 characters)

**General Comments:**

focused objective and has generated the required data and performed an appropriate analysis of the data and interpretation of the results. It has generated conclusions warranted by the data. However, the language of the submitted manuscript for from adequate, both from the perspective of clearly conveying what is intended and quality standards of scientific publications. Here, review comments are provided that the authors may wish to consider improving the quality of the manuscript, particularly in terms of content organization, formatting, and language to make the manuscript potentially publishable in PLOS.

Authors need to use the ‘PLoS Guidelines to Authors’ in the formatting (line spacing, text alignment) as well as in organizing the content in the different sections of the manuscript. Please note that some of the comments given in the different sections below clearly indicate this.

The manuscript requires heavy language editing – authors may wish to enlist support from a language editor. Much language editing in the manuscript is done as part of this review (please see below comments in each section)

**Specific comments:**

**Title/Title Page**

The title should be written in sentence case (capitalize only the first word of the title and proper names); expand ART to Antiretroviral Treatment

Line 1: Missing space between Seropositive and Status

Line 3: Missing space between words. Correct CLINICATTENDANTSINSODO to Clinic Attendants in Sodo

Line 4: Would edit Abebe ^2^Dereje as read Abebe^2^, Dereje

Line 5: Would remove the extra comma between Addis^4^ and Muluken

Lines 6-9: Would edit the affiliation for each author (include department, University, or organization affiliation, and its location, including city, state/province, and country

Would add on this page: first author’s e-mail address followed by name initials in parenthesis

On this page: also indicate authors’ contribution(s) using symbols as given in PLoS One “Title, Author, Affiliation Formatting Guidelines”.

**Abstract:**

Lines 30-31: the sentence needs editing; suggest "... transmitted through … sexual contact with an infected partner and babies born to mothers infected with the virus.”

Lines 33-35: The sentence is the title repeated as is; suggest restating this in a form of an objective ... Like “This study aimed at identifying prevalence and determinants of HIV infection among family members of index cases on antiretroviral treatment (ART).”

Line 38: Would Insert HIV between “of” and “index”

Line 39: “.. collected data ……” on what ?

Line 39: Would add space after the stop after data.

Line 39: Would replace “investigate” with “identify”

Line 40: Helpful to add the expanded form for AOR upfront here so that we use the acronym in the rest f the manuscript; i.e., adjusted odds ratio (AOR)

Line 41: Same here. 95% Confidence interval (CI)

Lines 41: The P in P-values is written in upper case as is and italicised (*P*). Please do this across the manuscript.

Lines 41-42: last part of the sentence requires editing. Suggest changing “----used to declare the cut-off point in determining the level of significance” to “---- used as a cut-off point to determine the level of statistical significance of point estimate.”

Line 45: Would edit “----- revealed that 31.5% with 95%CI (27.6-35.2%)’ as “----- revealed that 31.5% (95%CI: 27.6-35.2%); need to use same formant across the manuscript.

Lines 46-47: Would edit: (…11.1% with 95%CI (8.4–14.5%..) as (…11.1% (95%CI: 8.4–14.5%)

Line 47: Would edit: “…and 69.6% with 95%CI (63.1–75.6%..)” as “….. and 69.6% (95%CI: 63.1–75.6%)

Line 50: Would insert ‘of’ between “level” and “350”

Line 51: Would edit “….. significant with ….” as “….. significantly associated with …”

Line 54: Would edit: “…. infection since known …”, may remove ‘since known’ as this does not add an y useful information.

Line 59: Would change ‘should be’ to 'might be' or 'need to'; one needs to avoid strong words like should in conclusions/recommendations.

**Introduction**

Line 60: May consider editing as “HIV infection, HIV seropositive, biological children, spousal partners, index case,”.

Line 63: Definition is incomplete, and the language requires editing. Option: "Index testing is a voluntary process where counsellors or health care providers ask a newly diagnosed HIV positive patient or an HIV positive patient already accessing HIV treatment to list all partners who could have been exposed to HIV through them, the index cases" [1].

Line 57: Just use the acronym ‘ICT’; already used at first enter above with the expanded forms.

Line 67: Insert space “ ….models of testing:(1)Facility…..” so that it reads “ ….models of testing (1) Facility…..”

Line:67: Would edit “… (1)Facility Based ICT …” as “ ……. (1) Facility-based ICT …”

Line 68; Would edit “…. index case and gets the names ..” to read “…. index cases on ART at the health facility, and gets the names .. “

Line 69: Would replace ‘old’ with “of age’

Line 69: Would Insert a comma after ‘old’ and delete “in Antiretroviral Treatment (ART) service giving facility.

Line 69-72: Sentence after ‘(2)’ needs revision, as it has many grammatic errors; suggest: “community-based ICT: a counsellor traces sexual partner(s) of index cases and their biological children who are under 15 years of age through home-to-home visits, offers HTS, and links newly identified People Living with HIV (PLHIV) to ART services [1].

Line 75. Would add ‘year’ at the end of the sentence.

Line 76. Paragraph starting with this line is a continuation of the above; Would keep this para with the above.

Line 76: Would edit “….. PLHIV were lived and …” as “…. PLHIV were living in 2021, and of which ….”

Line 77: In “… transformative and ---” would add ‘a’ before transformative.

Line 78: Would edit “ …. expansion of ART indicating that from total of ….”as “…… as indicated by the total number of …”

Line 79: Would edit “…. sixteen million (16 million) …” as “..16 million…”

Line 79: Would need space between treatment and the citation in“…. on treatment[2] ….”. Would do this consistently all along.

Line 80: Would replace ”…among which …” by “… of which ….”

Line 82: Would keep this paragraph starting with line with the one above paragraph.

Line 82: Would delete ‘also”

Line 82. Would replace …among which….” With “… of which …”

Line 83: Would edit “… of age, with 13,000 …” to read “ of age. There were 13,000….”

Line 83: Would edit “ …. under 15 years old.” as “….. under 15 years of age. ….”

Line 84: Would edit “PLHIVs” should be “PLHIV”

Line 86: Would merge the paragraph starting with “according to the Ethiopian ….” with the above paragraph

Line 87: Would edit “….was 0.9 percent (0.7 percent ….” as “… was 0.9% (0.7% …..”; and be consistent all along.

Line 88: Would replace “With this general prevalence, the current epidemiology of HIV is heterogeneous, with ….” with 'HIV/AIDS in Ethiopia is regularly categorized as “generalized“ among the adult population, with …….”

Line 90: Would replace “…. Geographical ….” with ““…. Geographic ….”

Line 90: Would insert a reference (e.g., the Ethiopian Population-based HIV Impact Assessment 2020) at end of the line (after subgroups)

Line 92: Would insert after “below 0.1” a reference (e.g., EPHI, Mini EDHS 2018/19).

Line 92: Would replace ‘Somalia” with ‘Ethiopian Somali Region”

Line 97:Paragraph starting with this is redundant, repeating what is covered in the above paragraph. Would delete.

Line 101: Would insert HIV between universal and testing and edit “ …. testing strategy …”, as“…..test and treat strategy ….”

Line 101: Would edit “…. testing all individuals as a mass ……” as “ …. testing all individuals in the population who were at risk …”

Line 102: Would edit :…. index case clients are one of these areas [6] …” as“….index cases as a target population for this strategy [6].

Line 104: Would keep paragraph starting with this line with the above one.

Line 105: Would edit “….and Family-Based Index Case Testing ….” as“…..Family-based Index Case Testing ……”

Line 105: Would edit “……. or Index Case Testing (ICT) strategy ….” as “……. or the ICT strategy ….”

Line 107: Would edit “…..under-15-year old…” to“…..under 15-year-old…”

Line 107: Would edit: “…… biological children to break the chain of ……. “ as “…… biological children. This breaks the chain of ……. “ NB. This breaks a long sentence.

Line 108: Would edit: “….. who have been exposed to HIV …”as“….. who are exposed to HIV …”

Line 108: Would edit “….. to HIV to give preventive …..” as “to HIV, provide preventive ….”

Line 109: Would edit “…… services for negative and, ….” as “…… services for those testing HIV-negative, and, ….

Line 109: Would edit “…. if positive, linkage and treatment that increase case detection and attain ….” as “. if positive, link them to cared and treatment services, a cascade that will contribute to attaining the..”

Lines 110-111: Would edit “…. while family members have an ongoing risk of contracting HIV.” as “….. exposing the family members to an ongoing risk of contracting HIV.”

Line 111: Would replace “So” with “Therefore”

Lines 112-113: Would edit “….under 15 years of biological children[1].” as “….under 15-years old biological children[1].”

Line 117: The paragraph starting with this line would rather be kept with the above one.

Line 120-122: Would replace “Testing for HIV and linking to care for those identified by the families of the index cases will improve the health outcomes of the seropositive family members[5].” with “Testing for HIV and linking those who are positive to care and treatment services will improve the health outcomes of the seropositive family members[5].

“Line 123: Would replace ‘But’ with :Nevertheless’

Line 123: Would edit “HIV positive” as “HIV-positive”; and do this consistently across the manuscript

Lines 123-127: “…people for HIV testing and associated factors among ART clinic attendants at Felege Hiwot Referral Hospital, Northwest Ethiopia, reveals that 25.8% of index cases were not tested. Even though there were a significant number of family members not tested in this study, 30% of family members were seropositive[8].” requires editing; Would consider “……. ART clinic attendants at a referral hospital in Northwest Ethiopia revealed that many children and adults were traced and tested for HIV. Yet, there were many untested partners, children and other individuals living with index cases who were not tested.

Lines 128-130: Would delete this para; already addressed in the paragraphs above.

Line131: Paragraph starting with this line would rather be kept with paragraph above ending with ref #8.

Line 131: Would edit “…. no study was conducted …” as “… no similar study was conducted …”

‘Line 134: Would replace ‘’would’ with “will”

Lines 134-136: “ ….. the family members' information in routine clinical practice and could facilitate ending the HIV/AIDS epidemic vision in 2030.” requires editing; would suggest: :… 'HIV serostatus information on family members of index cases, which will contribute to improving HIV care and testament services and epidemic control in Ethiopia'.

**Methods**

Line 138: Usually written as ‘Materials and Methods” or just “Methods”

Line 142: Would expand SNNPR. Southern Nations, Nationalities, and Peoples Region (SNNPR).

Line 142: Would edit “… Sodo Town has been…” as “… Sodo Town is …”

‘Line 143: Would replace ‘3’ with ‘three’

Line 143: Would edit as “ … kebele (lowest administrative entity)”

Line 144: Would insert reference for “According to the 2021 population and housing census projection	[ ]. …”

Line 146: Would replace ‘3’ with ‘three’

Line 146: Would edit: “.. service-giving facilities ….” as “.. service-delivery facilities ….”

Line 147: Would replace ‘2” with ‘two” and ‘1’ with ‘one’

Line 150: Would replace sentence with “The study was a community-based cross-sectional study”

Line 151: Would edit as “source population”

Line 152-153: The source population of this study seems all patients coming to the health facilities involved in this study. May consider editing the sentence accordingly.

Line 155-156: The study subjects seem “All index cases coming at ART clinics at the study health facilities and their family members.”. May consider editing the sentence accordingly.

Line 162-163: May edit as “An index case living in Sodo who at least had one biological child aged 2–14 years or a spousal partner”.

Line 164-165: Would delete ‘living in Sodo’ as it is included in the above sentence.

Line 169: Would add after illness “or not willing to participate.”

Line 171-172: May consider editing the sentence as “The ART clinics at all three health facilities in Sodo Town (Sodo Health, Sodo Teaching and Referral Hospital, and Sodo Christian General Hospital) were included as study sites.

Line 173: Would edit “… HIV positive clients …” as “… HIV-positive patients…”

Line 173: Would delete “(COA)”, not a conventional acronym/abbreviation.

Line 175: Would edit “…and among these 2,213…” as “…. And, among these, 2,213…”

Line 175: Would delete ‘old’

Line 176: May change ‘Primarily’ to ‘As a first step’

Line 176: Would replace “Partner and Family-Based Index Case testing” with “PFBICT’ – you already have this in line 105 above.

Line 177: Would delete ‘only’ in :.. clients only living ..”

Line 177: Would edit “… in Sodo Town, and there were..” as “… in Sodo Town, which were…..”

Line 177: Would edit “Secondly, by using HIV….” as “By using HIV….”

Line 178: Would edit “….we identified 834…”…. as we then identified 834…”

Line 178: Would edit “….. Then, from the total …..” as “Of the total ….”

Line 180: Would edit “The ART code numbers of facilities were used to identify each facility.….” as “The unique ART code numbers given by the national program to the facilities were used to identify each study facility.”

Line 184: Would edit “… By considering this from 723 index clients,…” as : Of 723 index patients, …”

Line 184: Would edit “..to not..” as “… not to…”

Line 186: Would edit “…. and selected them as total index cases.”

Line 187: Would edit “…. for a total…” as “… and, as a result, had a total …”

Line 196: Would remove the “hyphen”

Line 198: Would remove the “hyphen”

Line 199: Would edit “VL” as “viral load (VL)”

Line 200: Would remove the “hyphen”

Line 202: Would insert a “comma: after ‘sexual partners’

Line 203: Would remove the “hyphen:

Lines 209-210: “After addressing the disclosure issue, data collectors were appointed to index cases with their family members for visiting home.” requires editing. Would suggest: “Following disclosure of HIV status by the index cases, data collectors fixed appointments with them and their families for home visits.”

Line 210: Would edit “…Then, during visiting, …:” as :.. At the start of the visit, …"

Line 211: Would edit “… consent for spousal partners…” as “… consent obtained from spousal partners…”

Lines 211-212: Would edit “… and assent for biological children was taken….” as “Consent for all biological children was obtained from the parents, and assent was obtained from children 12-14 years of age…”

Line 212: Would edit “Positive serostatus participants were only interviewed, but …” as “In those who were HIV-positive, only interviews were adminste4ed by the investigators, but ...”

Line 213: Would edit “…testing and interview were undergone simultaneously…” as “….. the participants were interviewed and tested for HIV.”

Line 213: Would delete “or guardians”, otherwise, the word biological children should be removed from the manuscript and parent/guardian used consistently all along.

Lines 214-215: “The testing procedure was strictly followed by the current national testing algorithm” requires editing; would suggest “The testing procedure strictly followed the current national testing algorithm.”

Lines 215-216: “For the data of the index case, unique ART numbers were used to review secondary data and link it with the primary data of their family members.” requires editing; Would suggest “Unique ART numbers were used to link primary index case data with that of the family members and with their secondary data in patients' records for analysis.”

Lines 218-231:cosnsitentlu use colons (:) after the bolded words to be defined. Would remove the “hyphens”

Line 218: Would delete “old”

Line 222: Would edit “…..algorithmic test” as :….. algorithm”

Line 225-231: Would replace equal ‘=’ with “comma“

Line 225: Would edit “…. share his status to his family members…” as “…. share their status to their family members…”

Line 226: Would edit “…one month, late …” as “…one month, and late …”

Line 228: Would replace “&’ with and.

Line 229: Would remove ‘=’ before immediate/

Line 234: Would edit: “…. The English version was translated …” as “…. The English version of the questionnaire was translated …”

Line 234: Edit “… to the….” as “… into ….”

Lines 235-236: “… for gathering information …..” as “….to collect data ….”

Line 236: “Five percent of the questioners were pre-tested in the Boditi ….” .. not clear. Is it to say ‘the questionnaire was pre-tested by the data collectors and the supervisors in 5% of the sample at Boditi Health Center, a site similar to the study health facilities?

Line 237: Would edit “Then it was modified or contextualized.” as “The results were used to modify the questionnaire as appropriate.”

Line 239-240: Requires editing. Would suggest “… two-day training introduced the objective of the study, familiarized data collectors with the questionnaire, the sampling procedure and interviewing techniques, and the HIV testing procedures …”

Line 240-242: Would delete “The data collectors and supervisors participated in pre-testing of the questionnaire for its understanding, which was not included in the study, and the result was used to correct unclear ideas and statements.” As this is incorporated covered as in the edited above.

Line:343-243: You may edit “During data collection, the supervisors….” as “The supervisors ….”

Line 245: Requires editing. Would suggest “After data collection, the collected information was” is replaced by “The collected data were ….”

Line 235: Would delete “its: and in line 146 delete the “s” in principal investigators.

Line 250-259: This section requires revision. Would suggest revising as “EPI data 4.2 statistical software was used for data entry, cleaning, and coding of the variables. The data were exported to SPSS version 25 for analysis. Descriptive statistics were used to summarize the data and check for the distribution of the study variables. A bivariate analysis was done by using a binary logistic regression. A *P*-value of 0.25 was used as a cut-off point to select candidate variables for multivariate logistics regression, the analysis used to determine the association between the independent and dependent variables having controlled for confounding effects. AOR with 95%CI was used to determine the level association. The Hosmer and Lemeshow's goodness of fit test was used to check for model fitness. A variance inflation factor (VIF) or tolerance test was done to check for correlation. A *P*-value of 0.05 was used as a cut-off point to determine statistical significance and the results were presented in narratives and tables.”

Line 263: Would delete “in decentralized order”

Line 265: Would edit “…. assent for biological children from their parents….” as “…consent from partners for their biological children plus assent from children aged 12-14 years …”

Lines 266-267: Would edit “…. The testing and….” as “…. HIV testing and….”

Line 267: Would replace“…. separate ..” with”…. secluded	…”

Line 268: Would edit “…. and positives were ….” as “…. and those who were positives were ….”

**Results**

Line 272: Would edit “… “…. interviewed with a response rate of 94%.” as ““…. Interviewed, which was a response rate of 94%.”

Line 273: Would edit “…While the majority were biological children (406, 65.2%),..” as While the majority, 406 ( 65.2%), were biological children…”

Lines 273-274: Would edit “… nearly one-third (217, 34.8%) “ as “… nearly one-third, 217 (34.8%), ….”

Lines 274-275: Would edit: “The mean age of biological children was 8.9 ± 3.66 and the spousal partner was 36.9 ± 9.26 years. …” as “The mean (SD) age of biological children was 8.9 (± 3.66) and of the spousal partner was 36.9 (± 9.26) years.

Lines 276- 277: “… had Frist percentile wealth status”; is this to mean a wealth status in the first quartile?

Lines 277:278: Need to watch consistent use of comas or no commas in numbers (e, g,, 1072 and 2,932). Also across the manuscript

Lines 280-281 (Table 1): Would edit as “…. members of index cases in Sodo Town, Southern Ethiopia, 2021(N=623).* You may move the content in the last row of the table and add it as a footnote starting with an asterisk (*) to link it with the title.

Table 1: Do not understand the categories for the wealth “Wealth Index”. I am wondering whether these are quartiles or centiles.

Table 1: Would insert ≥ symbol (from the Symbols menu) in place of >=

Table 1 (narrative in the last row) : “expected651” needs a space. Would remove the comma in “… cases, to ….” as “N’ we have 623 in the title and 662 here? Edit “…. used for to determine non-response rate.” as “…. used as the denominator to determine non-response rate.”

Line 283: Would edit “… index case.” as “…index cases.”

Line 284: Would provide the denominator upfront here like “Of the 217 spousal partners, nearly ….

Line 288: Would edit “… with their spousal partner.” as “… with their spousal partners.”

Lines 288-289: Would edit as “While the majority (78.8%) had never smoked tobacco, 70% had chawed chat, 9.7% had drunk alcohol daily.”

Lines 290-291 (Table 2 Title): Change “index case” to “index cases” and ‘n’ to ’N’

In Table 2: Would insert ≥ in place of =/> and >=

Line 294: Would be helpful to start by indicating the denominator. Might edit “Most family members (90.9%) …” as “Of the 267nfamily members, most (90.9%) …”

Line 294: Would add “for HIV” after “had ever been tested”. and replace “not been” with “never been”.

Line 297: Would edit “31.50%” as “31.5%” and “(196 out of 623 tested and known results).” as (196/623 cases).

Line 300 (Table 3 Title): “Town,Southern” needs a space

Table 3: Seems that the denominators for the variables vary. It might help to add the ‘N” for each variable as done for the two or add a ‘Total’ column

Line 302-303: Would change “only” to “the”, add (N=406) after children, and change “have” to “had”

Line 306: Would move the stop (.) to after Table 4

Line 307: “Table 4:HIV” needs a space

Line 308: “Ethiopia,2021” needs a space, and (n=406) should be (N=406)

Line 309: Would edit “In a sub-group analysis of only spousal partners” as “In a sub-group analysis of the spousal partners (N=217),…”

Line 310: “collection.All’ needs a space

Line 311: Would edit “..testing is important..” as “..testing would be important..”

Lines 311=313: May edit this as “A majority (98.2%) of them were ever tested, and 149 (70%) had HIV positive status.”

Lines 313-314: Would edit “…2 (3%) were newly identified positives.” as “two (3%) were newly identified HIV positive patients.”

Line 314: Would edit:”From all the spousal partner respondents, ..” as “Of the spousal partners, ..”

Line 315: Would edit “… were positive and 66 (30.4%) were negative. (Table 5)” as “ … were HIV positive and 66 (30.4%) were negative (Table 5).

Line 316: Would edit “Table 5:STIs..” as “Table 5: Sexually transmitted Infections …”

Line 317: Would edit “(n=217)” as “(N=217)”

Table 5, row 2: Would edit “had history..” as “Had history…”

Table 5, rows 4,5 and 6: Would add “?” at the end of each sentence

Table 5, rows 6 and 7: Would edit the “n” as “N”

Line 322: Would edit: “Most (97% and 79.9%) of …” as “Most, 97% and 79.9%, of .. “

Lines 324-325: Would edit “The majority (505, 81.1%) and (406, 65.2%) ..” as “The majority, 505 (81.1%) and 406 (65.2%) …”

Line 326: Would move the stop (.) to end of sentence.

Line 327: Table title need reframing. Would suggest “Clinical and behavioral characteristics of HIV index cases in Sodo town, Southern Ethiopia, 2021 (N=623)”

Table 6: Would delete “of index cases” from each row as this is redundant. The title indicates that the Table is on index cases. Space between word need to be corrects in most of the rows. Would also insert the symbol ≥ in where >= is used. Would also edit “Ever chaw chat” in the last row as Ever chawed chat.

Line 329: Would edit “Associated factors with HIV seropositive status ….” as “ Factors associated with HIV seropositive status ..”

Line 331: Would add a comma between “known and”

Lines 330-334: This is a long sentence and with important message that has not come out clearly. Please revise. Would consider describing the variables considered for the bivariate analysis and those selected based on the selection cut-off point (P<0.25).

Line 355: Would replace “found” with “showed”

Line 341: Would replace “than” with “compared to”

Line 334: Would replace “(n=406)” with “(N=406)”

Table 7: Requires format editing – text font and alignment, space between words, moving the contents in the last row to a footnote.

Line 345: Would edit “Associated factors with HIV..” as “Factors associated with HIV…”

Line 346-365: Too much information in one long paragraph, and long sentences are used making it difficult to get the important messages. May help if the bivariate analysis results are given in one paragraph and those from a multivariable logistic regression analysis in a separate on. The variable that achieved significant association may just be presented with related AORs (no need of separate listing). May need to describe some of the factors in a way that is easier to get the message, like instead of “ever used a condom and had a history or symptoms of STI in the 12 months before the survey among spousal partners were associated with …” “ever using a condom and having a history or symptoms of STI in the 12 months before the survey were associated with …”. Also double check the spacing between words and punctuations.

Table 8: As for Table 7, stated above, requires format editing – text font and alignment, space between words, moving the contents in the last row to a footnote.

**Discussion**

Line 369: Would edit “... the seropositive…” as :… the HIV seropositive…”

Lines 369-370: Would edit “…(31.5%) with a 95%CI of (27.6-35.2%) ..” as 31.5% (95%CI; 27.6-35.2%).

Line 370: Would edit “…. the index case are at high risk of HIV infection.” as “.. the index cases are at high risk for HIV infection.”

Line 371: Would edit “.. with the study..” as “..with the findings of studies..”

Line 372-374: Would edit “..Northwest Ethiopia, which was 30%,[8] and Zimbabwe 32.6%[10]. However, it is lower than a study conducted in South Africa 38% [11]. Moreover, it is higher than the prevalence of 22% in a study done in Malawi [12].” as “….. Northwest Ethiopia and Zimbabwe, which was 30% [8], and 32.6% [10], respectively. However, it is lower than the prevalence reported from South Africa of 38% [11], and higher than the prevalence reported from Malawi of 22% [12].”

Line 375-377: Would keep this with the above paragraph. Would edit “the nations.” At end of the paragraph as “countries.”

Lines 378-386: The paragraphs interpreting and discussing the results of a sub-group analysis to determine prevalence among biological children would rather be condensed in one/two paragraph(s) --- highlighting the finding, comparisons with results of other studies. Interpretation and implications of the similarities and differences …

Lines 387-403: Prevalence among spousal partners - same suggestions as above.

Line 405: Would use “7.7 times…” instead of “7.71 times…” at this stage (interpretation and discussion) . Same where such comparison in number terms are made (e.g., 8.1 instead of 8.06 times in line 407). It seems much easier for the reader to comprehend the message when in one decimal place. Just a suggestion!

Lines 407-411: long sentence with tortuous argument. Would break and edit as “There is no study report related to this in this specific age group and almost 90% of biological children acquire the HIV infection through vertical transmission. However, the importance of these factors is emphasized by the national PMTCT guideline of Ethiopia [7].

Line 411-414: Message has not come out clearly. Would edit as “In index cases who do not start ART as per the eligibility criteria, their CD4 level declines. This leads to viral replication, increase in vulnerabilities to opportunistic infections, and deterioration in patients functional status, thereby increasing the possibility of vertical viral transmission of HIV.”

Line 415-420: STI and HIV: Suggest merging the two paragraphs into one. May consider suggestions given above.

Line 421: Would edit “This finding showed…. ” as “This finding of this study shows…..”

Line 422: Would edit “…index cases stayed a month…” as “…index cases who stayed for a month ..”

Line 423: Would delete “prevention potential”, and edit “…disclosure to spousal partners..” as “.. disclosure of their HIV positive status to their spousal partners….”

Line 424: Would edit “…to test ..” as “…For testing…”

Line 425: Would delete “an”

Line 426: Would edit “create” as “creates” and remove the comma after “like”

Line 427: Would add “and” before “improves treatment ..”

Line 428: Would Edit “… and suppressed viral load level that leads to …” as “… and suppresses VL level that leads to …”

Lines 429-430: “This finding is supported by the National Comprehensive HIV Care Guideline of Ethiopia, 2018[6]”. May need to reframe this sentence to indicate that the finding of this study corroborates what is included in the guideline. What is given in guidelines supporting a research finding is difficult to comprehend.

Line 430-431: Would edit “… as eligible by eligibility criteria…” as “… on meeting the eligibility criteria…:

Line 432: “… didn’t start immediately or did not star at all? Would specify. Would also edit: :…. with a study…” as :…. with the findings of a study…” as

Line 433: Would keep this paragraph with the above one. Would edit

Lines 433-434: Would edit :This might be since ART initiation based on the eligibility criteria leads to the index case CD4 increase and viral load level suppression that decreases transmission to his or her spousal partner.: as “This might be because ART initiation based on the eligibility criteria could lead to increase in index case CD4 count and VL suppression that decreases HIV transmission to spousal partners.

Line 436: Would edits “… cases had WHO status stage III..” as “… cases who were at WHO clinical stage III..”

Line 438: Would edit “… cases were WHO stage I or II….” as “… cases who were WHO clinical stage I or II….”. Also edit “it is …” as :This finding is …” and ‘… Northern.. ”as “…North…”

Line 440: Would keep this paragraph with the above one. Would edit “This is due to the fact that those who were stage” as “this could be is due to the fact that index cases who were at WHO stage …”

Line 441: Would edit “…decreased their CD4 levels and increased the viral load levels resulting in …”

Line 443: Would edit “This study revealed..” as “This study also revealed..”. Also edit “.. whose index case lived with..” as “… whose index cases lived with..”

Line 449: Would delete “… of the study”

Line 450: Would replace “Since ..” with “Because…”

Line 451: Would consider hanging “… a separate sub-group analysis..” to “being able to address both outcomes in one study…”

Line 452: Would keep this paragraph with the above. Would consider editing the paragraph as “Family members whose index cases did not disclosed their status or had follow-up outside of Sodo Town were not included in the study. Obstetric and as well as modes of transmission other than MTCT were excluded as the study focused on biological factors.”

Line 453: Would keep conclusion in bolded lower case.

Line 457: Would edit “… high..late” as “… high. Late …”

Line 459: Would edit “… the major factors of seropositive…” as “… the major factors associated with HIV seropositive status”

Line 460: Would edits “Whereas among …” as “In the ….”. Also remove the hyphen in “HIV-history”

Line 461: Would edit “… and from the index case side,” as “whereas among the index cases, …”. Also remove “of’ in “…disclosure of status ‘’

Line 463: Would edit “… known > 10 years..” as “… known of > 10 years

Lines 463-465: Would revise as “In the context of Sodo Town, comprehensive HIV prevention, care, and treatment services need be promoted and implemented among family members of the index cases to increase the achievements of program targets in HIV epidemic control.

**Acknowledgment:**

***Availability of data and materials***

***Funding***

**References**

Suggest that the authors review and revise each reference as required to ensure that PLoS (Vancouver) Styles are met, There is also a need to meticulously revise the references taking care of text case, capitalization of proper names, punctuations, etc.

6. PLOS authors have the option to publish the peer review history of their article ([what does this mean?](https://journals.plos.org/plosone/s/editorial-and-peer-review-process#loc-peer-review-history)). If published, this will include your full peer review and any attached files.

If you choose “no”, your identity will remain anonymous but your review may still be made public.

**Do you want your identity to be public for this peer review?** For information about this choice, including consent withdrawal, please see our [Privacy Policy](https://www.plos.org/privacy-policy).

Reviewer:  Yes**.** Sileshi Lulseged

[NOTE: If reviewer comments were submitted as an attachment file, they will be attached to this email and accessible via the submission site. Please log into your account, locate the manuscript record, and check for the action link "View Attachments". If this link does not appear, there are no attachment files.]

While revising your submission, please upload your figure files to the Preflight Analysis and Conversion Engine (PACE) digital diagnostic tool, [https://pacev2.apexcovantage.com/](https://pacev2.apexcovantage.com/). PACE helps ensure that figures meet PLOS requirements. To use PACE, you must first register as a user. Registration is free. Then, login and navigate to the UPLOAD tab, where you will find detailed instructions on how to use the tool. If you encounter any issues or have any questions when using PACE, please email PLOS at [figures@plos.org](mailto:figures@plos.org). Please note that Supporting Information files do not need this step.

*In compliance with data protection regulations, you may request that we remove your personal registration details at any time.* [*(Remove my information/details)*](https://www.editorialmanager.com/pone/login.asp?a=r)*. Please contact the publication office if you have any questions.*

| **Filename not specified.** | Reply Forward |
| --- | --- |
