# Supplementary material for: Prevalence of HIV seropositive status and associated factors among family members of index cases of antiretroviral clinical attendants in Sodo Town, Southern Ethiopia
Source: PLoS One. 2023 Feb 13;18(2):e0280571. doi: 10.1371/journal.pone.0280571 (PMC9924999; doi:10.1371/journal.pone.0280571)
Supplement: Attachment — Submitted filename: response to reviwer comment.docx [file pone.0280571.s005.docx]

Reviewer #1: There are grammatic corrections that need to made by authors and section that need to be revised (e,g. references). These are highlighted in the revised manuscript with sticky notes - uploaded as an attachment.

Response: Thank you for your comment. We have corrected and highlighted it in the revised manuscript.
