# Supplementary material for: Prevalence of HIV seropositive status and associated factors among family members of index cases of antiretroviral clinical attendants in Sodo Town, Southern Ethiopia
Source: PLoS One. 2023 Feb 13;18(2):e0280571. doi: 10.1371/journal.pone.0280571 (PMC9924999; doi:10.1371/journal.pone.0280571)
Supplement: Attachment — Submitted filename: Response to the reviewer R3.docx [file pone.0280571.s006.docx]

**Response to the reviewer:** thank you for your meticulous observation and comment. In this study, a sample size was determined using the single population proportion formula by assuming a proportion of 50%, a margin of error of 5%, a 95% confidence interval, and a non-response rate of 10%. The calculated sample size was 423. However, a total of 651 eligible index cases of HIV were identified from the registry, so we used the census method to recruit all study participants.
